# Preparation of von Hippel-Lindau (VHL) E3 ubiquitin ligase ligands exploiting constitutive hydroxyproline for benzylic amine protection†

**DOI:** 10.1039/d4ra01974a

**Published:** 2024-05-28

**Authors:** Diana M. Soto-Martínez, Garrett D. Clements, John E. Díaz, Joy Becher, Robert C. Reynolds, Christina Ochsenbauer, Timothy S. Snowden

**Affiliations:** a Department of Chemistry and Biochemistry, The University of Alabama 250 Hackberry Lane Tuscaloosa AL 35487 USA snowden@ua.edu; b Department of Medicine, Division of Hematology and Oncology, University of Alabama at Birmingham Birmingham AL 35294 USA; c Center for Convergent Bioscience and Medicine, The University of Alabama 720 2nd St. Tuscaloosa AL 35401 USA

## Abstract

The von Hippel-Lindau (VHL) protein serves as the substrate recognition subunit of the multi-subunit Cullin-2 RING E3 ubiquitin ligase (CRL2^VHL^), which regulates intracellular concentrations of hypoxia inducible factors (HIFs) through a ubiquitin proteasome system (UPS) cascade. Strategic recruitment of CRL2^VHL^ by bi- or trifunctional targeted protein degraders (*e.g.*, PROTACs®) offers the prospect of promoting aberrant polyubiquitination and ensuing proteasomal degradation of disease-related proteins. Non-peptidic, l-hydroxyproline-bearing VHL ligands such as VH032 (1) and its chiral benzylic amine analog Me-VH032 (2), are functional components of targeted protein degraders commonly employed for this purpose. Herein, we compare two approaches for the preparation of 1 and 2 primarily highlighting performance differences between Pd(OAc)_2_ and Pd-PEPPSI-IPr for the key C–H arylation of 4-methylthiazole. Results from this comparison prompted the development of a unified, five-step route for the preparation of either VH032 (1) or Me-VH032 (2) in multigram quantities, resulting in yields of 56% and 61% for 1 and 2, respectively. Application of *N*-Boc-l-4-hydroxyproline rather than *N-tert*-butoxycarbonyl to shield the benzylic amine during the coupling step enhances step economy. Additionally, we identified previously undisclosed minor byproducts generated during arylation steps along with observations from amine deprotection and amidation reaction steps that may prove helpful not only for the preparation of 1 and 2, but for other VHL recruiting ligands, as well.

## Abstract 2

Step economical routes for the multigram preparation of popular VHL E3 ligase recruiting ligands.

## Graphical abstract

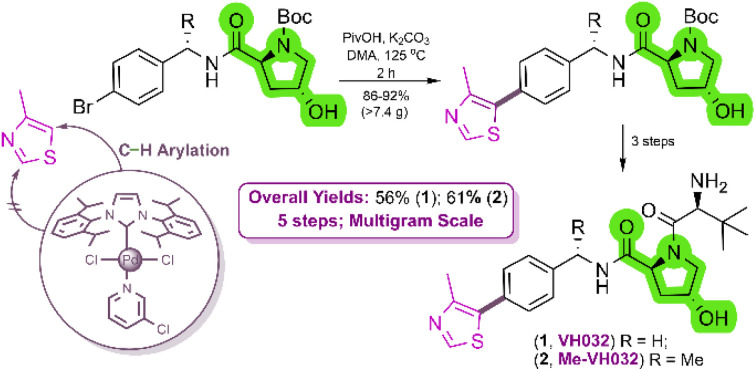

## Introduction

The von Hippel-Lindau (VHL) tumor suppressor protein has multiple cellular roles, including serving as the substrate recognition subunit of the Cullin-2 RING E3 ubiquitin ligase CRL2^VHL^ polyprotein. In this capacity, CRL2^VHL^ is commonly recruited by targeted protein degraders (*i.e.*, TPDs or PROTACs®^1^) and exploited for aberrant polyubiquitination of disease-related proteins, ideally resulting in the 26S proteasomal degradation of the target.^1–3^

Established VHL recruiting ligands and ligands targeting the substrate recognition receptor CRBN of the CRL4 E3 ubiquitin ligase predominate as functional segments of reported TPDs. CRBN ligands offer enhanced bioavailability,^4^ ease of preparation, and affordability; however, they are generally inferior to VHL ligands in terms of thermal and chemical stability^5–8^ and target selectivity.^9^ Because CRL2^VHL^ and CRL4^CRBN^ have different capacities to form stable ternary complexes with TPDs and the targeted proteins, and their recruiting ligands impart distinct physicochemical properties to potential degraders, it is beneficial to evaluate members of both ligand classes in the early stages of TPD discovery to increase the probability of hit identification.^10^

**Scheme 1:**
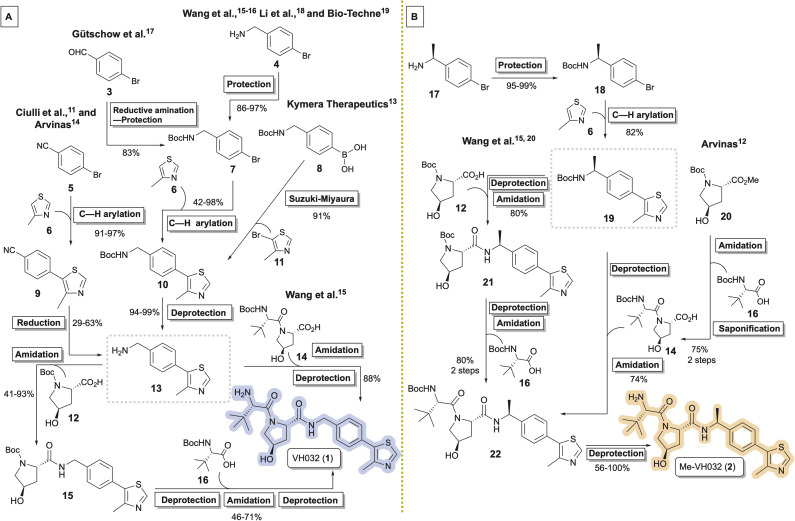
Synthetic routes (A) converging to key intermediate 13 en route to VH032 (1) and (B) diverging from 19 en route to Me-VH032 (2).

VH032 (1)^11^ and its chiral benzylic amine congener Me-VH032 (2)^12^ are among the most employed VHL ligands in TPDs. Reported syntheses of VH032 have relied upon C–H arylation of 4-methylthiazole 6 with Boc-protected benzylic amine 7, benzonitrile reduction of 9, or Suzuki–Miyaura cross coupling between 8 and 11 to access key intermediate 13.^11,13–17^ VH032 is subsequently assembled through sequential or convergent amidation and amine deprotection steps (Scheme 1A). Li and co-workers reported the highest yielding preparation of VH032, generating a 42.5 g batch in seven steps in 65% overall yield without chromatography.^18^ Researchers at Bio-Techne subsequently scaled up a comparable synthesis of 1 involving 7 to successfully prepare over 200 g batches.^19^ The routes originally reported by researchers at Arvinas and Yale and by the Ciulli group involving benzonitrile 5 remain popular, with multigram preparations of 1 completed in 25–65% overall yields in six steps from inexpensive commercial materials.^11,14^ Reported preparative methods for Me-VH032 (2) are more limited. Both researchers at Arvinas and the Wang group accessed 19*via* C–H arylation of 6 using Boc-protected chiral benzylic amine 18, followed by amine deprotection and standard amide coupling procedures to form 2 (Scheme 1B).^12,16,20^

Herein, we report a unified five-step strategy to prepare popular VHL recruiting ligand VH032 (1) and its higher affinity congener Me-VH032 (2) in multigram quantities. In addition, previously unreported observations related to minor byproduct formation during Pd-catalyzed 4-methylthiazole (6) arylation steps and during the sequential amine deprotection and amidation steps may prove helpful in the synthesis of these and other VHL recruiting ligands.

## Results and discussion

### Initial route

**Fig. 1:**
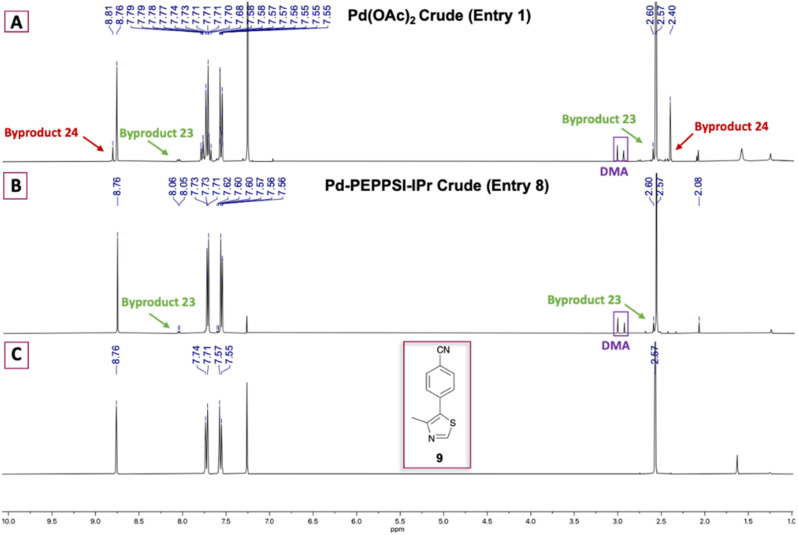
^1^H NMR comparison of crude 9 formed using (A) 0.1 mol% Pd(OAc)_2_ and (B) 0.5 mol% Pd-PEPPSI-IPr. (C) ^1^H NMR spectrum of purified 9.

**Fig. 2:**
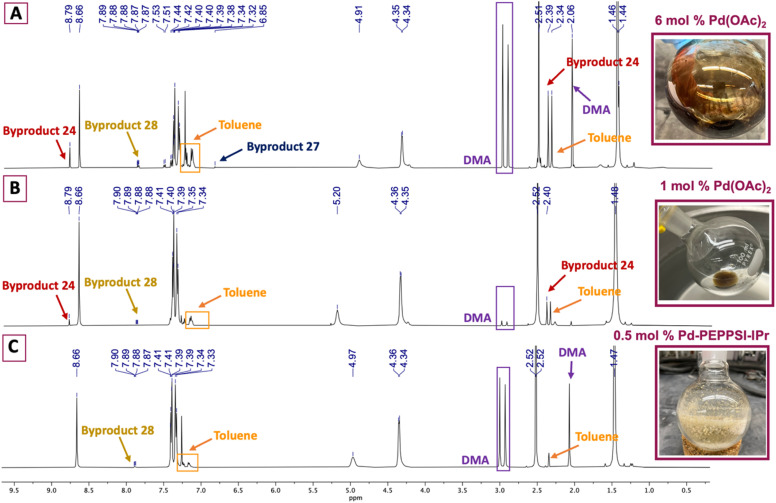
^1^H NMR and photographic comparison of crude 10 formed using: (A) 6 mol% Pd(OAc)_2_; (B) 1 mol% Pd(OAc)_2_; (C) 0.5 mol% Pd-PEPPSI-IPr. Residual reaction solvent, DMA, and azeotroping solvent, toluene, are indicated.

Initially, our goal was to improve the benzonitrile reduction step associated with popular synthetic routes for the multigram preparation of VH032 (1), since that step is yield limiting in published protocols involving synthetic intermediate 9 (Scheme 1A).^11,14^ We explored the preparation of requisite substrate 9 by initially comparing the C–H arylation conditions involving 2.0 equiv. 4-methylthiazole (6), 1.0 equiv. 4-bromobenzonitrile (5), 2.3 equiv. KOAc, and either 0.1 mol% or 3 mol% of Pd(OAc)_2_ in anhydrous DMA, as previously reported.^11,14^ The desired product 9 was obtained in excellent yields in both cases (Table 1, entries 1 and 2); however, both reactions generated two byproducts identified as 4,4′-(4-methylthiazole-2,5-diyl)dibenzonitrile (23) as a yellow solid (see page S11, Fig. 1S†) and 4,4′-dimethyl-5,5′-bithiazole (24) as a bright yellow solid (see page S12, Fig. 2S†), which were isolated in 5–8% combined yields (Fig. 1A). An attempt replacing KOAc with NaOAc under otherwise identical conditions to those in entry 2 offered comparable results (entry 3). Additional experiments using previously successful 3 mol% Pd(OAc)_2_ but only 1.3 equiv. of 6 at 100 °C or at 160 °C produced inferior results due to poor conversion of 9 in the first case and generation of multiple byproducts in the second (Table 1, entries 4 and 5).

**Table 1:** Comparative C–H arylation of 6 with 4-bromobenzonitrile (5) to afford 9

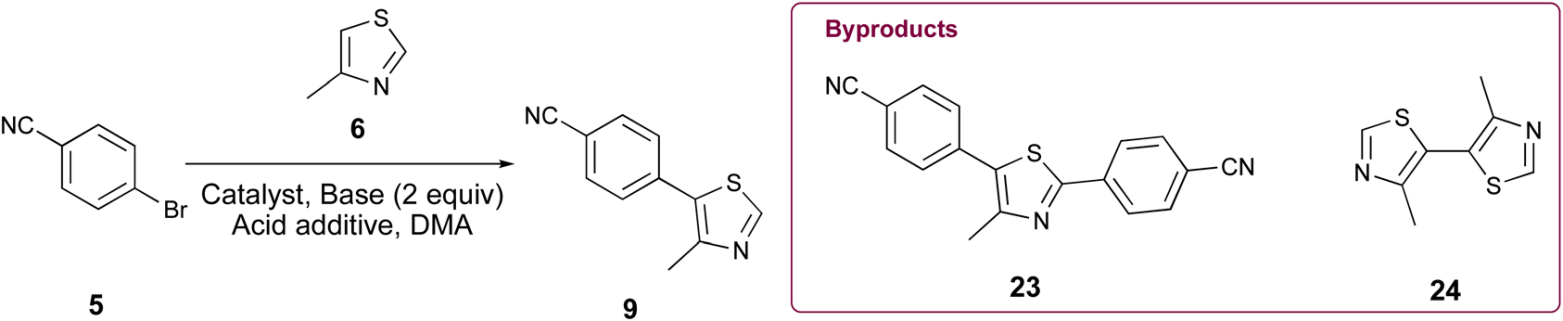									
Entry	Catalyst	Pd (mol%)	6 (equiv.)	Base	Additive	[ ] (M)	*T* (°C)	Time (h)	Yield (%)
1	Pd(OAc)_2_	0.1	2.0	KOAc		1.0	150	12	94
2	Pd(OAc)_2_	3	2.0	KOAc		0.5	150	5	88
3	Pd(OAc)_2_	3	2.0	NaOAc		0.5	150	3	86
4	Pd(OAc)_2_	3	1.3	NaOAc		0.5	160	4.5	47
5	Pd(OAc)_2_	3	1.3	NaOAc		0.5	100	5	30
6	Pd-PEPPSI-IPr	0.5	2.0	K_2_CO_3_	AcOH	0.25	125	3	70
7	Pd-PEPPSI-IPr	0.5	2.0	K_2_CO_3_	PivOH	0.25	100	3	86
8	Pd-PEPPSI-IPr	0.5	2.0	K_2_CO_3_	PivOH	0.25	125	2	93^a^
9	Pd-PEPPSI-IPr	0.5	2.0	K_2_CO_3_	PivOH	0.25	125	2	89^b^

a Scale = 250 mg of starting material 5 with purification by flash column chromatography.

b Scale = 5.15 g of starting material 5 with purification by trituration using ice and H_2_O.

Various palladium pyridine-enhanced precatalyst preparation stabilization and initiation (PEPPSI™) precatalysts have been demonstrated to afford C–H arylation products, including 9, in high yields.^21–23^ However, to our knowledge, these precatalysts have not been reported for the preparation of VHL ligands. Treatment of 5 and 6 with Organ's commercial Pd-PEPPSI-IPr^24,25^ at varied reaction temperatures and reaction times (Table 1, entries 6–9) furnished 9 in yields comparable or superior to those involving Pd(OAc)_2_ with the benefit of slightly reduced formation of bis-arylated 23 and no evidence of 24 (Fig. 1B). In addition, crude 9 produced by Pd-PEPPSI-IPr-catalyzed C–H arylation can be triturated using crushed ice/cold H_2_O to afford pure 9 as a pearl-colored powder in 89% yield demonstrated on a 5.15 g scale with no evidence of residual 23 or 24 (Table 1, entry 10; Fig. 1C)—a technique that failed when 9 was prepared using catalytic Pd(OAc)_2_ due to co-precipitation of minor byproduct 24. This trituration process may be appealing for the preparation of 9 in applications wherein flash chromatography is undesirable.

With benzonitrile 9 in hand, we compared the reductants LiAlH_4_, NaBH_4_ with NiCl_2_ ^26^ or CoCl_2_,^27^ and LiBH_4_ plus trimethylsilyl chloride^28^ to generate key benzylic amine intermediate 13 with an interest in increasing the isolated yield while also considering scalability. Unable to improve upon previously reported results involving LiAlH_4_,^14^ we explored Singaram's (^i^Bu)_2_AlBH_4_ reduction of 4-(4-methylthiazol-5-yl)benzonitrile (9).^29^ Our best results involved slight modifications to the originally reported conditions, largely to help manage B/Al-methanamine complex isolation from the associated solvogel produced upon quenching with methanol. Upon treating benzonitrile 9 for 2 hours with 1.1 equiv. of freshly prepared or briefly aged (^i^Bu)_2_AlBH_4_, followed by biphasic extraction from introduced aqueous Rochelle's salt, the B/Al-methanamine adduct was obtained as a tacky, yellow solid. Subsequently heating the metalloid-complexed amine at reflux in 6 M HCl for 3 hours reproducibly afforded the desired methanamine 13 in 69–74% yields, which was modestly superior to reductions of 9 using LiAlH_4_ and appreciably better than those involving the other evaluated reductants.

**Scheme 2:**
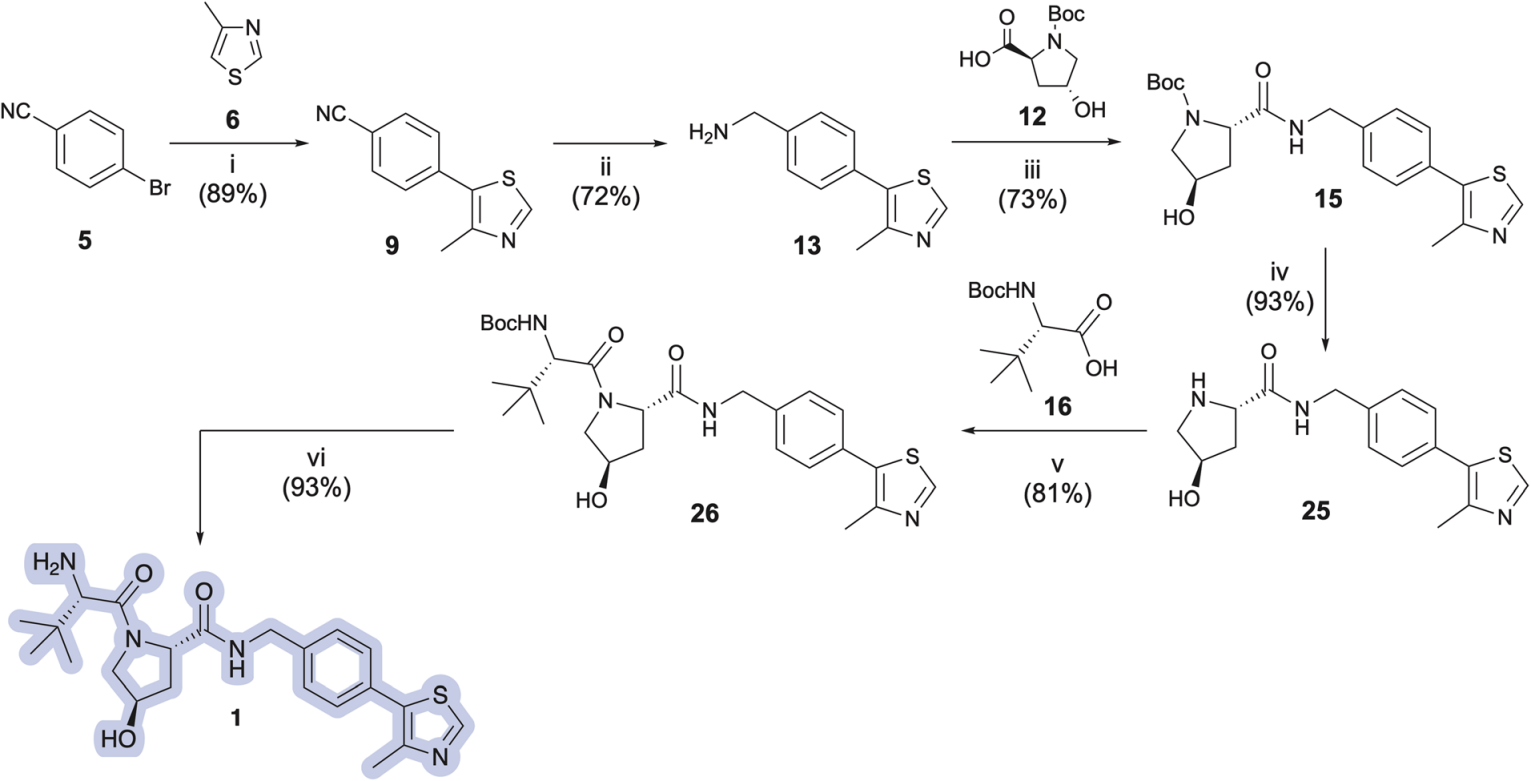
Initial route used to prepare VH032 (1). *Reagent and conditions*: (i) 0.5 mol% Pd-PEPPSI-IPr, K_2_CO_3_ (2 equiv.), PivOH (0.3 equiv.), DMA, 125 °C, 2 h; (ii) (a) (^i^Bu)_2_AlBH_4_ (1.1 equiv.), THF, 0 °C to r.t., 2 h; (b) 6 M HCl, reflux, 3 h; (iii) HATU (1.3 equiv.), DIPEA (3.5 equiv.), DMF, r.t., 19 h; (iv) (a) CH_2_Cl_2_ : TFA (1 : 1), 0 °C, 1 h; (b) NaOH solution until pH = 12.5–13; (v) HATU (1.3 equiv.), DIPEA (3.5 equiv.), DMF, r.t., 18 h; (vi) (a) CH_2_Cl_2_ : TFA (1 : 1), 0 °C, 1 h; (b) NaOH solution until pH = 12.5–13.

Key intermediate 13 was next treated with *N*-protected (2*S*,4*R*)-4-hydroxyproline (12, Boc-l-Hyp), hexafluorophosphate azabenzotriazole tetramethyl uronium (HATU), and *N*,*N*-diisopropylethylamine (DIPEA) to provide *N*-Boc-protected pyrrolidine carboxylate intermediate 15 in 73% yield (Scheme 2). Amine deprotection using a CH_2_Cl_2_ : TFA solution (1 : 1 v/v), followed by immediate freebasing and biphasic extraction from pH 12.5–13 aqueous medium afforded free amine 25 in 93% yield. The amidation strategy described for introduction of 12 to 13 was used to install Boc-l-*t*-leucine (16, Boc-l-Tle) onto 25, thereby furnishing penultimate VH032 product 26 in 81% yield. VH032 (1) was obtained using the amine deprotection/free basing strategy above in 93% yield, resulting in a 6-step preparation of 1 in 35% overall yield as free-based VH032 (1). The route offers comparable or superior step economy but lower overall yield compared to the best reported preparations of 1; however, identified improvements in select steps could be generally advantageous for the synthesis of other VHL ligands or unrelated target structures derived from 9 or 13. In addition, information gained from this initial approach inspired improvements for a more effective and expeditious preparative route to VH032 (1) and its analog Me-VH032 (2), *vide infra*.

Appealing procedures featuring 4-methylthiazolylation of *N*-Boc protected benzylic amine derivatives (*i.e.*, 7, 8, and 18, Scheme 1) as starting materials or early intermediates in the preparation of 1 and 2 have been reported.^11,12^ The advantage of these approaches for the preparation of 1 is obviation of the problematic benzonitrile reduction step required for transformation of 9 to 13 and employment of lower cost reactants than those required for Suzuki–Miyaura cross-coupling reactions.^13^ Given the comparative results between cat. Pd(OAc)_2_ and 0.5 mol% Pd-PEPPSI-IPr in the successful C–H arylation of 4-methylthiazole (6) using 5, we were interested to learn if the latter might offer some advantage in the C–H arylation of 6 using popular *tert*-butyl(4-bromobenzyl)carbamate 7. We initially evaluated conditions involving 6 mol% Pd(OAc)_2_, 2.3 equiv. methylthiazole (6), and 2.3 equiv. KOAc at 130 °C in anhydrous DMA (Table 2, entry 1),^18^ which furnished a viscous black oil following workup. The crude material was analyzed by ^1^H NMR spectroscopy, revealing signals for byproducts characterized as *tert*-butyl (4-(4-methylthiazol-5-yl)benzyl)carbamate (27), di-*tert*-butyl (((4-methylthiazole-2,5-diyl)bis(4,1-phenylene))bis(methylene))dicarbamate (28), and 4,4′-dimethyl-5,5′-bithiazole (24) along with desired product 10 (Fig. 2A). *N*-protected methylthiazole product 10 was isolated in 65% yield after flash chromatography and recrystallization using 1 : 4 CHCl_3_/hexanes – the latter required to remove residual, bright yellow 24 (see page S12, Fig. 2S†) that was not fully removed by chromatography. Other isolated materials included 8% of unreacted 7 and minor byproducts that could not be isolated and characterized. A recently reported method for the synthesis of 10 from 7 involving 1 mol% Pd(OAc)_2_, 2.0 equiv. methylthiazole (6), 2.0 equiv. KOAc in anhydrous DMA at 95 °C performed appreciably better, producing desired product 10 in 85% yield after workup and flash chromatography (Table 2, entry 2).^19^ The crude material was analyzed by ^1^H NMR spectroscopy (Fig. 2B), reflecting formation of 24 and 28 as minor byproducts with no evidence of 27 or other substantial impurities. Meanwhile, an attempt replacing Pd(OAc)_2_ with Pd-PEPPSI-IPr under the optimal conditions established for 4-methythiazolylation of benzonitrile 3 (Table 2, entry 3) produced superior results, with limited formation of byproducts 27 and 28 and no evidence of 24 in the ^1^H NMR spectrum of the crude product (Fig. 2C). Consequently, product 10 was isolated in 91% yield as an off-white solid after flash chromatography.

**Table 2:** Comparative C–H arylation of 6 using *tert*-butyl(4-bromobenzyl)carbamate (7)

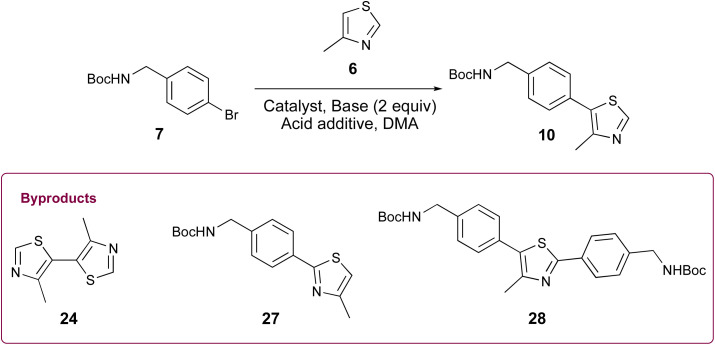									
Entry	Catalyst	Pd (mol%)	6 (equiv.)	Base	Additive	[ ] (M)	*T* (°C)	Time (h)	Yield^a^ (%)
1 ^18^	Pd(OAc)_2_	6	2.3	KOAc		0.4	130	4	65
2 ^19^	Pd(OAc)_2_	1	2.0	KOAc		0.6	95	18	85
3	Pd-PEPPSI-IPr	0.5	2.0	K_2_CO_3_	PivOH	0.25	130	2	91

a Isolated yield of 10 following purification *via* Combiflash.

### Route 2

Since the carbamate's role in 7 and 18 (Scheme 1) is to protect the Pd catalyst from amine coordination and associated side reactions that would complicate the C–H arylation step, we considered whether *N*-Boc-protected (2*S*,4*R*)-4-hydroxyproline (12, Boc-l-Hyp) might serve the same purpose with the hydroxyproline simultaneously acting as a constitutive moiety in the desired VHL ligand products, thereby eliminating an amine deprotection step. Researchers at GlaxoSmithKline reported this reaction in a 2014 patent using 2 mol% Pd(OAc)_2_ in *N*-methylpyrrolidinone (NMP) at 120 °C, obtaining 15 in 59% yield on an 8.0 g scale.^30^ No use of this approach or optimization is evidently reported beyond the one sentence reaction description in the patent. However, encouraged by the C–H arylation results highlighted in Tables 1 and 2, we compared the ability of Pd PEPPSI-IPr pre-catalyst to produce 15 from 29 relative to Pd(OAc)_2_ under various conditions, with the prospect of obviating the requirement for nitrile reduction or benzylic amine protection/deprotection steps en route to 15 or 21.

Initially, we conducted the amidation of commercial Boc-l-Hyp (12) with 4-bromobenzylamine (4) using HATU (1.2 equiv.) and DIPEA (3.0 equiv.) in anhydrous acetonitrile at room temperature for 13 h, which afforded desired product 29 (Table 3) in 92–98% yields after column chromatography. However, the subsequent 4-methylthiazole C–H arylation step with 29 underwent incomplete conversion, likely due to partial deactivation of the catalyst by a small amount of residual tetramethylguanidinium or tetramethylurea byproduct that was not completely removed from 29 through either biphasic extraction or subsequent flash chromatography. With the idea of avoiding formation of potentially irremovable guanidinium or urea impurities, we switched to amidation of 4 with 12 using 1.3 equivalents of *N*-(3-dimethylaminopropyl)-*N*′-ethylcarbodiimide hydrochloride (EDC·HCl), 1.3 equivalents of hydroxybenzotriazole (HOBt) hydrate, and 2.3 equivalents of DIPEA in dimethylformamide (DMF). Under these conditions, pure 29 was obtained in 45–50% yields along with 15–20% yields of a byproduct (33, see pages S8–S9†) resulting from esterification of the secondary alcohol in 29 with 12. Comparable results were obtained from multiple amidation attempts involving varied reaction times and temperatures. However, replacing DMF with the mixed solvent system CH_2_Cl_2_ : DMF (5 : 1 v/v) at −10 °C reported by Joullie and co-workers^31,32^ furnished 29 in 86% yield with no evidence of ester byproduct formation by ^1^H or ^13^C NMR spectroscopy in this or any subsequent amidation conducted on reaction scales ranging from 150 mg to >7 g of prepared 29.

**Table 3:** Investigation of the C–H arylation of 4-methylthiazole (6) with 29 to obtain 15

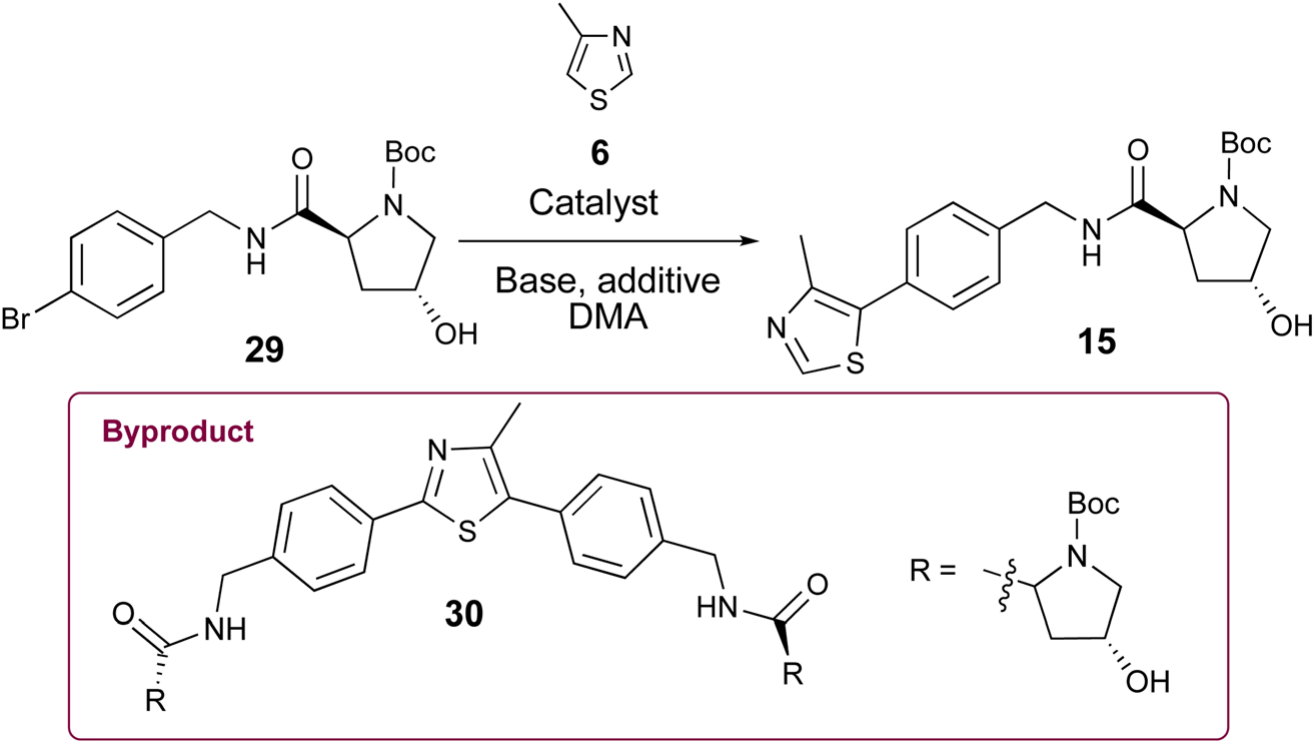								
Entry	Catalyst	Base	Additive	Pd (mol%)	[ ] (M)	*T* (^°^C)	Time (h)	Yield (%)
1	Pd(OAc)_2_	KOAc		2	0.4	120	18	58^a^
2	Pd(OAc)_2_	KOAc		0.1	1.0	150	1.5	53
3	Pd(OAc)_2_	KOAc		3	0.5	150	2	53
4	Pd(OAc)_2_	KOAc		1	0.8	130	4	75
5	Pd-PEPPSI-IPr	K_2_CO_3_	PivOH	0.25	0.25	125	19	21
6	Pd-PEPPSI-IPr	K_2_CO_3_	PivOH	0.5	0.25	100	21	37
7	Pd-PEPPSI-IPr	K_2_CO_3_	PivOH	0.5	0.25	125	2	85
8	Pd-PEPPSI-IPr	K_2_CO_3_	PivOH	0.5	0.25	125	2	88^b^
9	Pd-PEPPSI-IPr	K_2_CO_3_	PivOH	0.5	0.25	140	3	74
10	Pd-PEPPSI-IPr	K_2_CO_3_	PivOH	1	0.25	100	19	73
11	Pd-PEPPSI-IPr	K_2_CO_3_	PivOH	1	0.25	125	2.5	70
12	Pd(OAc)_2_	KOAc		0.5	0.25	125	1.5	80

a Reaction conducted in *N*-methylpyrrolidinone (NMP).

b Scale = 7.4 g of 29.

With ample 29 in hand, we explored its utility in the C–H arylation of 4-methylthiazole (6). Invariably, 4-methylthiazole coupling reactions involving catalytic Pd(OAc)_2_ proved inferior with 29 relative to those explored with 5 or reported by others with 7. The conditions reported by the GSK researchers worked as described in our hands, affording pure 15 in 58% isolated yield on a 250 mg scale (Table 3, entry 1). Treating 29 with 1.0 mol% Pd(OAc)_2_ and 2.0 equiv. KOAc in anhydrous DMA at 130 °C provided the best initial results for the conversion of 29 to 15 (Table 3, entry 4), offering marked improvement over reactions conducted at different temperatures and catalyst concentrations (*e.g.*, entries 2–3) in which byproducts including 24 and 30 were observed shortly after reaction commencement. 4-Methylthiazole coupling reactions with 29 involving 0.5% Pd-PEPPSI-IPr were generally more successful. Much like reaction attempts involving Pd(OAc)_2_ as summarized in Tables 1 and 2, we found the mol% of Pd-PEPPSI-IPr employed and reaction temperature significantly affected reaction outcomes in the generation of desired 15. Attempts at 100 °C or involving 0.25 mol% precatalyst proved sluggish with poor conversion (Table 3, entries 5 and 6), while those at 140 °C or involving 1 mol% Pd-PEPPSI-IPr showed high conversion but increased byproduct formation (Table 3, entries 9–11). The optimal conditions involved mixing 29 with pivalic acid (0.3 equiv.), K_2_CO_3_ (2 equiv.), and 0.5 mol% Pd-PEPPSI-IPr in DMA at 125 °C for 2 h (Table 3, entry 7). Under these conditions, yields of 15 invariably fall within the 85–88% range independent of the reaction scale evaluated (compare entry 7, conducted on a 250 mg scale with entry 8, conducted on a 7.4 g scale) or the batch produced. In addition, byproduct 30 formation was minimal, thereby facilitating rapid product purification relative to most reactions involving Pd(OAc)_2_. As a direct comparison of catalysts, we repeated the C–H arylation of 29 using Pd(OAc)_2_ in place of Pd-PEPPSI-IPr under the optimized conditions established with the latter and noted a slightly improved result relative to entry 4 (Table 3, entry 12), albeit still inferior to reactions completed using Pd-PEPPSI-IPr.

**Scheme 3:**
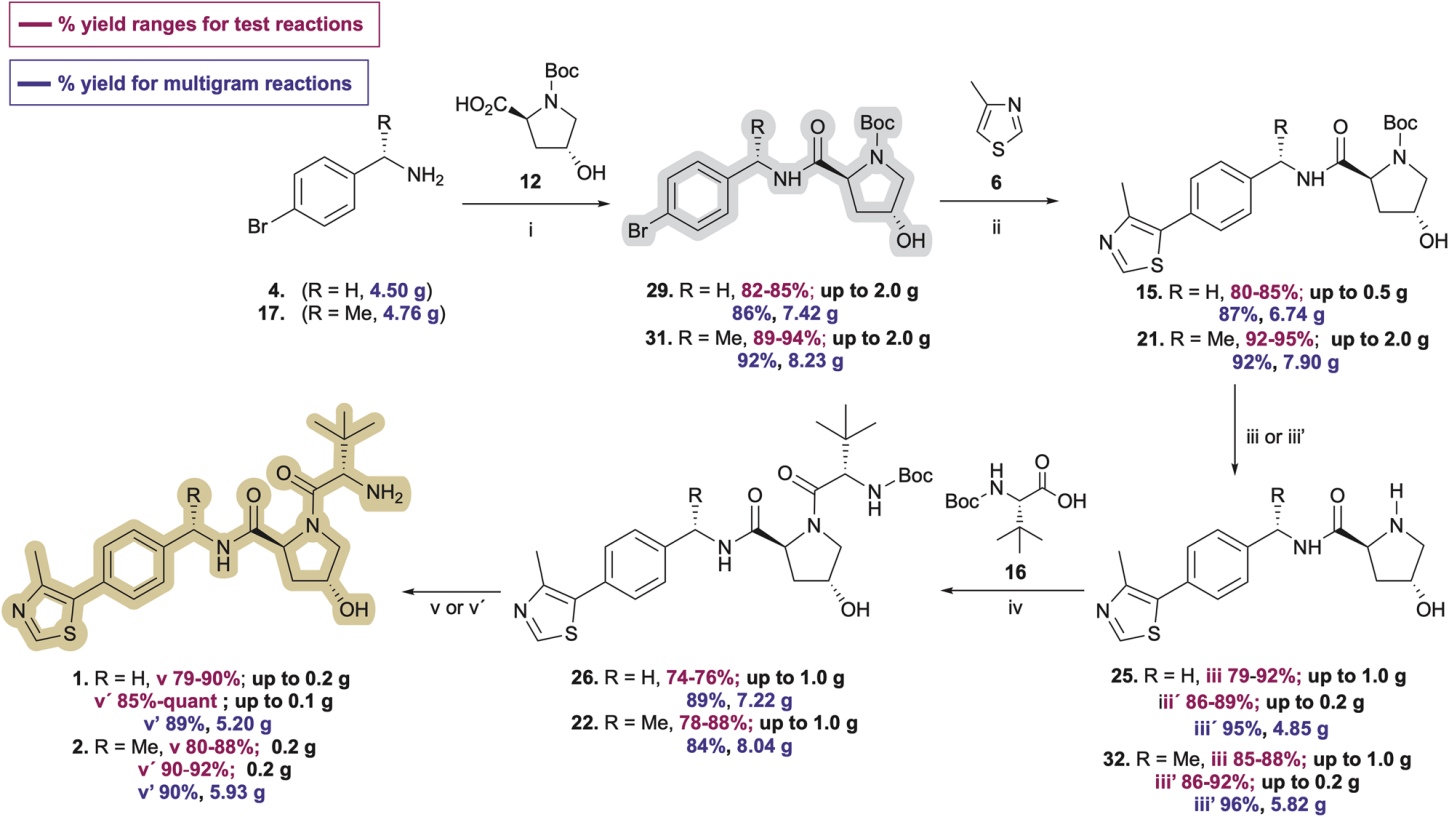
Optimized syntheses of VH032 (1) and Me-VH032 (2). Reagent and conditions: (i) EDC·HCl (1.3 equiv.), HOBt (1.3 equiv.), DIPEA (2.3 equiv.), CH_2_Cl_2_ : DMF (5 : 1), −10 °C to 0 °C, 1 h then 0 °C to r.t., 17 h; (ii) 0.5 mol% Pd-PEPPSI-IPr, K_2_CO_3_ (2 equiv.), PivOH (0.3 equiv.), DMA, 125 °C, 2.5 h; (iii) (a) CH_2_Cl_2_ : TFA (1 : 1), 4 °C, 1.5 h; (b) NaOH solution until pH = 12.5–13; (iii′) (a) 4 M HCl in MeOH, 4 °C, 2 h; (b) NaOH solution until pH = 12.5–13; (iv) EDC·HCl (1.3 equiv.), HOBt (1.3 equiv.), DIPEA (2.3 equiv.), CH_2_Cl_2_ : DMF (5 : 1), −10 °C to 0 °C, 1 h then 0 °C to r.t., 18 h; (v) (a) CH_2_Cl_2_ : TFA (1 : 1), 4 °C, 0.5–1 h; (b) NaOH solution until pH = 12.5–13; (v′) (a) 4 M HCl in MeOH, 4 °C, 2 h; (b) NaOH solution until pH = 12.5–13.

Pleased with the arylation results involving 6 and 29, we adapted the strategy to synthesize the key intermediate for the preparation of Me-VH032 (2). Commercial (*S*)-1-(4-bromophenyl)ethanamine (17) was amidated with Boc-l-Hyp (12) as described for 29, resulting in reproducibly high yields of 31 (averaging 90% in seven attempts) with no evidence of competing Boc-l-Hyp ester formation (Scheme 3). Treatment of resultant 31 with 6 (2.0 equiv.), pivalic acid (0.3 equiv.), K_2_CO_3_ (2 equiv.), and 0.5 mol% Pd-PEPPSI-IPr in DMA at 125 °C for 2 h cleanly furnished 21 in 95% yield, which is superior to reported C–H arylation of *N*-Boc-protected substrate 18 (Scheme 1).

Two common methods were evaluated to deprotect the prepared hydroxyproline intermediates 15 and 21. Initially, a TFA/CH_2_Cl_2_ solution (1 : 1 v/v, 0.1 M) was used to remove the Boc group and obtain the ammonium trifluoroacetate salt, which was subjected directly to amidation with *N*-Boc-l-*tert*-leucine (Boc-l-Tle) 16 using 1.3 equivalents of both EDC·HCl and HOBt monohydrate and 3.5 equivalents of DIPEA. Alternatively, a 4 M HCl solution in methanol was used (0.5 M), and the crude product was triturated using anhydrous MTBE and dried under vacuum to afford the desired product 25 as its presumed bis-hydrochloride salt (Table 4), although the isolated solvent-free product masses contradict the assumed product molecular weight. The ammonium salt was subsequently treated with Boc-l-Tle (16), 1.3 equivalents each of EDC·HCl and HOBt monohydrate, and 3.5 equivalents of DIPEA. Amidation yields involving ammonium trifluoroacetate or chloride salts from 25 and 32 with Boc-l-Tle varied widely in our hands, ranging from ∼35 to 75% in multiple attempts. Better amidation results were observed using ammonium chloride salts, possibly because the associated trituration step of the deprotected amines resulted in reduced residual chloride and HCl compared with residual trifluoroacetate and TFA from its corresponding simple evaporation from deprotected 15 and 21. Such counterions are rarely quantified in published preparations of 25 or 32, and the extent of both counterion inclusion and product hydration likely varies from batch to batch and by method and length of product storage.

**Table 4:** Comparative *N*-Boc removal from 15 and 21

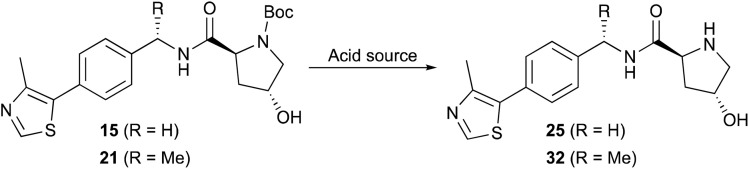						
Entry	R	Acid source	[ ] (M)	Time (h)	Yield (%) ammonium salt	Yield (%) free-based
1	H	HCl	0.75	6	89^a^	—
2	H	HCl	0.75	1	92^a^	90^b^
3	H	HCl	0.5	1.2	95^a^	89^b^
4	H	HCl	0.5	2	125^a^	95^c^
5	Me	HCl	0.5	1.5	102^a^	92^b^
6	Me	HCl	0.5	2	117^a^	96^d^
7	Me	HCl	0.3	1.2	92^a^	83^b^
8	H	TFA	0.1	1	—	92^b^
9	Me	TFA	0.1	1	—	88^b^
10	Me	TFA	0.25	1.1	—	75^b^

a % yield is calculated based on the mass of assumed bis-HCl salt, although the amount of residual HCl and water of hydration associated with the product was not determined.

b Scale: 250 mg of 15 or 21.

c Scale: 6.74 g of 15.

d Scale: 6.66 g of 21.

Dissatisfied with the capriciousness of our amide coupling attempts using ammonium salts, and with particular concern regarding possible material loss during larger scale preparations of 1 and 2, we compared amine deprotection procedures involving freebasing the isolated crude conjugate acids of 15 and 21 by liquid–liquid extraction. Free-basing involved dissolving the dry trifluoroacetate salts in a mixture of dichloromethane-deionized water (3 : 1 v/v) with constant stirring, followed by dropwise addition of concentrated aqueous NaOH solution to achieve pH = 12.5–13.0. Liquid–liquid extractions were conducted until product was absent from the DCM phase by TLC (typically, 8–11 small volume extractions). Free-based compounds 25 and 32, isolated upon treatment of 15 and 21 with TFA : CHCl_2_, were obtained in 92% and 88% yields, respectively (Table 4, entries 8 and 9; Scheme 3, iii). Similarly, triturated hydrochloride salts were free-based by dissolving the solid in a mixture of DCM–MeOH–dH_2_O (4 : 3 : 1 v/v)—with MeOH required to promote dissolution using a limited solvent volume—followed by dropwise addition of concentrated aqueous NaOH solution to achieve pH = 12.5–13.0. Liquid–liquid extraction as described above yielded free-based products 25 and 32 in 89% and 92% yields from 15 and 21, respectively (Table 4, entries 3 and 5; Scheme 3, iii′). No purification was necessary after either procedure.

After successfully producing free amines 25 and 32, the compounds were treated with *N*-Boc-*tert*-leucine (16) using 1.3 equivalents each of EDC·HCl and HOBt monohydrate and 2.3 equivalents of DIPEA in DCM : DMF (5 : 1 v/v) at −10 °C to afford 26 and 22 in 76% and 88% yields on up to 1 g preparations (Scheme 3, iv), respectively. *N*-Deprotection of the *N-tert*-butoxycarbamoyl *tert*-leucine in 26 and 22 using TFA in DCM, followed by solvent evaporation and biphasic extraction at pH 12.5–13.0 produced free-based target ligands 1 and 2 in 90% and 88% yields, respectively. Comparatively, treatment of 26 and 22 with 4 M HCl in MeOH followed by trituration of the hydrochloride salt of 1 with Et_2_O^33^ and that of 2 with MTBE prior to the freebasing workup afforded 1 and 2 in 89% and 92% yields, respectively (Scheme 3). As a result, the overall yields for the five-step synthesis of neutral VHL ligands 1 and 2 on ∼200 mg scales were 46% for VH032 (1) and 62% for Me-VH032 (2).

Encouraged by these findings, preparations of VH032 (1) and methyl-VH032 (2) were scaled starting with 5.15 g of Boc-l-Hyp (12) as the limiting reagent. The yields of each step from the multigram-scale preparations were consistent with those from the corresponding smaller scale reactions conducted during reaction and procedural optimizations—apart from the deprotections of 15 and 21 using HCl in MeOH and the amidation of 16 with 25 to afford 26, which proved more effective on a multigram scale (Scheme 3). Quantitative NMR analyses of both final free-based amine products indicated purities of 97% for 1 and 98% for 2 from their multigram scale preparations. In total, these unified approaches for the preparation of VH032 (1) and Me-VH032 (2) resulted in 56% and 61% overall yields, respectively—the highest yet reported for 2—thereby producing >5 g of product in each case in five steps from commercial materials.

## Conclusions

We compared approaches to prepare the popular VHL recruiting ligands VH032 (1) and Me-VH032 (2), thereby finding commercial Pd-PEPPSI-IPr pre-catalyst to be particularly effective for the requisite C–H arylation of 4-methylthiazole (6), even when the *N*-protected 4-hydroxyproline segment of 1 and 2, rather than a conventional amine protecting group, serves as the benzylic amine blocking moiety. Subsequent optimizations in amine deprotection and ensuing amidation steps produced multigram quantities of the high purity VHL ligands. The final route, which is common to formation of either 1 or 2, features unprecedented step economy (*i.e.*, five steps from inexpensive commercial materials) and comparable or improved overall yields of 1 and 2 relative to other scalable approaches. In addition, minor byproducts formed during arylations under various conditions are reported for the first time, and more extensive characterization of key intermediates is offered to assist those generating 1, 2 or other VHL ligands featuring (2*S*,4*R*)-4-hydroxy-*N*-(4-(4-methylthiazol-5-yl)benzyl)pyrrolidine-2-carboxamide (*i.e.*, 25 or 32) segments.

## Experimental

All reactants, reagents, and solvents were purchased from Sigma-Aldrich, Ambeed, or VWR suppliers. Reactions were monitored by thin-layer chromatography (TLC) on pre-coated 0.25 mm silica glass plates (60F254) purchased from Silicycle and visualized using UV light (254 nm or 365 nm), an I_2_ chamber, and/or either ninhydrin or KMnO_4_ stain with mild charring. Flash chromatography was performed using silica gel (60 Å, 230–400 mesh) from Silicycle pre-dried in a 150 °C oven for at least 24 h with a manual column or a Teledyne ISCO Combiflash *R*_f_ 200i. ^1^H and ^13^C NMR spectra were recorded on a Bruker NEO-500 spectrometer with a cryoprobe. All reported ^1^H and ^13^C chemical shifts (*δ*_H_, *δ*_C_) are referenced to the residual ^1^H signal of deuterated solvents (CDCl_3_: ^1^H = 7.26 ppm, ^13^C = 77.16 ppm; (CD_3_)_2_CO: ^1^H = 2.05 ppm, ^13^C = 29.84 ppm; CD_3_OD: ^1^H = 3.31 ppm, ^13^C = 49.00 ppm).^34^ Mass spectra were recorded using a Waters Xevo G2-XS QToF with ACUITU UPLC M-Class equipped with ESI and a high-performance orthogonal-acceleration Time of Flight (oaToF) mass analyzer (MS2). Melting points were determined with a MelTemp 1001D capillary melting point apparatus and were uncorrected. 1,2,4,5-Tetrachloro-3-nitrobenzene (99.85% pure) from Sigma Aldrich was used as the internal standard for quantitative NMR studies. Specific rotations were determined using an AUTOPOL IV automatic polarimeter. FTIR spectra were obtained with a JASCO FT/IR-4100 spectrometer.

### General procedure I: amidation of Boc-l-Hyp (12) and Boc-l-Tle (16) to form 22, 26, 29, 31

To a flame-dried round-bottom flask equipped with a magnetic stir bar and septum were added 12 or 16 (1.0 equiv.), EDC·HCl (1.3 equiv.), and HOBt monohydrate (1.3 equiv.). Amine (1.1 equiv.) dissolved in dry CH_2_Cl_2_ : DMF (5 : 1 v/v) was added to the reaction flask to create a 0.25 M solution of 12 or 16. The resulting white suspension was cooled to −10 °C with stirring for 5 minutes, then DIPEA (2.3 equiv.) was added dropwise. The reaction mixture was stirred at −10 °C for 1 h then the temperature was increased to 4 °C followed by gradual warming to room temperature until full conversion was evident by TLC (generally 15–20 h). The reaction was quenched by adding deionized water, and the aqueous phase was extracted successively with CH_2_Cl_2_ then EtOAc. The combined organic phases were washed with an aqueous solution of NaHCO_3_ to remove much of the remaining HOBt, then dried over anhydrous sodium sulfate, filtered, and concentrated by rotary evaporation followed by azeotropic distillation with toluene by rotary evaporation (55–60 °C water bath) to remove residual DMF. The crude product was purified by flash column chromatography using oven-dried silica and 4–5% MeOH in DCM as the eluent to afford the desired amide product 22, 26, 29, or 31.

### General procedure II: C–H arylation using 0.5 mol% Pd-PEPPSI-IPr to form 15 and 21

To a two-neck round-bottom flask equipped with a magnetic stir bar and condenser were added bromoaryl derivatives 29 or 31 (1.0 equiv.), anhydrous potassium carbonate (2.0 equiv.), Pd-PEPPSI-IPr (0.5 mol%), pivalic acid (0.3 equiv.), 4-methylthiazole 6 (2.0 equiv.) in anhydrous DMA (0.25 M). The charged flask was placed into an oil bath that had been pre-equilibrated to 125 °C, and the reaction was heated for 2 h at that temperature. The mixture was cooled to room temperature, quenched with deionized water (equal to the DMA volume used in the reaction), then the aqueous phase was extracted with EtOAc (5 × ∼5 mL mmol^−1^). The organic phase was dried over anhydrous sodium sulfate, filtered, and concentrated by rotary evaporation to remove EtOAc. Residual DMA was removed by azeotropic distillation by rotary evaporation between 55 and 60 °C using toluene. The crude product was purified by flash column chromatography using oven-dried silica and 5% MeOH in DCM to afford the desired product 15 or 21.

### General procedure III: amine deprotection to form 1, 2, 25, 32

The Boc-protected amine 15, 21, 22, or 26 (1.0 equiv.) was stirred in a solution of 4 M HCl in methanol (2 mL mmol^−1^) at 4 °C for 2 h in a one-neck round-bottom flask equipped with a magnetic stir bar. The volatile components were removed by rotary evaporation at 33 °C, and the resulting oil was dried overnight under vacuum. The hydrochloride salt was stirred in cold MTBE (for compounds 25, 32 and 2) or diethyl ether (for 1) for 1–2 h at 4 °C then the solid was collected by filtration and rinsed with cold MTBE or Et_2_O, as appropriate, to obtain an off-white amorphous solid. The solid was dissolved in a mixture of MeOH : DCM :vH_2_O (3 : 4:1 v/v, 8 mL mmol^−1^) at 4 °C, then a concentrated NaOH solution was slowly added with rapid mixing until the pH reached 12.5 to 13.0. The aqueous phase was extracted using small volumes of DCM until no product was evident in the extracting solvent by TLC (typically 8–11 times). The combined extracts were dried over anhydrous sodium sulfate, filtered, and concentrated by rotary evaporation at 33 °C to afford the desired product as analytically pure, free-based amine.

#### (2*S*,4*R*)-1-((*S*)-2-amino-3,3-dimethylbutanoyl)-4-hydroxy-*N*-(4-(4-methylthiazol-5-yl)benzyl)pyrolidine-2-carboxamide (1)

The title compound was prepared following General procedure III by treating *tert*-butyl-((*S*)-1-((2*S*,4*R*)-4-hydroxy-2-((4-(4-methylthiazol-5-yl)benzyl)carbamoyl)pyrolidin-1-yl)-3,3-dimethyl-1-oxobutan-2-yl)carbamate (26) (7.21 g, 13.6 mmol) with a 4 M HCl solution in methanol (28 mL). The crude product was stirred with diethyl ether (40 mL) at 0 °C for 2 h then vacuum filtered while rinsing with cold diethyl ether. The triturated solid was collected and dried overnight under vacuum affording 6.67 g of 1 as its beige hydrochloride salt. Free-based 1 was obtained as an off-white solid (5.20 g, 12.1 mmol, 89% yield) with a purity of 97% as determined by quantitative NMR analysis.

m.p.: 93–95 °C. *R*_f_: 0.10 (5% MeOH/CH_2_Cl_2_).

FT-IR (KBr, plate) *ν*_max_ (cm^−1^): 3360, 3298, 3072, 2954, 2869, 1668, 1624, 1553, 1439, 1416, 1221, 1199, 1080, 962, 848, 751.

^1^H NMR (500 MHz, CDCl_3,_ OH signal not evident in ^1^H NMR spectrum) *δ* (ppm): 8.65 (s, 1H, C*H* thiazole), 7.65 (t, *J* = 6.1 Hz, 1H, N*H*C = O), 7.31 (d, *J* = 8.5 Hz, 2H, Ar–*H*), 7.29 (d, *J* = 8.5 Hz, 2H, Ar–*H*), 4.71 (t, *J* = 8.1 Hz, 1H, O=C–C*H*–N Hyp), 4.45–4.41 (m, 2H, ((CH_2_)C*H*OH) Ar–C*H*_2_–N), 4.27 (dd, *J* = 15.2, 5.7 Hz, 1H, Ar–C*H*_2_–N), 3.71 (d, *J* = 11.3 Hz, 1H, C*H*_2_ Hyp), 3.58 (dd, *J* = 11.0, 3.8 Hz, 1H, C*H*_2_ Hyp), 3.29 (s, 1H, O=C–C*H*–N Boc-l-Tle), 2.79 (s br, 2H, N*H*_2_), 2.48 (s, 3H, C*H*_3_ thiazole), 2.29 (ddd, *J* = 13.3, 8.6, 4.4 Hz, 1H, C*H*_2_ Hyp), 2.12 (ddt, *J* = 13.3, 7.7, 1.8 Hz, 1H, C*H*_2_ Hyp), 0.91 (s, 9H, C(C*H*_3_)_3_).

^13^C NMR (126 MHz, CDCl_3_) *δ* (ppm): 173.90 (CONH Hyp), 171.70 (CONH Boc-l-Tle), 150.38 (CH thiazole), 148.49 (C thiazole), 138.46 (C–Ar), 131.71 and 130.83 (C–Ar, C thiazole), 129.48 and 127.92 (CH–Ar), 70.07 (CH Hyp), 60.54 (CH Boc-l-Tle), 58.97 (CH Hyp), 56.89 (CH_2_ Hyp), 43.05 (Ar–CH_2_–N), 37.16 (CH_2_ Hyp), 35.73 (C *t*-Bu Boc-l-Tle), 26.15 (CH_3_*t*-Bu Boc-l-Tle), 16.15 (CH_3_ thiazole).

HRMS QToF-ESI: calculated for C_22_H_31_N_4_O_3_S [M + H^+^] *m*/*z* 431.2117; found *m*/*z* 431.2117.[*α*]^D^_20_ = +34.3 (*c* = 0.035 in MeOH).

#### (2*S*,4*R*)-1-((*S*)-2-amino-3,3-dimethylbutanoyl)-4-hydroxy-*N*-((S)-1-(4-(4-methylthiazol-5-yl)phenyl)ethyl)pyrrolidine-2-carboxamide (2)

The title compound was prepared following General procedure III by treating *tert*-butyl-((*S*)-1-((2*S*,4*R*)-4-hydroxy-2-((4-(4-methylthiazol-5-yl)benzyl)carbamoyl)pyrolidin-1-yl)-3,3-dimethyl-1-oxobutan-2-yl)carbamate (22) (8.04 g, 14.8 mmol) with a 4 M HCl solution in methanol (30 mL). The crude product was stirred with MTBE (30 mL) at 0 °C for 2 h then vacuum filtered while rinsing with cold MTBE. The triturated solid was collected and dried overnight under vacuum affording 8.12 g of 2 as its white hydrochloride salt. Free-based 2 was obtained as an off white solid (5.93 g, 13.3 mmol, 90% yield) with a purity of 98% as determined by quantitative NMR analysis.

m.p.: 180–181 °C. *R*_f_: 0.10 (5% MeOH/CH_2_Cl_2_).

FT-IR (KBr plate), *ν*_max_ (cm^−1^): 3364, 3282, 3066, 2955, 2869, 1672, 1631, 1539, 1448, 1416, 1222, 1085, 968, 851, 835, 755.

^1^H NMR (500 MHz, CDCl_3_,; OH signal not evident in ^1^H NMR spectrum) *δ* (ppm): 8.60 (s, 1H, C*H* thiazole), 7.89 (d, *J* = 7.6 Hz, 1H, N*H*C=O), 7.31 (s, 4H, Ar–*H*), 5.00 (p, *J* = 7.1 Hz, 1H, Ar–C*H*–N), 4.70 (dd, *J* = 9.5, 7.6 Hz, 1H, O=C–C*H*–N Hyp), 4.38 (s br, 1H, (CH_2_)C*H*–OH), 3.65 (d, *J* = 11.0 Hz, 1H, CH_2_, C*H*_2_ Hyp), 3.54 (dd, *J* = 10.9, 4.1 Hz, 1H, C*H*_2_ Hyp), 3.29 (s, 1H, O=C–C*H*–N Boc-l-Tle), 2.44 (s, 3H, C*H*_3_ thiazole), 2.25 (ddd, *J* = 12.8, 7.8, 4.8 Hz, 1H, C*H*_2_ Hyp), 2.02 (m, 1H, C*H*_2_ Hyp), 1.42 (d, *J* = 7.0 Hz, 3H, C*H*_3_), 0.98 (s, 9H, C(C*H*_3_)_3_).

^13^C NMR (126 MHz, CDCl_3_), *δ* (ppm): 173.97 (CONH Hyp), 170.51 (CONH Boc-l-Tle), 150.39 (CH thiazole), 148.49 (C thiazole), 143.78 (C–Ar), 131.76 (C thiazole), 130.77 (C–Ar), 129.59 and 126.43 (CH–Ar), 69.97 (CH Hyp), 60.53 (CH Boc-l-Tle), 58.76 (CH Hyp), 56.77 (CH_2_ Hyp), 48.96 (Ar–CH–N), 36.74 (CH_2_ Hyp), 35.77 (C *t*-Bu Boc-l-Tle), 26.24 (CH_3_*t*-Bu Boc-l-Tle), 22.45 (CH_3_), 16.18 (CH_3_ thiazole).

HRMS QToF-ESI: calculated for C_23_H_33_N_4_O_3_S [M + H^+^] *m*/*z* 445.2273; found *m*/*z* 445.2276.[*α*]^D^_20_ = −128.0 (*c* = 0.025 in MeOH).

#### *tert*-Butyl (2*S*,4*R*)-4-hydroxy-2-((4-(4-methylthiazol-5-yl)benzyl)-carbamoyl)pyrrolidine-1-carboxylate (15)

The title compound was prepared following General procedure II using a solution of *tert*-butyl-(2*S*,4*R*)-2-((4-bromobenzyl)carbamoyl)-4-hydroxypyrrolidine-1-carboxylate (29) (7.34 g, 18.6 mmol), anhydrous potassium carbonate (5.16 g, 37.2 mmol), Pd-PEPPSI-IPr (0.065 g, 0.093 mmol), pivalic acid (0.58 g, 5.58 mmol), and 4-methylthiazole (6) (3.4 mL, 37.2 mmol) in 74 mL of anhydrous DMA. The product was purified by flash column chromatography using oven-dried silica with 5% MeOH in CH_2_Cl_2_ as the eluent affording 15 as a foamy, off-white solid (6.74 g, 16.2 mmol, 87% yield).

m.p.: 78–80 °C. *R*_f_: 0.45 (5% MeOH/CH_2_Cl_2_).

FT-IR (KBr plate) *ν*_max_ (cm^−1^): 3305, 3076, 2978, 2930, 1672, 1546, 1409, 1162, 858, 755.

^1^H NMR (500 MHz, CD_3_OD) *δ* (ppm): 8.874 and 8.865 (each s, 1H, C*H*, C*H* thiazole major and minor rotamer^17^), 7.43 and 7.42 (each s, 4H, the major and minor rotamer,^17^ Ar–*H*), 4.63–4.20 (m, 4H, Ar–C*H*_2_–N, O=C–C*H*–N, (CH_2_)C*H*–OH), 3.64–3.54 (m, 1H, C*H*_2_ Hyp), 3.54–3.43 (m, 1H, C*H*_2_ Hyp), 2.47 (s, 3H, C*H*_3_ thiazole), 2.34–2.20 (m, 1H, C*H*_2_ Hyp), 2.03 (ddd, *J* = 13.1, 8.6, 4.5 Hz, 1H, C*H*_2_ Hyp), 1.47 and 1.33 (s each, 9H, OC(C*H*_3_)_3,_ the major and minor rotamer^17^).

^13^C NMR (126 MHz, CD_3_OD, major rotamer^17^) *δ* (ppm): 175.56 (CONH), 156.17 (NCO), 152.92 (CH thiazole), 149.11 (C thiazole), 140.31 (C–Ar), 133.25 and 131.86 (C–Ar and C Thiazole), 130.51 and 129.69 (CH–Ar), 81.58 (C *N*-Boc), 70.05 (CH Hyp), 60.81 (CH Hyp), 56.00 (CH_2_ Hyp), 43.78 (Ar–CH_2_–N), 40.86 (CH_2_ Hyp), 28.54 (CH_3_*t*-Bu *N*-Boc), 15.79 (CH_3_ thiazole).

HRMS QToF-ESI: calculated for C_21_H_28_N_3_O_4_S [M + H^+^] *m*/*z* 418.1801; found *m*/*z* 418.1808.

#### *tert*-Butyl (2*S*,4*R*)-4-hydroxy-2-(((S)-1-(4-(4-methylthiazol-5-yl)phenyl)ethyl)carbamoyl) pyrrolidine-1-carboxylate (21)

The title compound was prepared following General procedure II using a solution of *tert*-butyl-(2*S*,4*R*)-2-(((*S*)-1-(4-bromophenyl)ethyl)carbamoyl)-4-hydroxypyrrolidine-1-carboxylate (31) (8.23 g, 19.9 mmol), anhydrous potassium carbonate (5.53 g, 39.8 mmol), Pd-PEPPSI-IPr (0.068 g, 0.99 mmol), pivalic acid (0.622 g, 5.97 mmol), and 4-methylthiazole (6) (3.60 mL, 39.8 mmol) in 80 mL of anhydrous DMA. The product was purified by flash column chromatography using oven-dried silica with 5% MeOH in CH_2_Cl_2_ as the eluent affording 21 as a foamy, off-white solid (7.90 g, 18.3 mmol, 92% yield).

m.p: 89–91 °C. *R*_f_: 0.40 (5% MeOH/CH_2_Cl_2_).

FT-IR (KBr plate) *ν*_max_ (cm^−1^): 3409, 3072, 2977, 2932, 1664, 1542, 1413, 1162, 1092, 858, 829, 751.

^1^H NMR (500 MHz, CD_3_OD; the major and minor rotamer; NH and OH signals not evident in ^1^H NMR spectrum) *δ* (ppm): 8.88 (s, 1H, C*H* thiazole), 7.48–7.40 (s, 4H, Ar–*H*), 5.20–5.01 (m, 1H, Ar–C*H*–N), 4.48–4.21 (m, 2H, O=C–C*H*–N, (CH_2_)C*H*–OH), 3.65–3.54 (m, 1H, C*H*_2_ Hyp), 3.54–3.42 (m, 1H, C*H*_2_ Hyp), 2.49 (s, 3H, C*H*_3_ thiazole), 2.29–2.19 (m, 1H, C*H*_2_ Hyp), 1.95 (ddd, *J* = 13.1, 8.7, 4.5 Hz, 1H, C*H*_2_ Hyp), 1.53 (d, *J* = 7.1 Hz, 3H, C*H*_3_), 1.49 and 1.43 (s each, 9H, OC(C*H*_3_)_3_, minor and major rotamer).

^13^C NMR (126 MHz, CD_3_OD, major rotamer) *δ* (ppm): 174.63 (CONH), 156.17 (NCO), 152.86 (CH thiazole), 149.06 (C thiazole), 145.50 (C–Ar), 133.28 and 131.61 (C–Ar or C thiazole), 130.50 and 127.73 (CH–Ar), 81.54 (C *N*-Boc), 70.04 (CH Hyp), 60.53 (CH Hyp), 56.06 (CH_2_ Hyp), 49.86 (Ar–CH–N), 40.70 (CH_2_ Hyp), 28.64 (CH_3_*t*-Bu *N*-Boc), 22.21 (CH_3_), 15.81 (CH_3_ thiazole).

HRMS QToF-ESI: calculated for C_22_H_30_N_3_O_4_S [M + H^+^] *m*/*z* 432.1957; found *m*/*z* 432.1956.

#### *tert*-Butyl ((*S*)-1-((2*S*,4*R*)-4-hydroxy-2-(((S)-1-(4-(4-methylthiazol-5-yl)phenyl)ethyl)carbamoyl)pyrolidin-1-yl)-3,3-dimethyl-1-oxobutan-2-yl)carbamate (22)

The title compound was prepared following General procedure I using (*S*)-*N*-Boc-2-amino-3,3-dimethylbutyric acid (16) (4.47 g, 19.3 mmol), EDC·HCl (3.55 g, 22.8 mmol), HOBt monohydrate (3.09 g, 22.8 mmol) and (2*S*,4*R*)-4-hydroxy-*N*-((*S*)-1-(4-(4-methylthiazol-5-yl)phenyl)ethyl)pyrrolidine-2-carboxamide (32) (5.83 g, 17.6 mmol) in CH_2_Cl_2_ (58.6 mL) and DMF (11.7 mL) followed by addition of DIPEA (6.9 mL, 40.4 mmol). The product was purified by flash column chromatography using 4% MeOH in CH_2_Cl_2_ as the eluent to afford 22 as an off-white solid (8.04 g, 14.8 mmol, 84% yield).

m.p.: 224–226 °C. *R*_f_: 0.35 (5% MeOH/CH_2_Cl_2_).

FT-IR (KBr plate) *ν*_max_ (cm^−1^): 3287, 3061, 2973, 2939, 2874, 1683, 1624, 1542, 1501, 1453, 1368, 1169, 837, 755.

^1^H NMR (500 MHz, CDCl_3,_ the major and minor rotamer; OH signal not evident in ^1^H NMR spectrum) *δ* (ppm): 8.67 (s, 1H, C*H* thiazole), 7.65 (d, *J* = 7.9 Hz, 1H, N*H*C = O), 7.38 (d, *J* = 8.3 Hz, 2H, Ar–*H*), 7.36 (d, *J* = 8.3 Hz, 2H, Ar–*H*), 5.28 (d, *J* = 9.2 Hz, 1H, N*H*), 5.07 (p, *J* = 7.1 Hz, 1H, Ar–C*H*–N), 4.72 (t, *J* = 7.9 Hz, 1H, O=C–C*H*–N Hyp), 4.48 (s br, 1H, ((CH_2_)C*H*–OH)), 4.22 (d, *J* = 9.3 Hz, 1H, O=C–C*H*–N Boc-l-Tle), 4.00 (d, *J* = 11.4 Hz, 1H, C*H*_2_ Hyp), 3.59 (dd, *J* = 11.4, 3.7 Hz, 1H, C*H*_2_ Hyp), 3.44 (s, 1H, O*H* Hyp), 2.51 (s, 3H, C*H*_3_ thiazole), 2.43 (ddd, *J* = 12.8, 7.8, 4.6 Hz, 1H, C*H*_2_ Hyp), 2.05 (dd, *J* = 13.5, 8.1 Hz, 1H, C*H*_2_ Hyp), 1.46 (d, *J* = 6.9 Hz, 3H, C*H*_2_ Hyp), 1.40 (s, 9H, major rotamer, OC(C*H*_3_)_3_), 1.01 (s, 9H, C(C*H*_3_)_3_ Boc-l-Tle).

^13^C NMR (126 MHz, CDCl_3_, the major rotamer) *δ* (ppm): 173.0 (CONH Hyp), 169.7 (CONH Boc-l-Tle), 156.6 (NCO), 150.6, (CH thiazole) 148.5 (C thiazole), 143.3 (C–Ar), 131.8 and 130.9 (C–Ar or C thiazole), 129.7 and 126.6 (CH–Ar), 80.6 (C *N*-Boc), 70.2 (CH Hyp), 59.1 (CH Boc-l-Tle), 58.4 (CH Hyp), 56.6 (CH_2_ Hyp), 49.0 (Ar–CH–N), 35.4 (CH_2_ Hyp), 35.0 (C *t*-Bu Boc-l-Tle) 28.4 (CH_3_*N*-Boc), 26.6 (CH_3_*t*-Bu Boc-l-Tle), 22.4 (CH_3_), 16.1 (CH_3_ thiazole).

HRMS QToF-ESI: calculated for C_28_H_41_N_4_O_5_S [M + H^+^] *m*/*z* 545.2798; found *m*/*z* 545.2794.

#### (2*S*,4*R*)-4-Hydroxy-*N*-(4-(4-methylthiazol-5-yl)benzyl)pyrrolidine-2-carboxamide (25)

The title compound was prepared following General procedure III by treating *tert*-butyl-(2*S*,4*R*)-4-hydroxy-2-((4-(4-methylthiazol-5yl)benzyl)carbamoyl)pyrrolidine-1-carboxylate (15) (6.74 g, 16.1 mmol) with a 4 M HCl solution in methanol (33 mL) at 4 °C for 2 h. The crude product was stirred with MTBE (50 mL) at 0 °C for 3 h then vacuum filtered while rinsing with cold MTBE. The triturated solid was collected and dried overnight under vacuum affording 6.71 g of 25 as its hydrochloride salt. The solid was dissolved in concentrated NaOH (dropwise addition at 4 °C) until pH = 12.5. Free-based 25 was obtained as a white foamy solid (4.85 g, 15.3 mmol, 95% yield).

m.p.: 108–110 °C. *R*_f_: 0.10 (5% MeOH/CH_2_Cl_2_).

FT-IR (KBr plate) *ν*_max_ (cm^−1^): 3312, 3077, 2925, 2862, 1653, 1520, 1417, 852, 807.

^1^H NMR (500 MHz, CD_3_OD) *δ* (ppm): 8.86 (s, 1H, C*H* thiazole), 7.42 (d, *J* = 8.0 Hz, 2H, Ar–*H*), 7.37 (d, *J* = 8.0 Hz, 2H, Ar–*H*), 4.43 (s, 2H, Ar–C*H*_2_–N), 4.36 (m, 1H, (CH_2_)C*H*OH), 3.94 (t, *J* = 8.2 Hz, 1H, O=C–C*H*–N), 3.01 (dd, *J* = 11.9, 3.9 Hz, 1H, C*H*_2_ Hyp), 2.90 (d, *J* = 12.0 Hz, 1H, C*H*_2_ Hyp), 2.46 (s, 3H, C*H*_3_), 2.18 (dd, *J* = 13.3, 8.0 Hz, 1H, C*H*_2_ Hyp), 1.86 (ddd, *J* = 13.5, 8.6, 5.0 Hz, 1H, C*H*_2_ Hyp).

^13^C NMR (126 MHz, CD_3_OD) *δ* (ppm): 177.13 (CONH), 152.83 (CH thiazole), 149.10 (C thiazole), 140.34 (C–Ar), 133.26 and 131.68 (C–Ar or C thiazole), 130.46 and 128.97 (CH–Ar), 73.52 (CH Hyp), 60.78 (CH Hyp), 56.05 (CH_2_ Hyp), 43.37 (Ar–CH_2_–N), 41.01 (CH_2_ Hyp), 15.82 (CH_3_ thiazole).

HRMS QToF-ESI: calculated for C_16_H_19_N_3_O_2_S [M + H^+^] *m*/*z* 318.1276; found *m*/*z* 318.1287.

#### *tert*-Butyl ((*S*)-1-((2*S*,4*R*)-4-hydroxy-2-((4-(4-methylthiazol-5-yl)benzyl)carbamoyl)pyrolidin-1-yl)-3,3-dimethyl-1-oxobutan-2-yl)carbamate (26)

The title compound was prepared following General procedure I using (*S*)–*N*-Boc-2-amino-3,3-dimethylbutyric acid (16) (3.96 g, 16.8 mmol), EDC·HCl (3.12 g, 19.9 mmol), HOBt monohydrate (2.74 g, 19.9 mmol) and (2*S*,4*R*)-hydroxy-*N*-(4-(4-methylthiazol-5-yl)benzyl)pyrrolidine-2-carboxamide (25) (4.85 g, 15.3 mmol) in CH_2_Cl_2_ (50 mL) and DMF (10 mL) followed by addition of DIPEA (6.0 mL, 35.2 mmol). The product was purified by flash column chromatography using a 4–5% MeOH in CH_2_Cl_2_ gradient to afford 26 as a foamy, off-white solid (7.22 g, 13.6 mmol, 89% yield).

m.p.: 161–163 °C. *R*_f_: 0.45 (5% MeOH/CH_2_Cl_2_).

FT-IR (KBr plate) *ν*_max_ (cm^−1^): 3445, 3423, 3312, 3078, 2970, 2873, 1686, 1631, 1551, 1504, 1440, 1368, 1231, 1166, 763.

^1^H NMR (500 MHz, CDCl_3_, the major and minor rotamer;^17^ OH signal not evident in ^1^H NMR spectrum) *δ* (ppm): 8.67 (s, 1H, C*H* thiazole), 7.48 (t, *J* = 6.0 Hz, 1H, N*H*C = O), 7.33 (d, *J* = 8.3 Hz, 2H, Ar–*H*), 7.30 (d, *J* = 8.3 Hz, 2H, Ar–*H*), 5.23 (d, *J* = 9.1 Hz, 1H, N*H*C = O), 4.72 (t, *J* = 7.8 Hz, 1H, O=C–C*H*–N Hyp), 4.53 (dd, *J* = 15.0, 6.5 Hz, 1H, Ar–C*H*_2_–N), 4.51 (m, s br, 1H, (CH_2_)C*H*–OH), 4.28 (dd, *J* = 15.0, 5.2 Hz, 1H, Ar–C*H*_2_–N), 4.17 (d, *J* = 9.2 Hz, 1H, O=C–C*H*–N Boc-l-Tle), 3.98 (d, *J* = 11.2 Hz, 1H, 3.5 Hz, 1H, C*H*_2_ Hyp), 3.61 (dd, *J* = 11.3, 3.8 Hz, 1H, 3.5 Hz, 1H, C*H*_2_ Hyp), 2.49 (s, 3H, C*H*_3_ thiazole overlapping signal of *H* from C*H*_2_), 2.47–2.44 (m, overlapping signal of CH_3_, 1H, C*H*_2_ Hyp), 2.11–2.04 (m, 1H, C*H*_2_ Hyp), 1.39 (s, 9H, major and minor rotamer,^17^ OC(C*H*_3_)_3_), 0.91 (s, 9H, major rotamer,^17^ C(C*H*_3_)_3_ Boc-l-Tle).

^13^C NMR (126 MHz, CDCl_3_, the major rotamer^17^) *δ* (ppm): 172.56 (CONH Hyp), 170.93 (CONH Boc-l-Tle), 156.636 (NCO), 150.48 (CH thiazole), 148.47 (C thiazole), 138.23 (C–Ar), 131.74 and 130.74 (C–Ar or C thiazole), 129.56 and 128.11 (CH–Ar), 80.39 (C *N*-Boc), 70.15 (CH Hyp), 58.97 (CH Boc-l-Tle), 58.59 (CH Hyp), 56.59 (CH_2_ Hyp), 43.28 (Ar–CH_2_–N), 36.11 (CH_2_ Hyp), 35.16 (C *t*-Bu Boc-l-Tle), 28.42 (CH_3_*N*-Boc), 26.41 (CH_3_*t*-Bu Boc-l-Tle), 16.10 (CH_3_ thiazole).

HRMS QToF-ESI: calculated for C_27_H_39_N_4_O_5_S [M + H^+^] *m*/*z* 531.2641; found *m*/*z* 531.2648.

#### *tert*-Butyl (2S*R*,4R)-2-((4-bromobenzyl)carbamoyl)-4-hydroxypyrrolidine-1-carboxylate (29)

The title compound was prepared following General procedure I using *trans-N*-(*tert*-butoxycarbonyl)-4-hydroxy-l-proline (12) (5.10 g, 21.6 mmol), EDC·HCl (4.39 g, 28.1 mmol), HOBt monohydrate (3.87 g, 28.1 mmol), and 4-bromobenzylamine (4) (3.0 mL, 23.8 mmol) in CH_2_Cl_2_ (72 mL) and DMF (14.4 mL) followed by addition of DIPEA (8.5 mL, 49.7 mmol). The product was purified by flash column chromatography using 4% MeOH in CH_2_Cl_2_ to afford 29 as an off-white solid (7.42 g, 21.62 mmol, 86% yield).

m.p.:127–129 °C. *R*_f_: 0.50 (5% MeOH/CH_2_Cl_2_).

FT-IR (KBr plate) *ν*_max_ (cm^−1^): 3313, 2975, 2927, 1713, 1672, 1540, 1417, 1368, 1221, 759.

^1^H NMR (500 MHz, CD_3_OD, NH and OH signals not evident in ^1^H NMR spectrum) *δ* (ppm): 7.46 and 7.44 (d, *J* = 8.3 Hz, 2H, the major and minor rotamer,^35^ Ar–*H*), 7.24 (d, *J* = 8.3 Hz, 2H, and Ar–*H*), 4.58–4.12 (m, 4H, Ar–C*H*_2_–N, O=C–C*H*–N, (CH_2_)C*H*–OH), 3.61–3.52 (m, 1H, C*H*_2_ Hyp), 3.52–3.43 (m, 1H, C*H*_2_ Hyp), 2.23 (m, 1H, C*H*_2_ Hyp), 2.00 (ddd, *J* = 13.1, 8.6, 4.5 Hz, 1H, C*H*_2_ Hyp), 1.47–1.33, (s each, 9H, OC(C*H*_3_)_3,_ minor and major rotamer^34^).

^13^C NMR (126 MHz, CD_3_OD, major rotamer^34^) *δ* (ppm): 175.53 (C=ONH), 156.14 (C=O), 139.29 (C–Ar), 132.60 and 130.99 (CH–Ar), 122.02 (C–Br), 81.58 (C *N*-Boc), 70.02 (CH Hyp), 60.74 (CH Hyp), 55.97 (CH_2_ Hyp), 43.48 (Ar–CH_2_–N), 40.80 (CH_2_ Hyp), 28.51 (CH_3_*t*-Bu *N*-Boc).

HRMS QToF-ESI: calculated for C_17_H_23_N_2_O_4_NaBr [M + Na^+^] *m*/*z* 421.0739; found *m*/*z* 421.0731.

#### *tert*-Butyl (2*S*,4*R*)-2-(((S)-1-(4-bromophenyl)ethyl)carbamoyl)-4-hydroxypyrrolidine-1-carboxylate (31)

The title compound was prepared following General procedure I using *trans-N*-(*tert*-butoxycarbonyl)-4-hydroxy-l-proline (12) (5.10 g, 21.6 mmol), EDC·HCl (4.41 g, 28.1 mmol), HOBt monohydrate (3.87 g, 28.1 mmol), and (*S*)-1-(4-bromophenyl)ethanamine (17) (3.4 mL, 23.8 mmol) in CH_2_Cl_2_ (79 mL) and DMF (16 mL) followed by addition of DIPEA (8.5 mL, 49.7 mmol). The product was purified by flash column chromatography using 4% MeOH in CH_2_Cl_2_ as the eluent to afford 31 as a foamy, off-white solid (8.23 g, 19.9 mmol, 92% yield).

m.p.: 153–155 °C. R_f_: 0.45 (5% MeOH/CH_2_Cl_2_).

FT-IR (KBr plate) *ν*_max_ (cm^−1^): 3309, 2977, 2935, 1740, 1660, 1548, 1414, 1366, 1228, 770.

^1^H NMR (500 MHz, CD_3_OD, the major and minor rotamer; NH and OH signals not evident in NMR spectra) *δ* (ppm): 7.48–7.44 (m, 2H, and Ar–*H*), 7.28 and 7.24 (d each, *J* = 8.3 Hz, 2H, major and minor rotamer, Ar–*H*), 4.98 (q, *J* = 7.0 Hz, 1H, Ar–C*H*–N), 4.39–4.27 (m, 2H, O=C–C*H*–N, (CH_2_)C*H*OH), 3.59–3.51 (m, 1H, C*H*_2_ Hyp), 3.48–3.36 (m, 1H, C*H*_2_ Hyp), 2.30–2.11 (m, 1H, C*H*_2_ Hyp), 1.89 (ddd, *J* = 13.1, 8.8, 4.5 Hz, 1H, C*H*_2_ Hyp), 1.45 (d, *J* = 7.1 Hz, 3H, C*H*_3_), 1.48–1.36 (s each, 9H, OC(C*H*_3_)_3,_ major and minor rotamer).

^13^C NMR (126 MHz, CD_3_OD, major rotamer) *δ* (ppm): 174.58 (CONH), 156.12 (NCO), 144.51 (C–Ar), 132.58 and 129.10 (CH–Ar), 121.68 (C–Ar), 81.53 (C *N*-Boc), 70.01 (CH Hyp), 60.43 (CH Hyp), 56.02 (CH_2_ Hyp), 49.61 (Ar–CH_2_–N), 40.62 (CH_2_ Hyp), 28.62 (CH_3_*t*-Bu *N*-Boc), 22.10 (CH_3_).

HRMS Q-tof-ESI: calculated for C_18_H_25_N_2_O_4_NaBr [M + Na^+^] *m*/*z* 435.0895; found *m*/*z* 435.0898.

#### (2S,4R)-4-hydroxy-N-((S)-1-(4-(4-methylthiazol-5-yl)phenyl)ethyl)pyrrolidine-2-carboxamide (32)

The title compound was prepared following General procedure III by treating *tert*-butyl-(2*S*,4*R*)-4-hydroxy-2-(((*S*)-1-(4-(4-methylthiazol-5-yl)phenyl)ethyl)carbamoyl)pyrrolidine-1-carboxylate (21) (7.90 g, 18.3 mmol) with a 4 M HCl solution in methanol (37 mL) at 4 °C for 2 h. The crude product was stirred with MTBE (55 mL) at 0 °C for 2 h then vacuum filtered while rinsing with cold MTBE. The triturated solid was collected and dried overnight under vacuum affording 7.29 g of white 32 as its hydrochloride salt. The solid was dissolved in concentrated NaOH (dropwise addition at 4 °C) until pH = 12.5–13. Free-based 32 was obtained as a light yellow foamy solid (5.83 g, 17.6 mmol, 96% yield).

m.p.: 112–113 °C. *R*_f_: 0.10 (5% MeOH/CH_2_Cl_2_).

FT-IR (KBr plate) *ν*_max_ (cm^−1^): 3299, 3058, 2980, 2928, 1642, 1542, 961, 840, 763, 737.

^1^H NMR (500 MHz, CD_3_OD, NH and OH signals not evident in NMR spectra) *δ* (ppm): 8.86 (s, 1H, C*H* thiazole), 7.43 (m, 4H, Ar–*H*), 5.04 (q, *J* = 7.0 Hz, 1H, Ar–C*H*–N), 4.37–4.35 (m, 1H, (CH_2_)C*H*–OH), 3.91 (t, *J* = 8.3 Hz, 1H, O=C–C*H*–N), 3.06 (dd, *J* = 12.0, 4.2 Hz, 1H, C*H*_2_ Hyp), 2.89 (dt, *J* = 11.9, 1.8 Hz, 1H, C*H*_2_ Hyp), 2.47 (s, 3H, C*H*_3_ thiazole), 2.15 (ddt, *J* = 13.4, 7.8, 1.8 Hz, 1H, C*H*_2_ Hyp), 1.82 (ddd, *J* = 13.7, 8.7, 5.1 Hz, 1H, C*H*_2_ Hyp), 1.49 (d, *J* = 7.1 Hz, 3H, C*H*_3_).

^13^C NMR (126 MHz, CD_3_OD): *δ* (ppm) 174.38 (CONH), 151.48 (CH thiazole), 147.72 (C thiazole), 143.98 (C–Ar), 131.91 and 130.24 (C–Ar C thiazole), 129.14 and 126.27 (CH–Ar), 72.08 (CH Hyp), 59.27 (CH Hyp), 54.64 (CH_2_ Hyp), 48.33 (Ar–CH–N), 39.61 (CH_2_ Hyp), 21.00 (CH_3_), 14.46 (CH_3_ thiazole).

HRMS QToF-ESI: calculated for C_17_H_22_N_3_O_2_S [M + H^+^] *m*/*z* 332.1433; found *m*/*z* 332.1423.

##### Quantitative NMR product purity analysis

Compounds 1 and 2 were dissolved in anhydrous CDCl_3_ to make 0.041 M solutions. The quantitative NMR standard 1,2,4,5-tetrachloro-3-nitrobenzene (99.85% pure) was used as the internal standard in a ratio of 1 : 1 (m/m). 500 MHz ^1^H NMR spectra were obtained for each mixture with a relaxation delay of 16 s at 298 K for 1 and 313 K for 2. The purity of each compound was calculated by applying the following equation: 
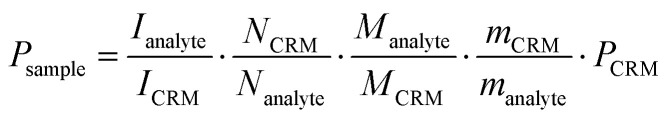
, where *P*_Sample_ = purity of the sample as mass fraction; *P*_CRM_ = purity of the Certificated Reference Material (CMR) as mass fraction; *I*_Analyte_ = integration of the analyte signal; *I*_CRM_ = integration of the CRM signal; *N*_Analyte_ = number of analyte protons; *N*_CRM_ = number of CRM protons; *M*_Analyte_ = molecular weight of the analyte; *M*_CRM_ = molecular weight of the CRM; *m*_Sample_ = mass of sample analysed; *m*_CRM_ = mass of CRM analysed.

## Author contributions

DMSM, GDC, JED, and JB conducted the synthesis experiments and completed compound characterization. DMSM and JED conducted quantitative NMR studies and authored the ESI.† DMSM and TSS wrote the original manuscript. JED and GDC reviewed and edited the manuscript. TSS conceptualized and supervised all studies. TSS, RCR, and CO acquired financial support. All authors have given approval of the final version of the manuscript.

## Conflicts of interest

The authors declare no conflict of interest.

## Supplementary Material

RA-014-D4RA01974A-s001
