# Supplementary material for: Preparation of von Hippel-Lindau (VHL) E3 ubiquitin ligase ligands exploiting constitutive hydroxyproline for benzylic amine protection
Source: RSC Adv. 2024 May 28;14(24):17077–90. doi: 10.1039/d4ra01974a (PMC11130640; doi:10.1039/d4ra01974a)
Supplement: RA-014-D4RA01974A-s001 [file RA-014-D4RA01974A-s001.pdf]

Electronic Supplementary Material (ESI) for RSC Advances.
This journal is © The Royal Society of Chemistry 2024

Supporting Information

Preparations of Von Hippel-Lindau (VHL) E3 Ubiquitin Ligase Ligands Exploiting Constitutive Hydroxyproline for Benzylic Amine Protection

Diana M. Soto-Martínez,[a] Garrett D. Clements,[a] John Edwar Díaz,[a] Joy Becher,[a] Robert C. Reynolds,[b] Christina Ochsenbauer,[b] and Timothy S. Snowden,*[a,c]

[a] Department of Chemistry and Biochemistry, The University of Alabama, 250 Hackberry Lane, Tuscaloosa, AL 35487, USA

[b] Department of Medicine, Division of Hematology and Oncology, University of Alabama at Birmingham, Birmingham, AL 35294, USA

[c] Center for Convergent Bioscience and Medicine, The University of Alabama, 720 2nd St., Tuscaloosa, AL 35401, USA

Email: snowden@ua.edu

Table of Contents

1. General Information …………………………………………………………………… S2
2. Route 1 and Individual Preparations of **9**, **10**, **13**, **15**, and **26**... S2
3. Characterization Data of Identified Reaction Byproducts …………..…………….......... S6
4. References ……………………………………………………………………………... S9
5. Appearance of **9**, **10** and Isolated Byproducts **23**, **24**, **27** and……………………………. S11
6. ^{1}H, ^{13}C, and 2D NMR spectra………………………………………………………...... S13
7. Quantitative NMR spectra...……………………………………………………….…..... S75

1. General Information

All reactants, reagents, and solvents were purchased from Sigma Aldrich, Ambeed, or VWR suppliers. Reactions were monitored by thin-layer chromatography (TLC) on pre-coated 0.25 mm silica gel glass plates (60F254) purchased from Silicycle and visualized using UV light (254 nm or 365 nm), an I_2 chamber, and/or either ninhydrin or $KMnO_4$ stain with mild charring. Flash chromatography was performed using silica gel (60 Å, 230 – 400 mesh) from Silicycle pre-dried in a 150°C oven for at least 24 h with a manual column or a Teledyne ISCO Combiflash R_f 200i. ^{1}H and ^{13}C NMR spectra were recorded on a Bruker NEO-500 spectrometer with a cryoprobe. All reported ^{1}H and ^{13}C chemical shifts (δH, δC) are referenced to the residual ^{1}H signal of deuterated solvents ($CDCl_3$: ^{1}H = 7.26 ppm, ^{13}C = 77.16 ppm; $(CD_3)_2CO$: ^{1}H = 2.05 ppm, ^{13}C = 29.84 ppm; CD_3OD: ^{1}H = 3.31 ppm, ^{13}C = 49.00 ppm).[1] Mass spectra were recorded using a Waters Xevo G2-XS QToF with ACUITU UPLC M-Class equipped with ESI and a high-performance orthogonal-acceleration Time of Flight (oaTOF) mass analyzer (MS2). Melting points were determined with a MelTemp 1001D capillary melting point apparatus and were uncorrected. 1,2,4,5-tetrachloro-3-nitrobenzene (99.85% pure) from Sigma Aldrich was used as the internal standard for quantitative NMR studies. Specific rotations were determined using an AUTOPOL IV automatic polarimeter. FTIR spectra were obtained with a JASCO FT/IR-4100 spectrometer.

.

2. Route 1 and Individual Preparations of 9, 10, 13, 15, and 26

4-(4-Methylthiazol-5-yl)benzonitrile (9)

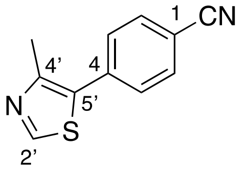

To a 500 mL flame-dried round-bottom flask equipped with a magnetic stir bar, rubber septum, and condenser were added 4-bromobenzonitrile (**5**) (5.16 g, 27.5 mmol), 4-methylthiazole (**6**) (5.1 mL, 54.9 mmol), Pd-PEPPSI-IPr (95.6 mg, 0.137 mmol), K_2CO_3 (7.61 g, 54.9 mmol), pivalic acid (0.841 g, 8.24 mmol), and 100 mL of anhydrous DMA. The reaction was heated at 125 ^{o}C for 2 h until full conversion was evident by TLC (30% EtOAc/Hexanes). The system was cooled to room temperature then the mixture was poured onto crushed ice and refrigerated overnight. The precipitate was filtered and repeatedly rinsed with cold dH_2O then dried under vacuum to afford the desired product **9** as a white solid (4.89 g, 24.4 mmol, 89% yield). Spectral data matched that reported previously.[2, 3]

m.p: 80-83° C. **R_f:** 0.30 (30% EtOAc/Hexanes)

FT-IR (KBr plate) ν_{max} (cm^{-1}): 3080, 3053, 2955, 2921, 2226, 1604, 1536, 1450, 1413, 852, 833.

^{1}H NMR (500 MHz, $CDCl_3$) δ (ppm): 8.76 (s, 1H, C**H** thiazole), 7.73 (d, *J* = 8.4 Hz, 2H, Ar-**H**), 7.57 (d, *J* = 8.4 Hz, 2H, Ar-**H**), 2.57 (s, 3H, C**H**$_3$).

^{13}C NMR (126 MHz, $CDCl_3$) δ (ppm): 151.71(**C-2'** thiazole), 150.20 (**C-4'**), 137. 02 (**C-4**), 132.65 (**C**-Ar), 130.23 (**C-5'** thiazole), 129.86,(**C**-Ar) 118.61 (**C**N), 111.66 (**C-1**), 16.50 (CH_3).

HRMS QToF-ESI: calculated for $C_{11}H_8N_2S$ [M$^+$] *m/z*: 200.0408; found *m/z*: 200.0404.

tert-Butyl (4-(4-methylthiazol-5-yl)benzyl)carbamate (10)

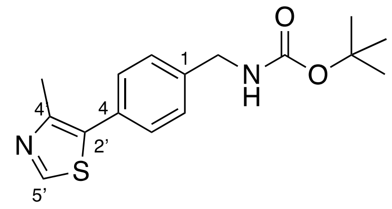

To a 25 mL flame-dried round-bottom flask equipped with a magnetic stir bar and rubber septum were added *tert*-butyl(4-bromobenzyl)carbamate (**7**) (1.00 g, 3.49 mmol), Pd-PEPPSI-IPr (0.012 g, 0.017 mmol), anhydrous potassium carbonate (0.967 g, 6.99 mmol), pivalic acid (0.108 g, 1.04 mmol), 4-methylthiazole (**6**) (0.64 mL, 6.99 mmol), and 14 mL anhydrous DMA. The reaction was heated at 130 ^{o}C for 2 h until full conversion was evident by TLC (10% EtOAc/Hexanes). The mixture was cooled to room temperature, quenched with deionized water (14 mL) and extracted using EtOAc until no product was evident in the extracting solvent by TLC (4 x 7 mL). The organic phase was dried over anhydrous sodium sulfate, filtered, and concentrated, followed by azeotropic removal of residual DMA with toluene by rotary evaporation at 55-60 ^{o}C. The crude was purified by flash column chromatography automatized (Combiflash, 12 g pre-packed flash column, 12 min) and a 10-20% EtOAc/hexanes gradient to afford **10** as a white solid (0.967 g, 3.18 mmol, 91% yield). Spectral data matched that reported previously.[4-6]

m.p.: 123-124 ^{o}C. **R$_f$**: 0.40 (10% EtOAc in Hexanes)

^{1}H NMR (500 MHz, $CDCl_3$) δ (ppm): 8.63 (s, 1H, C**H** thiazole), 7.37 (d, *J* = 8.1 Hz, 2H, Ar-**H**), 7.31 (d, *J* = 8.1 Hz, 2H, Ar-**H**), 5.14 (br s, 1H, N**H**-Boc), 4.31 (d, *J* = 5.5 Hz, 2H, Ph-C**H**$_2$-NHBoc), 2.49 (s, 3H, C**H**$_3$ thiazole), 1.45 (s, 9H, CO_2-C(C**H**$_3$)$_3$).

^{13}C NMR (126 MHz, $CDCl_3$) δ (ppm): 155.87 (N**C**O), 150.14 (**C**H thiazole), 148.37 (**C**-4 thiazole), 138.86 (**C**1-Ar), 131.48(**C**-5 thiazole), 130.77 (**C**-4 Ar), 129.35 and 127.60 (**C**-2 and **C**-3 Ar), 79.46 (O**C**(CH_3)$_3$), 44.13 (Ar-**C**H$_2$-N), 28.31 (OC(**C**H$_3$)$_3$), 15.96 (**C**H$_3$).

(4-(4-Methylthiazol-5-yl)phenyl)methanamine (13)

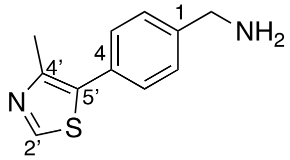

To a 25 mL flame-dried round-bottom flask equipped with a stir bar and rubber septum were added 4-(4-methylthiazol-5-yl)benzonitrile (**9**) (0.20 g, 0.99 mmol), and 1.8 mL of anhydrous THF under nitrogen atmosphere. Freshly prepared (iBu)$_2$AlBH$_4$[7] (1.2 mL, 1.01 mmol, 0.91 M in dichloromethane) was added dropwise at room temperature, and the reaction mixture was stirred for 3 h until full conversion was evident by TLC (5% NH_3-saturated MeOH/CH_2Cl_2). The reaction mixture was cooled to -5 to -10 °C (crushed ice/acetone bath) then quenched with methanol (5 mL) and stirred for 10-15 min. The resulting solvogel was dissolved by adding 5 mL of concentrated aqueous Rochelle's salt solution and stirring vigorously for 2 h. Following this, 3 mL of EtOAc were added and stirred for an additional hour. The aqueous phase was extracted using EtOAc until no product was evident in the extracting solvent by TLC (5 x 3 mL). The combined organic phase was dried over anhydrous sodium sulfate, filtered, and concentrated by rotary evaporation at 35 °C. Next, a 6 M HCl_{aq} solution (5 mL) was added, and the mixture was heated at 70 ^{o}C for 3 h then cooled to room temperature. The solution pH was carefully adjusted to pH =12 using 6 M $NaOH_{aq}$ solution then extracted using 3 mL portions of CH_2Cl_2 until no product was evident in the extracting solvent (i.e., typically 10 times). The resulting organic phase was dried over anhydrous sodium sulfate, filtered, and concentrated

by rotary evaporation at 32 °C. The product was purified by flash column chromatography using a 3-5% ammonia saturated MeOH in CH_2Cl_2 gradient affording **13** as a yellow oil (0.146 g, 0.717 mmol, 72% yield). Spectral data matched that reported previously.[7]

R$_f$: 0.10 (5% NH_{3sat} MeOH in CH_2Cl_2)

FT-IR (KBr plate) ν_{max} (cm^{-1}): 3364, 3294, 3068, 3024, 2957, 2921, 2856, 1606, 1535, 1446, 1413, 1376, 848, 818.

^{1}H NMR (500 MHz, $CDCl_3$) δ (ppm): 8.67 (s, 1H, C**H** thiazole), 7.42 (d, *J* = 8.4 Hz, 2H, Ar-**H**), 7.38 (d, *J* = 8.4 Hz, 2H, Ar-**H**), 3.92 (s, 2H, C**H**$_2$), 2.54 (s, 3H, C**H**$_3$).

^{13}C NMR (126 MHz, $CDCl_3$) δ (ppm): 150.28 (**C-2'** thiazole), 148.51 (**C-4'** thiazole), 143.13 (**C-1**), 131.84 (**C-5'** thiazole), 130.53 (**C-4**), 129.56 (**C**-Ar), 127.56 (**C**-Ar), 46.20 (**C**H_2), 16.18 (**C**H_3).

HRMS QToF-ESI: calculated for $C_{11}H_{13}N_2S$ [M+H$^+$] *m/z*: 205. 0799; found *m/z*: 205.0804.

tert-Butyl (2*S*,4*R*)-4-hydroxy-2-((4-(4-methylthiazol-5-yl)benzyl)carbamoyl)pyrrolidine-1-carboxylate (15)

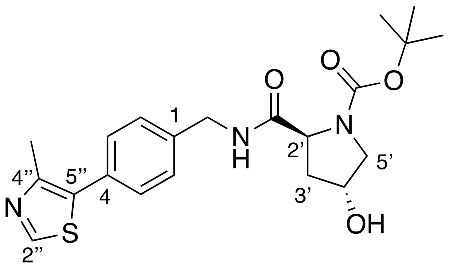

To a 25 mL flame-dried round-bottom flask equipped with a magnetic stir bar and rubber septum were added *trans*-*N*-(*tert*-butoxycarbonyl)-4-hydroxy-L-proline (**12**) (0.615 g, 2.60 mmol), HATU (0.130 g, 3.38 mmol), DIPEA (1.60 mL, 9.11 mmol), and 13 mL of anhydrous DMF. Amine **13** (0.597 g, 2.86 mmol) dissolved in anhydrous DMF (13 mL) was added in two portions to the reaction flask. The resulting solution was stirred at room temperature for 19 h. The reaction was quenched by adding deionized water (26 mL), and the aqueous phase was extracted using EtOAc until no product was evident in the extracting solvent by TLC (typically 6 times). The combined organic phases were dried over anhydrous sodium sulfate, filtered, and concentrated by rotary evaporation at 35 °C. The product was purified by flash column chromatography using oven-dried silica and a 5-7% MeOH in CH_2Cl_2 gradient to afford **15** as an off-white foamy solid (0.788 g, 1.89 mmol, 73%).

m.p.: 78-80 °C. **R$_f$**: 0.45 (5% MeOH in CH_2Cl_2).

FT-IR (KBr plates) ν_{max} (cm^{-1}): 3305, 3076, 2978, 2930, 1672, 1546, 1409, 1162, 858, 755.

^{1}H NMR (500 MHz, CD_3OD, major and minor rotamer, NH and OH signals not evident in ^{1}H NMR spectrum) δ (ppm): 8.874 and 8.865 (each s, 1H, C**H** thiazole of major and minor rotamers), 7.43 and 7.42 (each s, 4H, major and minor rotamer, **2**-Ar-**H** and **3**-Ar-**H**), 4.63 – 4.20 (m, 4H, Ar-C**H**$_2$-N, O=C-C**H**$_2$-N, (CH_2)C**H**-OH), 3.64 – 3.54 (m, 1H, **5´**-C**H**$_2$), 3.54 – 3.43 (m, 1H, **5´**-C**H**$_2$), 2.47 (s, 3H, C**H**$_3$), 2.34 – 2.20 (m, 1H, **3´**-C**H**$_2$), 2.03 (ddd, *J* = 13.1, 8.6, 4.5 Hz, 1H, **3´**-C**H**$_2$), 1.47 and 1.33 (s each, 9H, OC(C**H**$_3$)$_3$, minor and major rotamer).

^{13}C NMR (126 MHz, CD_3OD, major rotamer) δ (ppm): 175.56 (**C**ONH), 156.17 (N**C**O), 152.92 (**C**H thiazole), 149.11 (**C**-4´´ thiazole), 140.31 (**C**-1-Ar), 133.25 and 131.86 (**C**-4 and **C**-5´´ Thiazole), 130.51 and 129.69 (**C**-2 and **C**-3-Ar), 81.58 (O**C**(CH_3)$_3$), 70.05 ((CH_2)**C**H-OH), 60.81 (O=C-**C**H-N), 56.00 ((**5´´**-**C**H_2)CHOH), 43.78 (Ar-**C**H_2-N), 40.86 ((**3´´**-**C**H_2)CH-OH), 28.54 (OC(**C**H_3)$_3$), 15.79 (**C**H_3 thiazole).

HRMS QToF-ESI: calculated for $C_{21}H_{28}N_3O_4S$ [M+H$^+$] *m/z* 418.1801; found *m/z* 418.1808.

***tert*-Butyl-((*S*)-1-((2*S*,4*R*)-4-hydroxy-2-((4-(4-methylthiazol-5-yl)benzyl)carbamoyl)pyrolidin-1-yl)-3,3-dimethyl-1-oxobutan-2-yl)carbamate (26)**

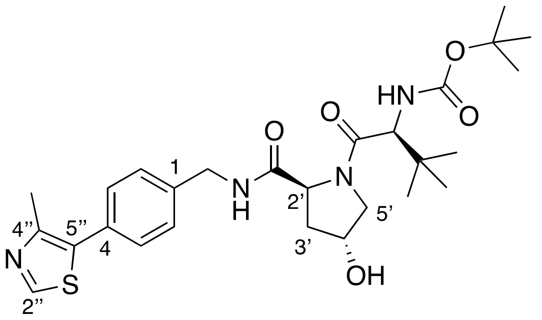

To a 50 mL flame-dried round-bottom flask equipped with a magnetic stir bar and rubber septum were added (*S*)-*N*-Boc-2-amino-3,3-dimethylbutyric acid (**16**) (0.264 g, 1.11 mmol), HATU (0.556 g, 1.45 mmol), DIPEA (0.66 mL, 3.90 mmol), and 5 mL of anhydrous DMF. Amine **13** (0.390, 2.86 mmol) dissolved in anhydrous DMF (6 mL) was added in two portions to the reaction flask. The resulting solution was stirred at room temperature for 20 h. The reaction was quenched by adding deionized water (11 mL), and the aqueous phase was extracted using EtOAc (10 mL) until no product was evident in the extracting solvent by TLC (typically 5 times). The combined organic phases were dried over anhydrous sodium sulfate, filtered, and concentrated by rotary evaporation at 35 ^oC. The product was purified by flash column chromatography using oven-dried silica and a 5-7% MeOH in CH_2Cl_2 gradient to afford **15** as an off-white foamy solid (0.525 g, 0.989 mmol, 81%)

m.p: 161-163 ^oC. **R$_f$:** 0.45 (5% MeOH in CH_2Cl_2).

FT-IR (KBr plates) ν_{max} (cm^{-1}): 3445, 3423, 3312, 3078, 2970, 2873, 1686, 1631, 1551, 1504, 1440, 1368, 1231, 1166, 763.

^{1}H NMR (500 MHz, $CDCl_3$, major and minor rotamer, OH signal not evident in ^{1}H NMR spectrum) δ (ppm): 8.67 (s, 1H, C**H** thiazole), 7.48 (t, *J* = 6.0 Hz, 1H, N**H**C=O), 7.33 (d, *J* = 8.3 Hz, 2H, Ar-**H**), 7.30 (d, *J* = 8.3 Hz, 2H, Ar-**H**), 5.23 (d, *J* = 9.1 Hz, 1H, N**H**C=O), 4.72 (t, *J* = 7.8 Hz, 1H, O=C-C**H**-N proline), 4.53 (dd, *J* = 15.0, 6.5 Hz, 1H, Ar-C**H**$_2$-N), 4.51 (m, s br, 1H, (CH_2)C**H**-OH)), 4.28 (dd, *J* = 15.0, 5.2 Hz, 1H, Ar-C**H**$_2$-N), 4.17 (d, *J* = 9.2 Hz, 1H, O=C-C**H**-N *tert*-leucine), 3.98 (d, *J* = 11.2 Hz, 3.5 Hz, 1H, **5´**-C**H**$_2$), 3.61 (dd, *J* = 11.3, 3.8 Hz, 1H, **5´**-C**H**$_2$), 2.49 (s, 3H, C**H**$_3$ thiazole overlapping signal with **H** from C**H**$_2$), 2.47-2.44 (m, overlapping signal with CH_3, 1H, **3´**-C**H**$_2$), 2.11-2.04 (m, 1H, **3´**-C**H**$_2$), 1.39 (s, 9H, major and minor rotamer, OC(C**H**$_3$)$_3$), 0.91 (s, 9H, major rotamer, C(C**H**$_3$)$_3$).

^{13}C NMR (126 MHz, $CDCl_3$, major rotamer) δ (ppm): 172.56 (**C**ONH proline), 170.93 (**C**ONH *tert*-leucine), 156.636 (N**C**O), 150.48 (**C**-2'' thiazole), 148.47 (**C**-4'' thiazole), 138.23 (**C**-1 Ar),

131.74 and 130.74 (**C**-1 Ar or **C**-5’’ thiazole), 129.56 and 128.11 (**C**-2 and **C**-3-Ar), 80.39 (O**C**(CH_3)$_3$), 70.15 ((CH_2)**C**H-OH), 58.97 (O=C-**C**H-N *tert*-leucine), 58.59 (O=C-**C**H-N proline), 56.59 (**5´**-**C**H_2)CH-OH), 43.28 (Ar-**C**H_2-N), 36.11 (**5´**-**C**H_2)CH-OH), 35.16 (**C**(CH_3)$_3$), 28.42 (OC(**C**H_3)$_3$, 26.41 C(**C**H_3)$_3$, 16.10 (**C**H_3 thiazole).

HRMS QToF-ESI: calculated for $C_{27}H_{39}N_4O_5S$ [M+H$^+$] *m/z* 531.2641; found *m/z* 531.2648.

3. Characterization Data of Identified Reaction Byproducts

4,4'-(4-Methylthiazole-2,5-diyl)dibenzonitrile (23)

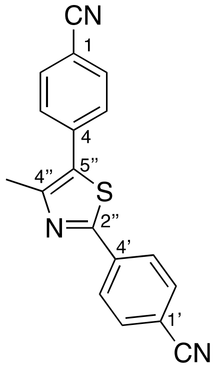

Yellow solid isolated from C—H arylation using **5**, **6**, and catalytic Pd(OAc)$_2$ (See manuscript Table 1, entries 1 and 2).

m.p.: 212-214 °C.

R$_f$: 0.85 (30% EtOAc in Hexanes)

FT-IR (KBr plate) ν_{max} (cm^{-1}): 2913, 2849, 2223, 1605, 1491, 1441, 1405, 1375, 836.

^{1}H NMR (500 MHz, $CDCl_3$) δ (ppm): 8.05 (d, *J* = 8.5 Hz, 2H, 3´-Ar-**H**), 7.81-7.66 (m, 4H, 2 and 2´´Ar-**H**), 7.60 (d, *J* = 8.3 Hz, 2H, 3-Ar-**H**), 2.60 (s, 3H, C**H**$_3$).

^{13}C NMR (126 MHz, $CDCl_3$) δ (ppm): 163.80(**C**-2´´ thiazole**)**, 151.39(**C**-4´´ thiazole**)**, 137.13 (**C**-4´ Ar), 136.54 (**C**-4 Ar), 132.98 and 132.77 (**C**-2 and **C**2´´ Ar), 132.19 (**C**-5´´ thiazole**)**, 129.71 (**C**-3 Ar), 126.90 (**C**-**3´** Ar), 118.51 (**C**N), 113.58, (**C**-1´ Ar) 111.92 (**C**-1 Ar), 16.76 (**C**H_3).

HRMS QToF-ESI: calculated for $C_{18}H_{12}N_3S$ [M+H$^+$] *m/z*: 302.0752; found *m/z*: 302.0742

4,4'-dimethyl-5,5'-bithiazole (24)

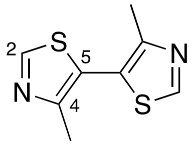

Yellow solid isolated from C—H arylation using **5**, **6**, and catalytic Pd(OAc)$_2$ (see Tables 1-3. Table 1 and 2 entries 1 and 2, Table 3 entries 1 to 4 and 12)

m.p.: 99-100 °C.

R$_f$: 0.60 (30% EtOAc in Hexanes).

FT-IR (KBr plate) ν_{max} (cm^{-1}): 3054, 2987, 2954, 2917, 2854, 1409, 1370, 1308.

^{1}H NMR (500 MHz, $CDCl_3$) δ (ppm): 8.80 (s, 1H, C**H** thiazole), 2.40 (s, 3H, C**H$_3$**).

^{13}C NMR (126 MHz, $CDCl_3$) δ (ppm): 152.83 (2-**C**H), 152.73(3-**C**H), 120.74 (5-**C**H), 16.00 (**C**H_3).

HRMS QToF-ESI: calculated for $C_8H_9N_2S_2{}^+$ [M+H$^+$] *m/z* 197,0202; found *m/z* 197,0202.

***tert*-Butyl (4-(4-methylthiazol-2-yl)benzyl)carbamate (27)**

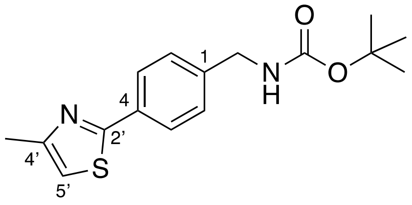

White solid isolated from C—H arylation using **6**, **7**, and catalytic $Pd(OAc)_2$ (Table 2, entry 1).

m.p.:123-124 °C

R$_f$: 0.80 (30% EtOAc in Hexanes)

FT-IR (KBr plate) ν_{max} (cm^{-1}): 3312, 3104, 3043, 3001, 2922, 2978, 1683, 1531, 1287, 1162, 1140 858, 755.

^{1}H NMR (500 MHz, $CDCl_3$) δ (ppm): 7.89 (d, *J* = 8.1 Hz, 2H, 2-Ar-**H**), 7.33 (d, *J* = 8.1 Hz, 2H, 3-Ar-**H**), 6.86 (q, *J* = 0.9 Hz, 1H, C**H** thiazole), 4.89 (br s, 1H, N**H**), 4.35 (d, *J* = 5.4 Hz, 2H, ArC**H$_2$**N), 2.50 (d, *J* = 0.9 Hz, 3H, C**H$_3$**), 1.47 (s, 9H, OC(C**H**$_3$)$_3$).

^{13}C NMR (126 MHz, $CDCl_3$) δ (ppm): 167.39 (**C**-2´ thiazole), 156.04 (**C**O), 153.99 (**C**-4´ thiazole), 140.91 (**C**1-Ar), 133.06 (**C**-4 Ar), 128.04 (**C**-2 Ar), 126.84 (**C**-3-Ar), 113.51 (**C**H thiazole), 79.82 (O**C**(CH_3)$_3$), 44.53 (**C**H_2), 28.54 (OC(**C**H_3)$_3$), 17.42 (**C**H_3).

HRMS QToF-ESI: calculated for $C_{16}H_{21}N_2O_2S$ [M+H$^+$] *m/z* 305.1318; found *m/z* 305.1312.

Di-*tert*-Butyl(((4-methylthiazole-2,5-diyl)bis(4,1-phenylene))bis(methylene))dicarbamate (28)

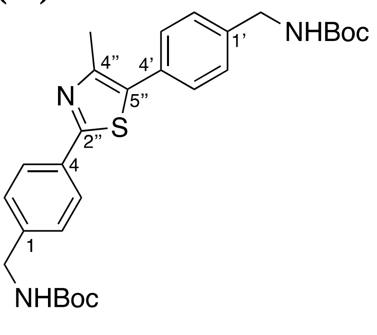

Beige solid isolated from C—H arylation using **6**, **7**, and catalytic $Pd(OAc)_2$ (Table 2, entry 1).

m.p.: 125-127 °C

R$_f$: 0.80 (50% EtOAc in Hexanes)

FT-IR (KBr plate) ν_{max} (cm^{-1}): 3339, 2977, 2930, 1697, 1520, 1273, 1247, 1169.

^{1}H NMR (500 MHz, $CDCl_3$) δ (ppm): 7.82 (d, *J* = 8.1 Hz, 1H, 3-Ar-**H**), 7.38 (d, *J* = 8.1 Hz, 2H, 3´-Ar-**H**), 7.24-7.31 (m, 4H, 2-Ar-**H** and 2´-Ar-**H**), 4.83 (s, 2H, N**H**), 4.29 (s, 4H, C**H$_2$**), 2.47 (s, 3H, C**H$_3$** thiazole), 1.41 (s, 18H, OC(C**H**$_3$)$_3$).

^{13}**C NMR** (126 MHz, $CDCl_3$) δ (ppm): 164.82 (**C**-2´´ thiazole), 156.05 (N**C**O), 149.00 (**C**-4´´ thiazole), 140.95 (**C**-1 Ar), 138.88 (**C**1 Ar), 132.94 (**C**4-Ar), 131.98 (**C**-5´ thiazole), 131.35 (**C**-4´Ar), 129.52 (**C**-3´-Ar), 128.07 (**C**-2 Ar), 127.90 (**C**-2 Ar), 126.68 (**C**-3 Ar), 79.84 (O**C**(CH_3)$_3$), 44.47 (Ar**C**H_2N), 28.56 (OC(**C**H_3)$_3$), 16.51 (**C**H_3).

HRMS QToF-ESI: calculated for $C_{28}H_3$ N_3O_4S [M+H$^+$] *m/z* 510.2427; found *m/z* 510.2423.

di-*tert*-Butyl 5,5'-(((((4-methylthiazole-2,5-diyl)bis(4,1-phenylene))bis(methylene))bis (azanediyl))bis(carbonyl))(3*R*,3'*R*,5*S*,5'*S*)-bis(3-hydroxypyrrolidine-1-carboxylate) (30)

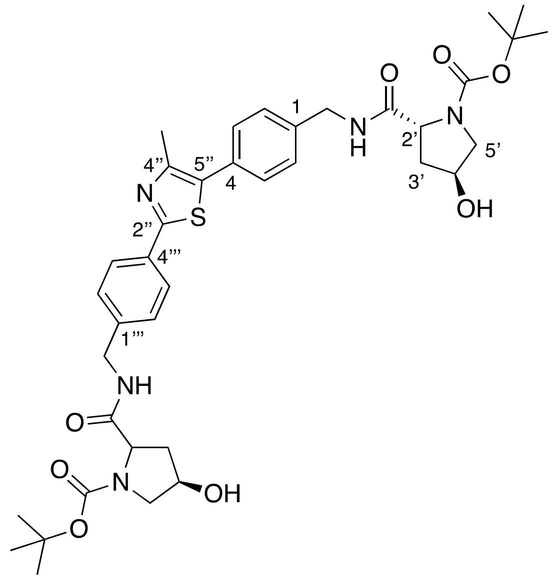

Yellow oil isolated from C—H arylation using **6**, **29**, and catalytic $Pd(OAc)_2$ (Table 3, entry 1).

R$_f$: 0.70 (10% MeOH in DCM).

FT-IR (KBr plates), ν_{max} (cm^{-1}): 3338, 2977, 2928, 1662, 1546, 1454, 1410, 1366, 1160

^{1}**H NMR** (500 MHz, CD_3OD) δ (ppm): 8.79 – 8.48 (m, 1H, N**H**), 7.89 (d, *J* = 8.2 Hz, 1H, 3´´Ar-H), 7.64 – 7.33 (m, 7H, Ar-H), 4.61 – 4.43 (m, 2H, ArC**H$_2$**N), 4.43-4.28 (m, 6H, ArC**H$_2$**N, O=C-C**H**-N), 3.70 – 3.41 (m, 4H, 5´-C**H$_2$**), 2.50 (s, 3H,C**H$_3$**), 2.25 (m, 1H, 3´-C**H$_2$**), 2.15 – 1.89 (m, 1H, 3´-C**H$_2$**), 1.57 – 0.91 (m, 18H, OC(C**H$_3$**)$_3$).

^{13}**C NMR** (126 MHz, CD_3OD, major rotamer) δ (ppm): 175.58 (**C**ONH), 166.60 (**C**-2´´ thiazole), 156.22(N**C**O), 149.78(**C**-4´´thiazole), 142.45(**C-1**´´´ Ar), 140.25(**C-1** Ar)**,** 133.60(**C-4** Ar)**,** 132.01(**C**-5´´ thiazole), 130.40 (**C**-Ar), 129.67 (**C**-Ar), 128.94 (**C**-Ar), 128.06 (**C**-Ar), 127.50 (**C**-Ar), 81.63 (O**C**(CH_3)$_3$), 70.08 ((CH_2)**C**HOH), 60.87 (O=C-**C**H-N), 56.00 (**5´**-**C**H_2)CHOH), 43.94 (Ar-**C**H_2-N), 40.85 (3´-**C**H_2)CHOH), 28.52 (OC(**C**H_3)$_3$), 16.13 (**C**H_3 thiazole).

HRMS QToF-ESI: calculated for $C_{38}H_{49}N_5O_8SNa^+$[M+Na$^+$] *m/z* 758.3194; found *m/z* 758.3199.

2-((3*R*,5*S*)-5-((4-bromobenzyl)carbamoyl)-1-(tert-butoxycarbonyl)pyrrolidin-3-yl)1-(tert-butyl) (2*S*,4*R*)-4-hydroxypyrrolidine-1,2-dicarboxylate (33)

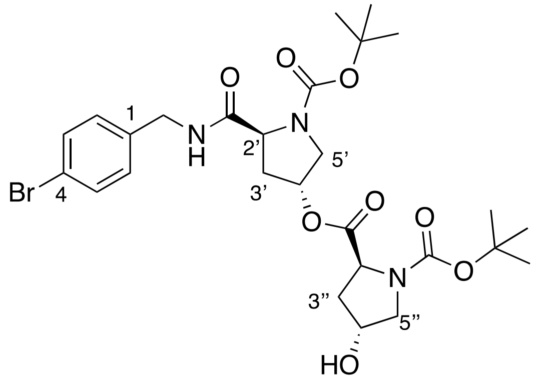

Yellow oil from benzylic amine amidation using 4-bromobenzylamine (**4**) (1.1 equiv), *N*-(*tert*-butoxycarbonyl)-4-hydroxy-L-proline (**12**) (1.0 equiv), EDC•HCl (1.3 equiv), HOBt (1.3 equiv), DIPEA (2.3 equiv), and neat DMF as the solvent (0.25 M).

R_f: 0.40 (5% MeOH in DCM).

FT-IR (KBr plate) ν_{max} (cm^{-1}): 3323,3087, 2972, 2928, 1745, 1682, 1541, 1408, 1253, 1158, 1132.

^{1}H NMR (500 MHz, MeOD, major and minor rotamer) δ (ppm): 8.51 (m, 1H, N**H**), 7.51 – 7.26 (m, 2H,, Ar-H), 7.22 – 7.05 (m, 2H, Ar-**H**), 5.20 (s, 1H, ((CH_2)C**2´H**OH)), 4.40 – 4.09 (m, 5H, O=C-C**H**-N, ArC**H₂**N, (CH_2)**C-4´H**OH), 3.82 – 3.50 (m, 2H, **5´-**C**H₂**), 3.50 – 3.22 (m, 2H, 5´´-C**H₂**), 2.43 – 2.21 (m, 1H, 3´-C**H₂**), 2.20 – 2.03 (m, 2H, 3´-C**H₂** and 3´´-C**H₂**), 1.99 – 1.79 (m, 1H, 3´´-C**H₂**), 1.26 (4 s, each, 18H, OC(C**H₃**)₃).

^{13}C NMR (126 MHz, CD_3OD, major rotamer) δ (ppm): 174.79 (**C**ONH), 174.09 (**C**OO), 156.22 (N**C**O), 155.75 (N**C**O), 139.18 (**C1**-Ar), 132.65 and 131.01 (**C**-2 and **C**-3 Ar), 122.09 (**C-B**r Ar), 81.87 (O**C**(CH_3)₃), 81.59 (O**C**(CH_3)₃), 74.62 ((CH_2)**C**HOOC), 69.88 ((CH_2)**C**HOH), 60.54 (OOC-**C**H-N), 59.26 (O=C-**C**H-N), 55.88 (5´**-C**H_2)CHOH), 53.52 (5´´**-C**H_2)CHOH), 43.66 (Ar-**C**H_2-N), 39.95 and 38.02 ((3´-**C**H_2)CHOH and 3´´-**C**H_2)CHOH), 28.67 and 28.51 (OC(**C**H_3)₃).

HRMS QToF-ESI: calculated for $C_{27}H_{38}BrN_3NaO_8^+$ [M+Na^+] *m/z* 634.1734; found *m/z* 634.1725

4. References

1. N. R. Babij, E. O. McCusker, G. T. Whiteker, B. Canturk, N. Choy, L. C. Creemer, C. V. D. Amicis, N. M. Hewlett, P. L. Johnson, J. A. Knobelsdorf, F. Li, B. A. Lorsbach, B. M. Nugent, S. J. Ryan, M. R. Smith and Q. Yang, *Org. Process Res. Dev.*, 2016, **20**, 661-667, DOI: **10.1021/acs.oprd.5b00417**.
2. C. Galdeano, M. S. Gadd, P. Soares, S. Scaffidi, I. Van Molle, I. Birced, S. Hewitt, D. M. Dias and A. Ciulli, *J. Med. Chem.*, 2014, **57**, 8657-8663, DOI: **10.1021/jm5011258**.
3. L.-Q. Hu, R.-L. Deng, Y.-F. Li, C.-J. Zeng, D.-S. Shen and F.-S. Liu, *Organometallics*, 2018, **37**, 214-226, DOI: **10.1021/acs.organomet.7b00784**.
4. M. Gütschow, C. Steinebach, S. A. Voell, L. P. Vu, A. Bricelj, I. Sosič and G. Schnakenburg, *Synthesis*, 2020, **52**, 2521-2527, DOI: **10.1055/s-0040-1707400**.
5. W. Yan, B. S. Pan, J. Shao, H. K. Lin and H. Y. Li, *ACS Omega*, 2022, **7**, 26015-26020, DOI: **10.1021/acsomega.2c00245**.
6. M. S. Cooper, M. C. Norley, S. Armitage, J. O. Cresser-Brown, A. K. Edmonds, S. Goggins, J. P. Hopewell, B. Karadogan, K. A. Knights, T. J. Nash, C. S. Oakes, W. J. O'Neill, S. J. Pridmore, H. J. Maple and G. P. Marsh, *Org. Biomol. Chem.*, 2023, **21**, 8344-8352, DOI: **10.1039/d3ob00983a**.

7. G. Amberchan, R. A. Snelling, E. Moya, M. Landi, K. Lutz, R. Gatihi and B. Singaram, *J. Org. Chem.*, 2021, **86**, 6207-6227, DOI: **10.1021/acs.joc.0c03062**.

5. Appearance of 9, 10 and Isolated Byproducts 23, 24, 27, and 28

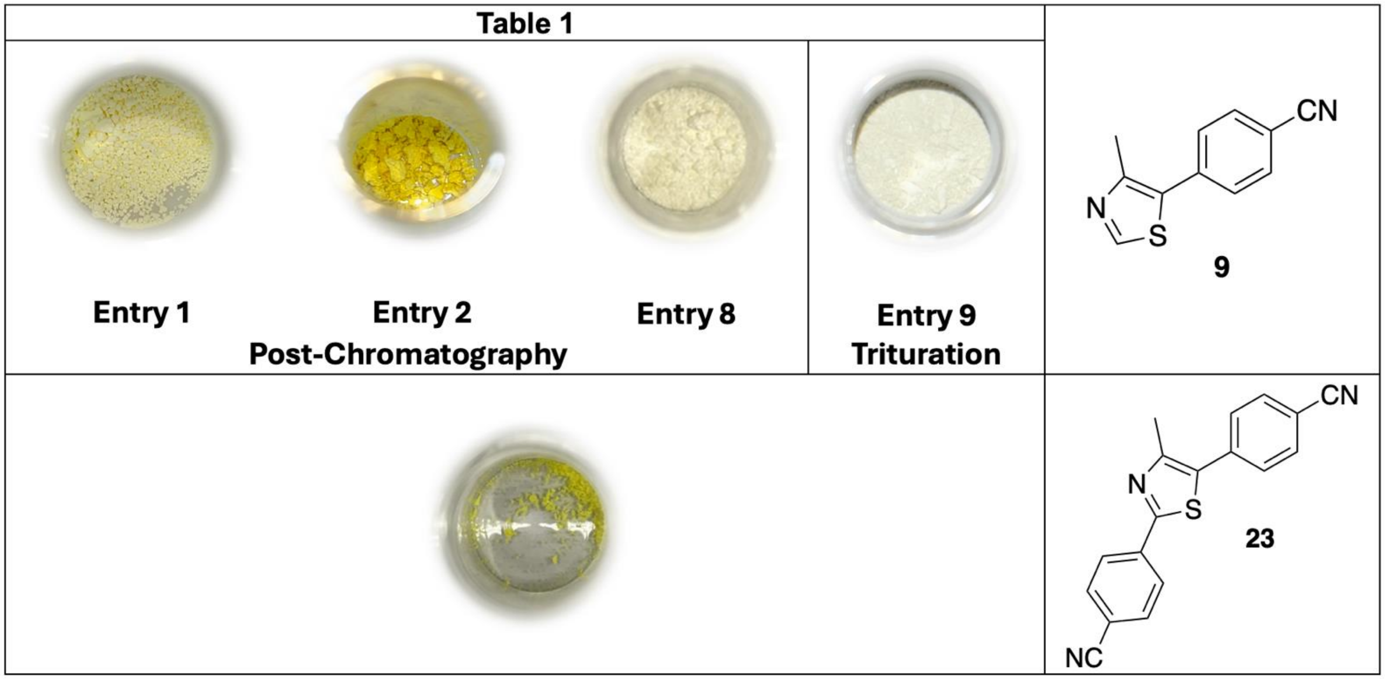

Figure 1S. Products isolated through C—H arylation of **6** with **5** using $Pd(OAc)_2$ and Pd-PEPPSI-IPr

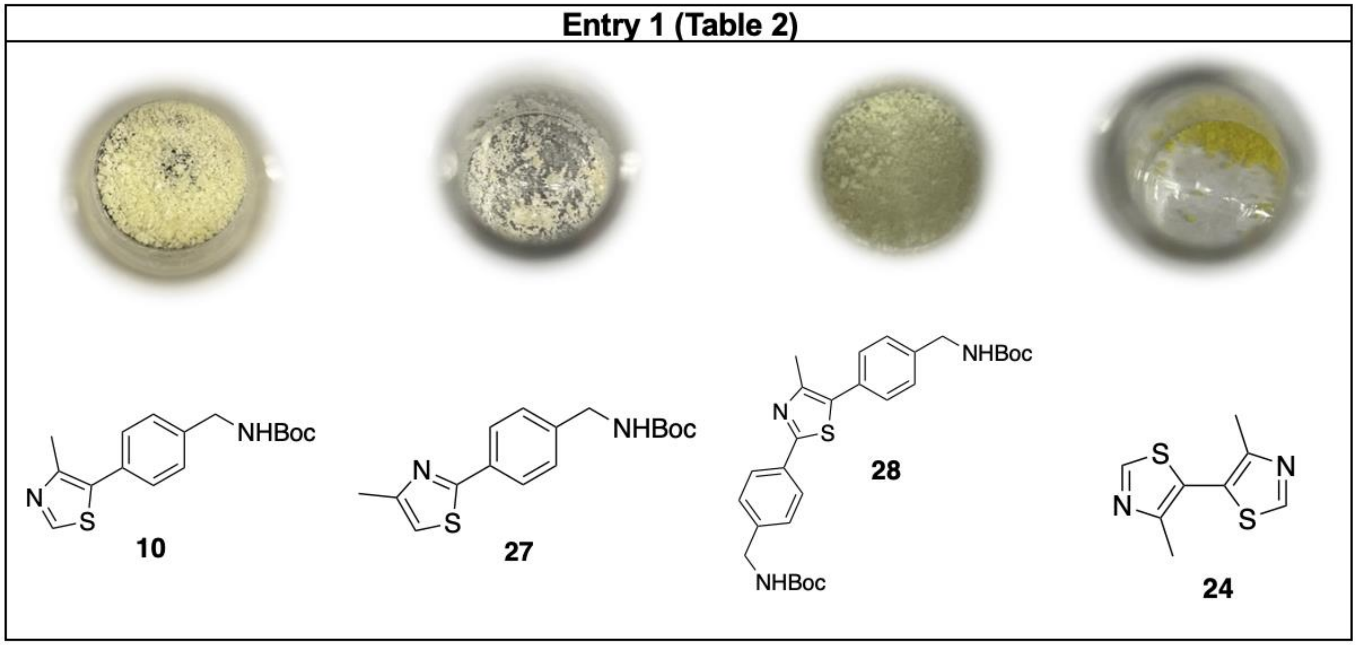

Figure 2S. Products isolated through C—H arylation of **6** with **7** using $Pd(OAc)_2$.

6. ^{1}H, ^{13}C NMR spectra

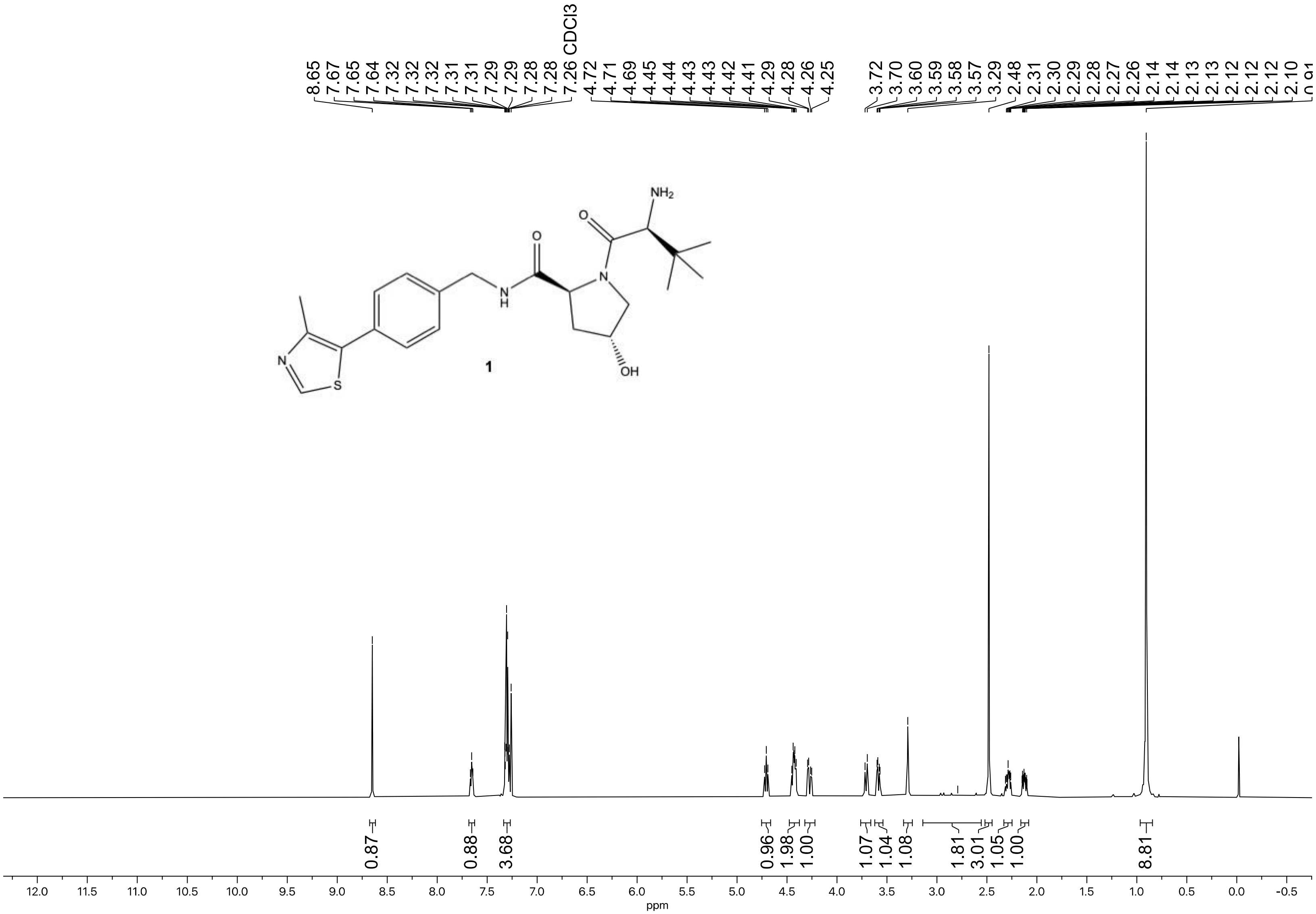

^{1}H NMR (500 MHz, $CDCl_3$) spectrum of **1**

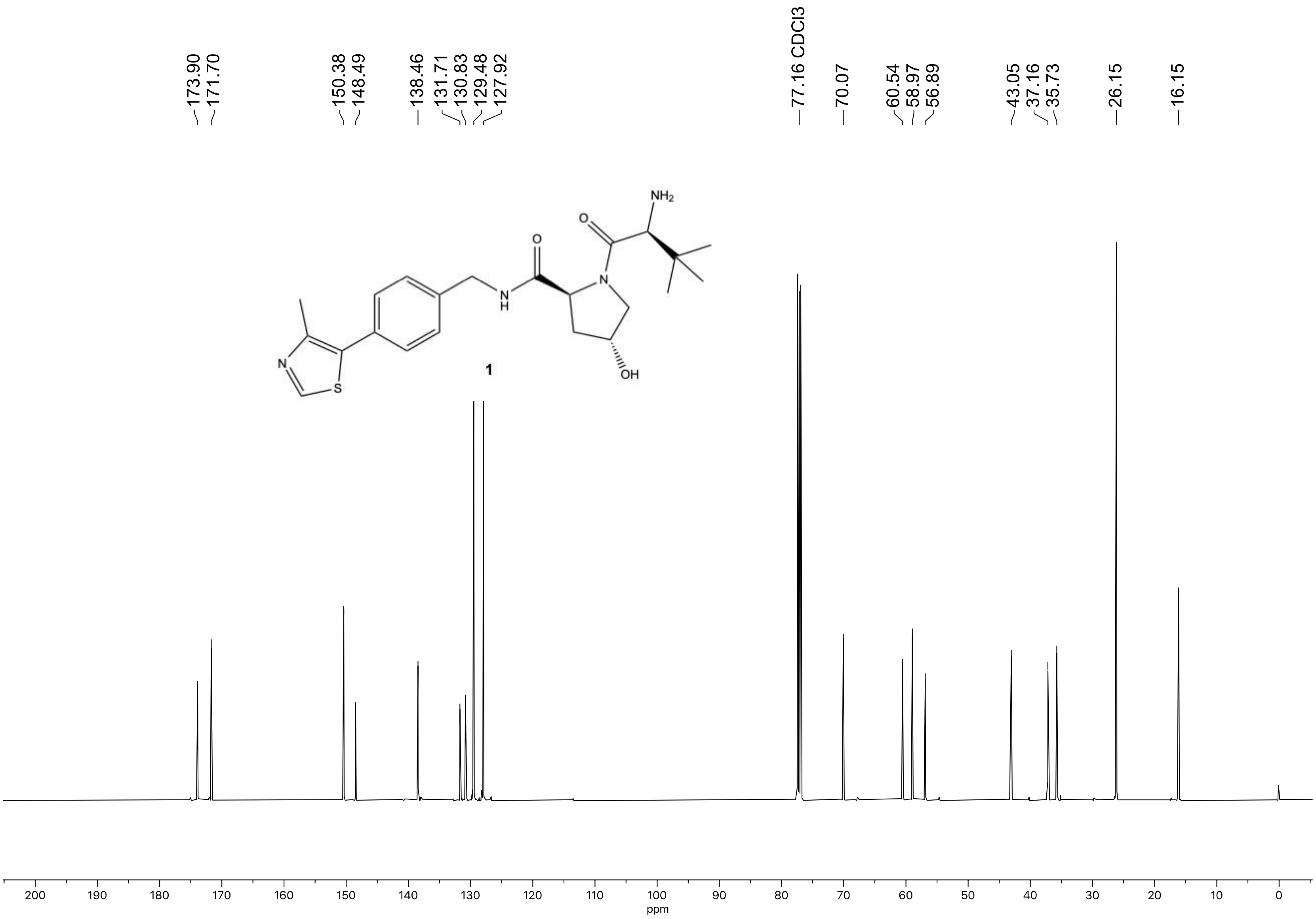

^{13}C NMR (126 MHz, $CDCl_3$) spectrum of **1**

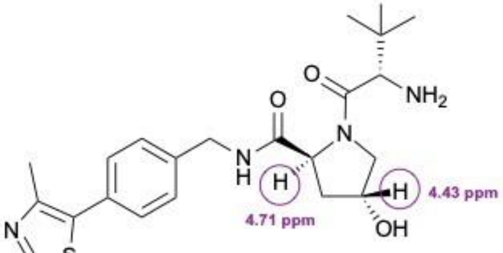

1

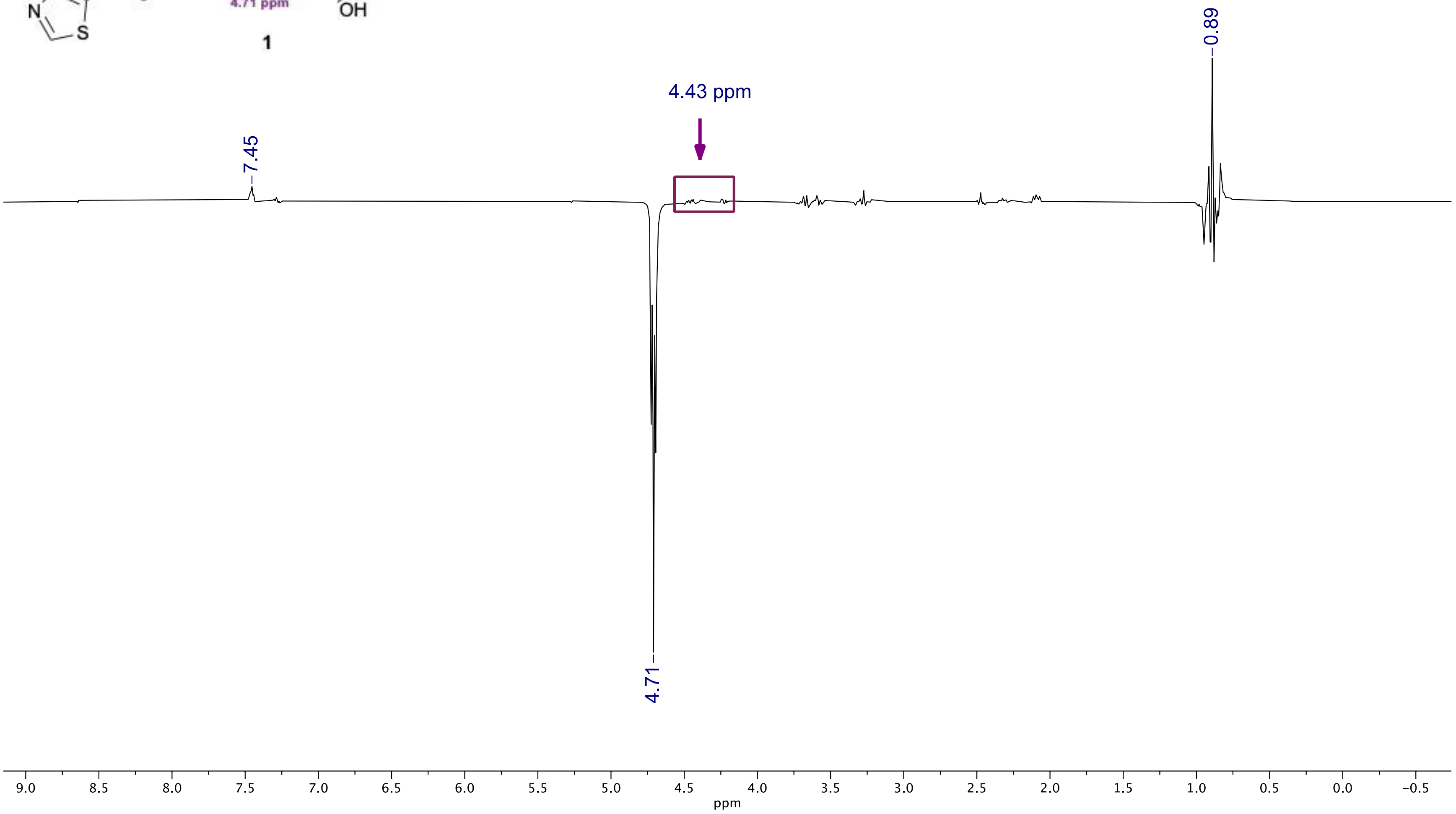

[1]D NOE NMR (500 MHz, $CDCl_3$) spectrum of **1**

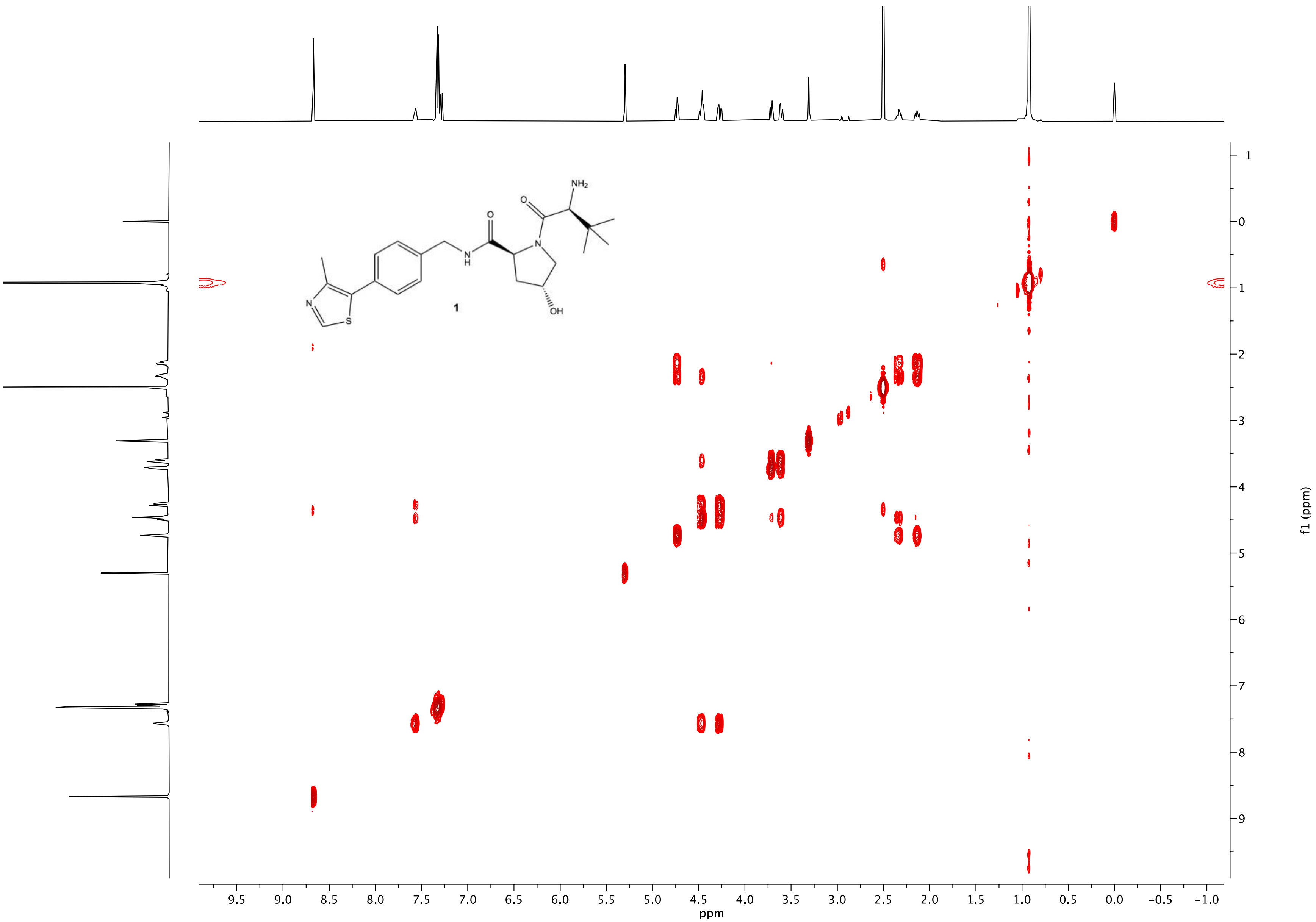

^{1}H-^{1}H COSY NMR (500 MHz, $CDCl_3$) spectrum of **1**

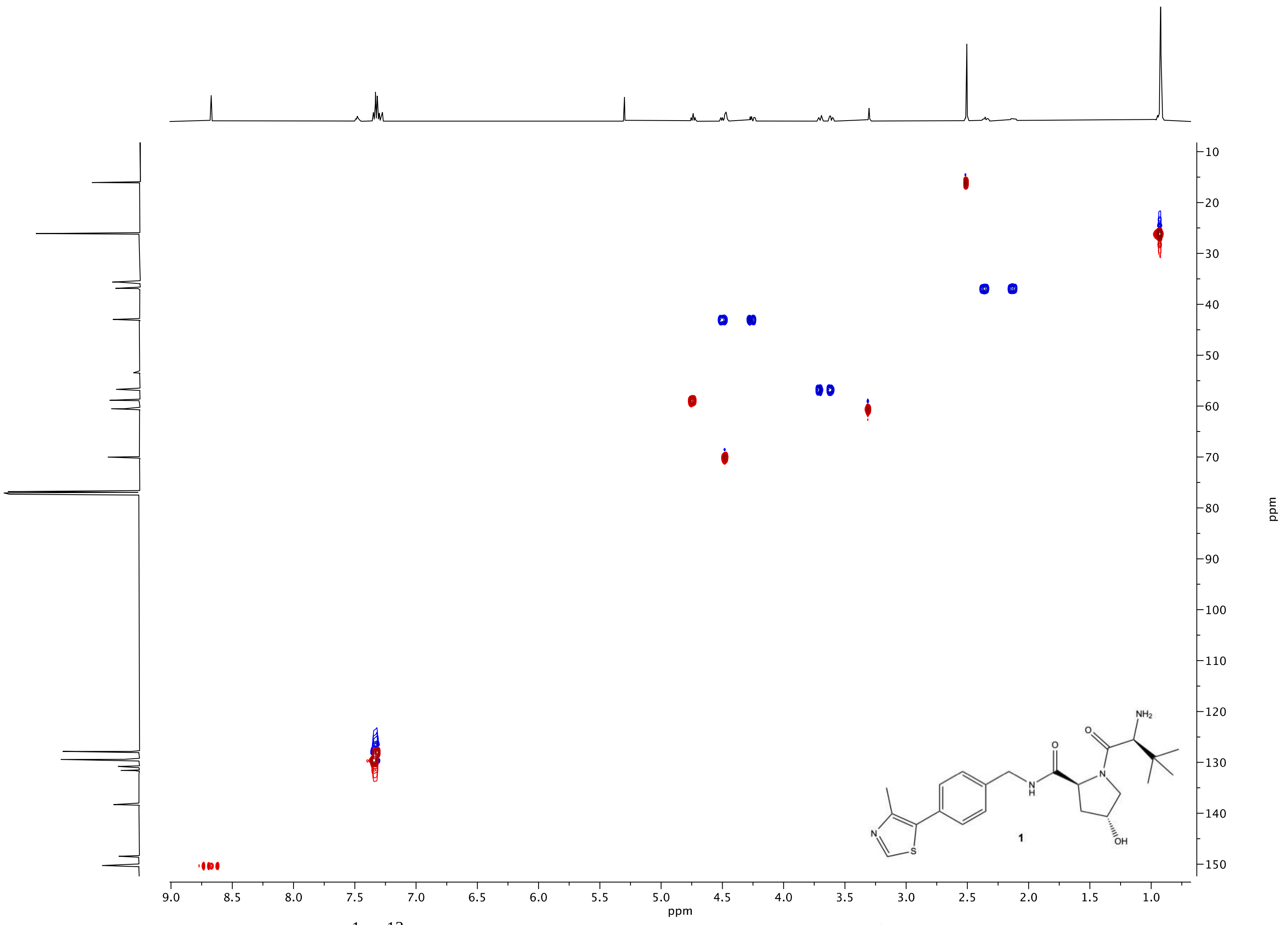

^{1}H-^{13}C HSQC NMR (500 MHz, $CDCl_3$) spectrum of **1**

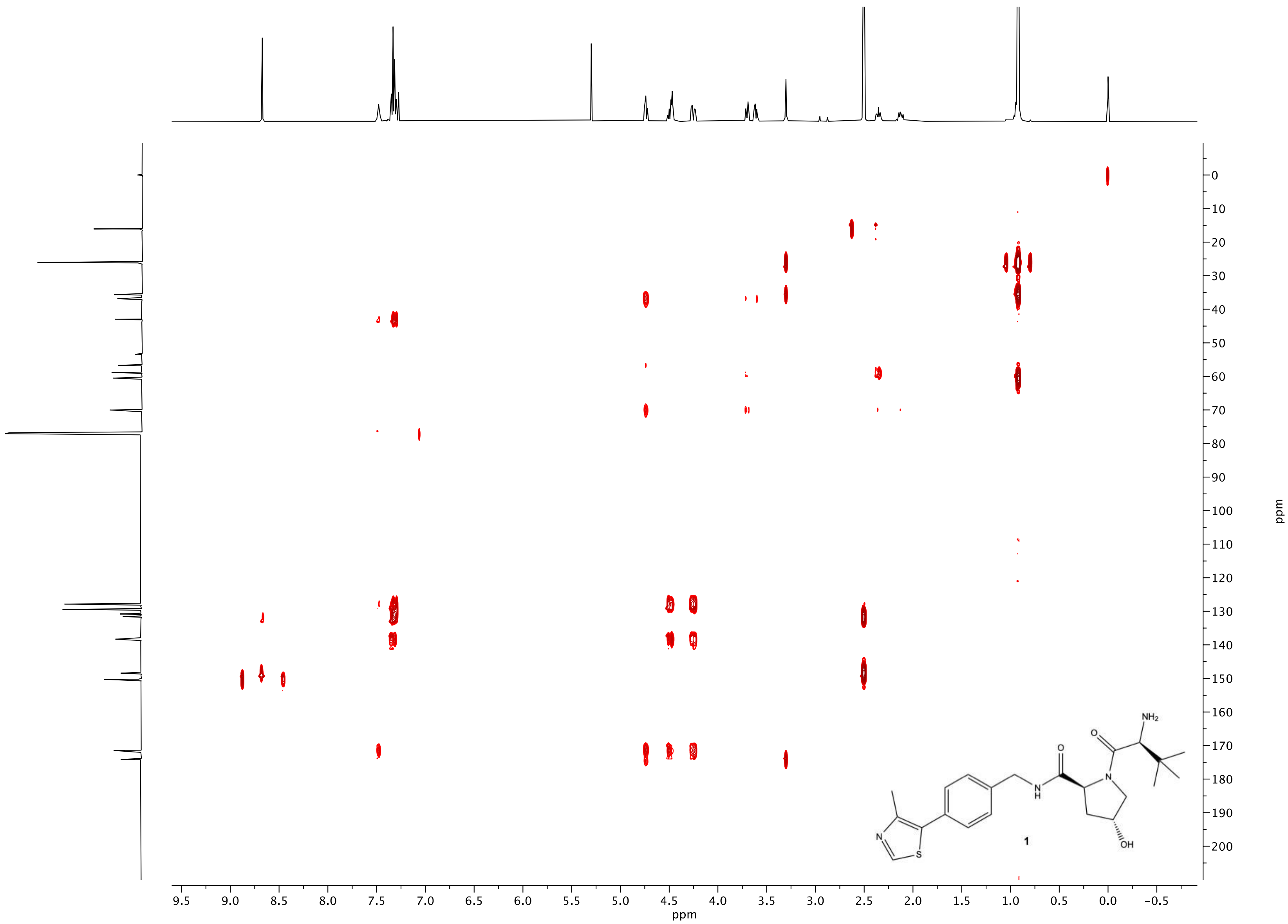

^{1}H-^{13}C HMBC NMR (500 MHz, $CDCl_3$) spectrum of **1**

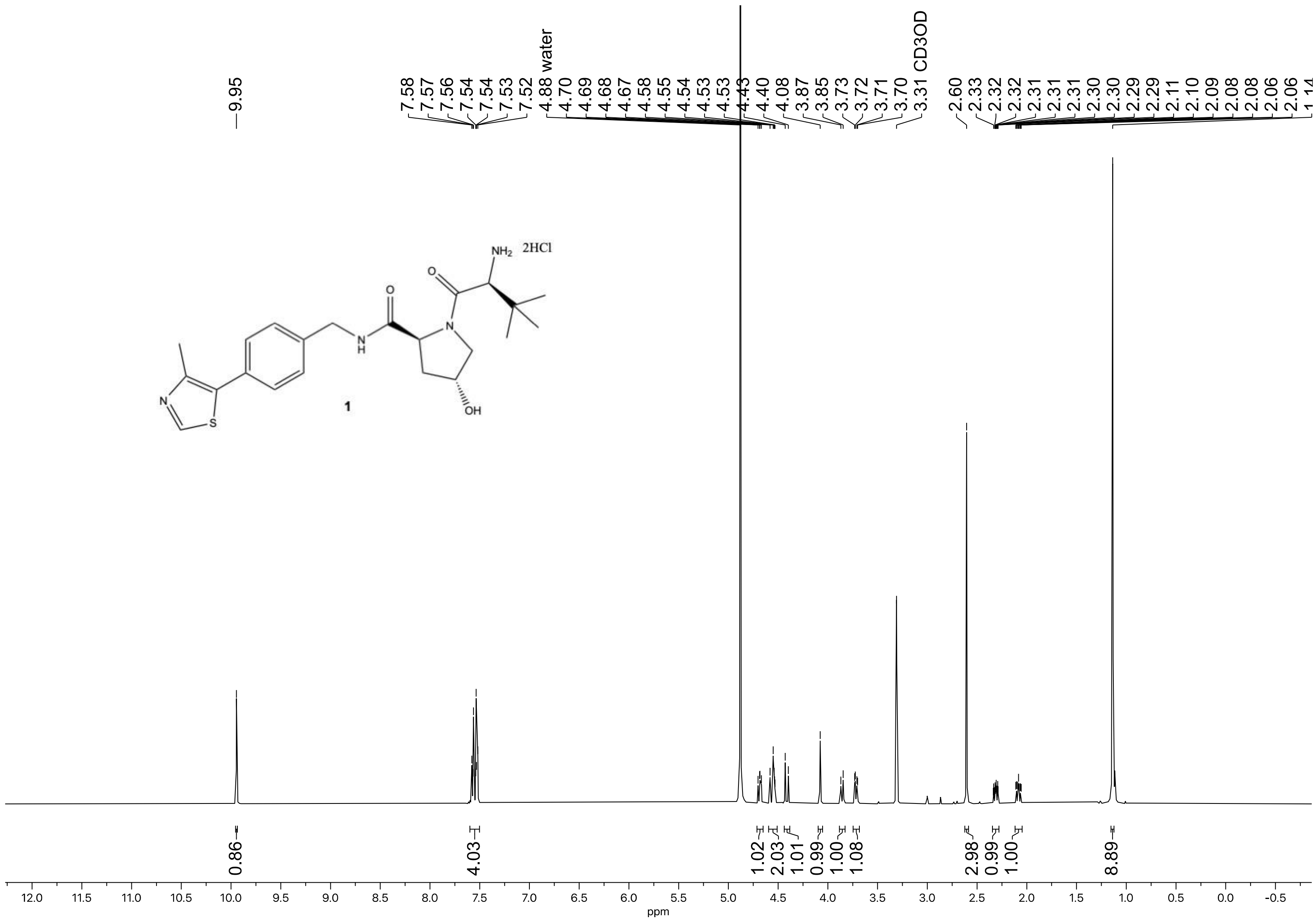

^{1}H NMR (500 MHz, CD_3OD) spectrum of **1** (hydrochloride salt)

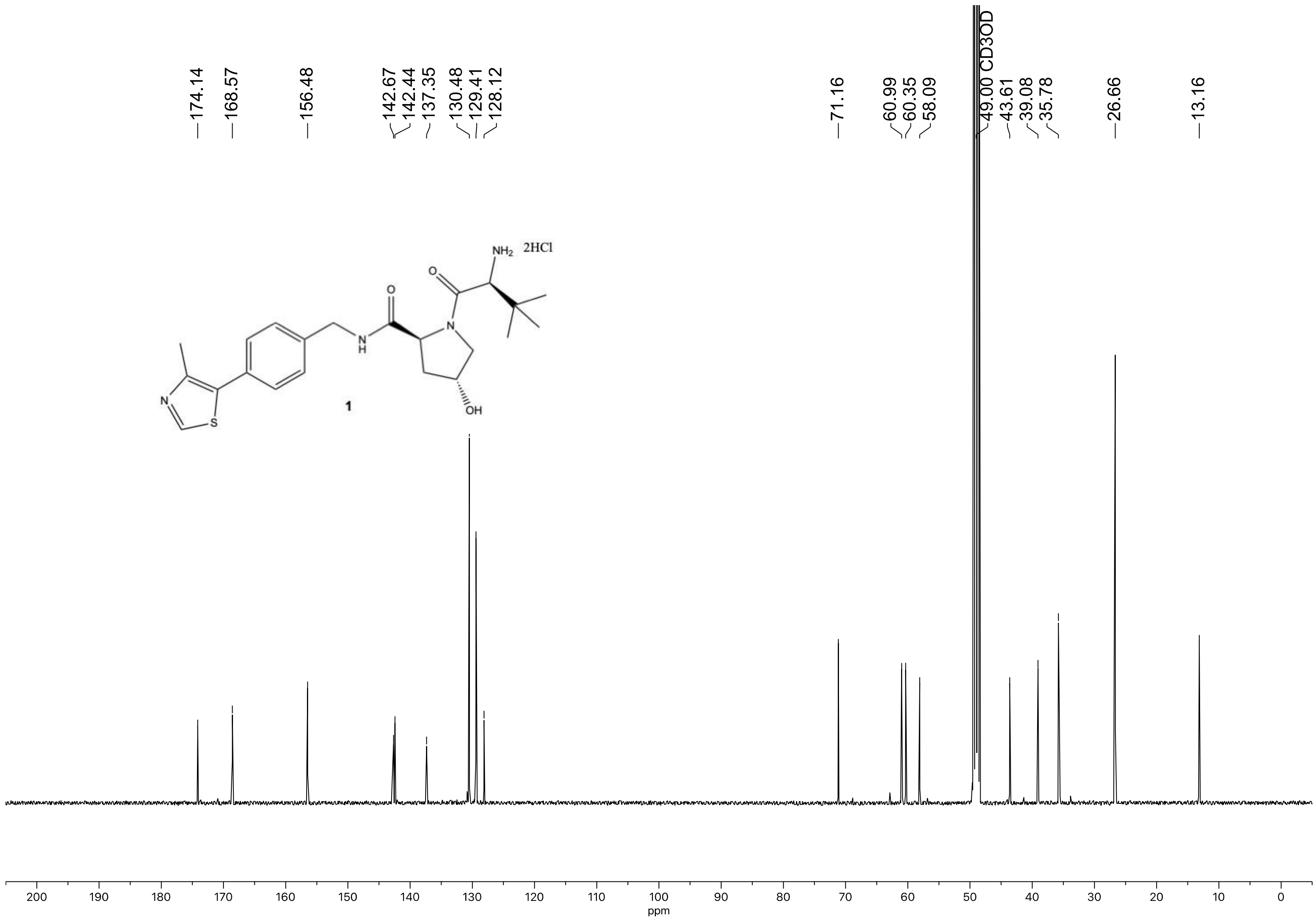

^{13}C NMR (126 MHz, CD_3OD) spectrum of **1** (hydrochloride salt)

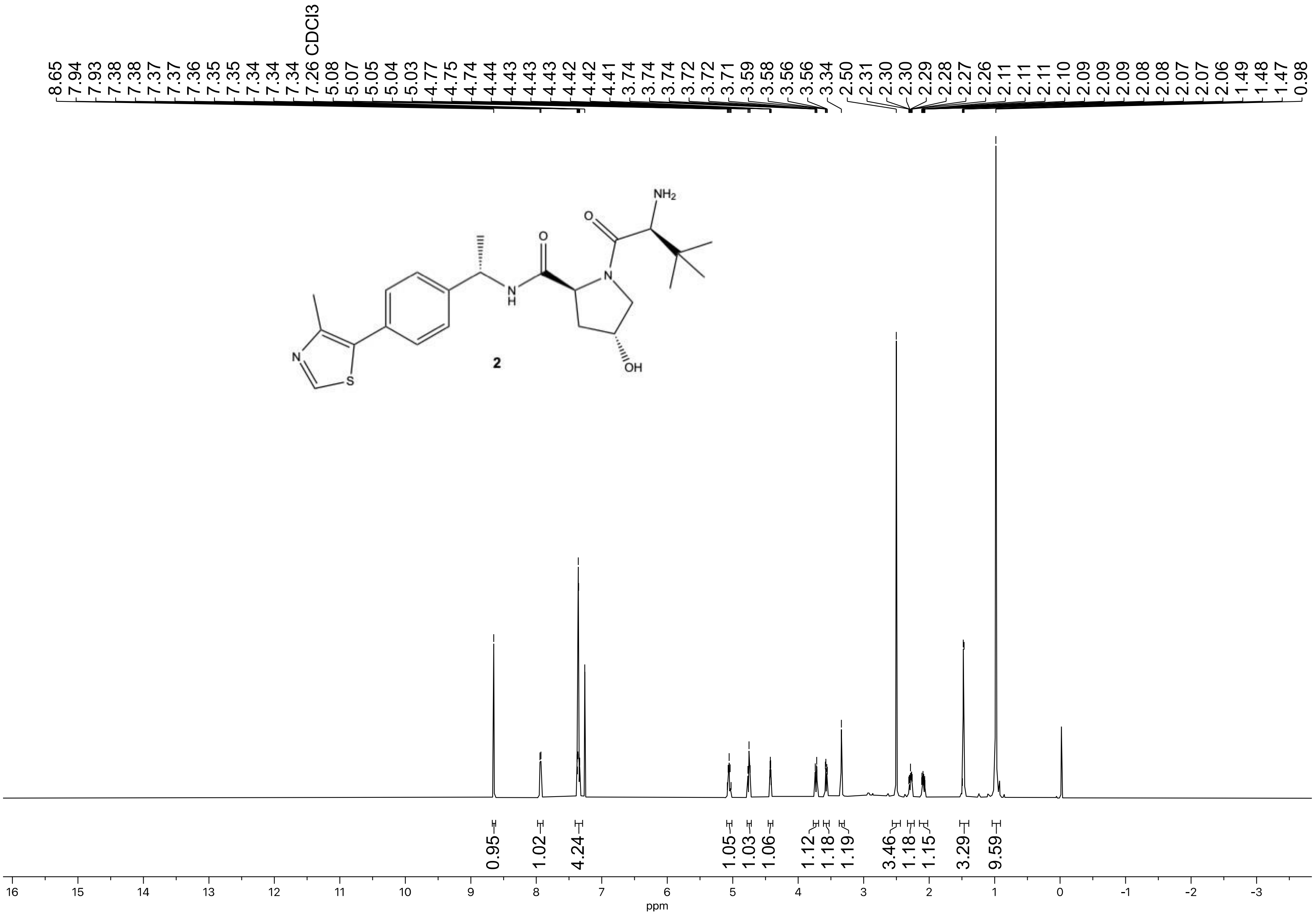

^{1}H NMR (500 MHz, $CDCl_3$) spectrum of **2**

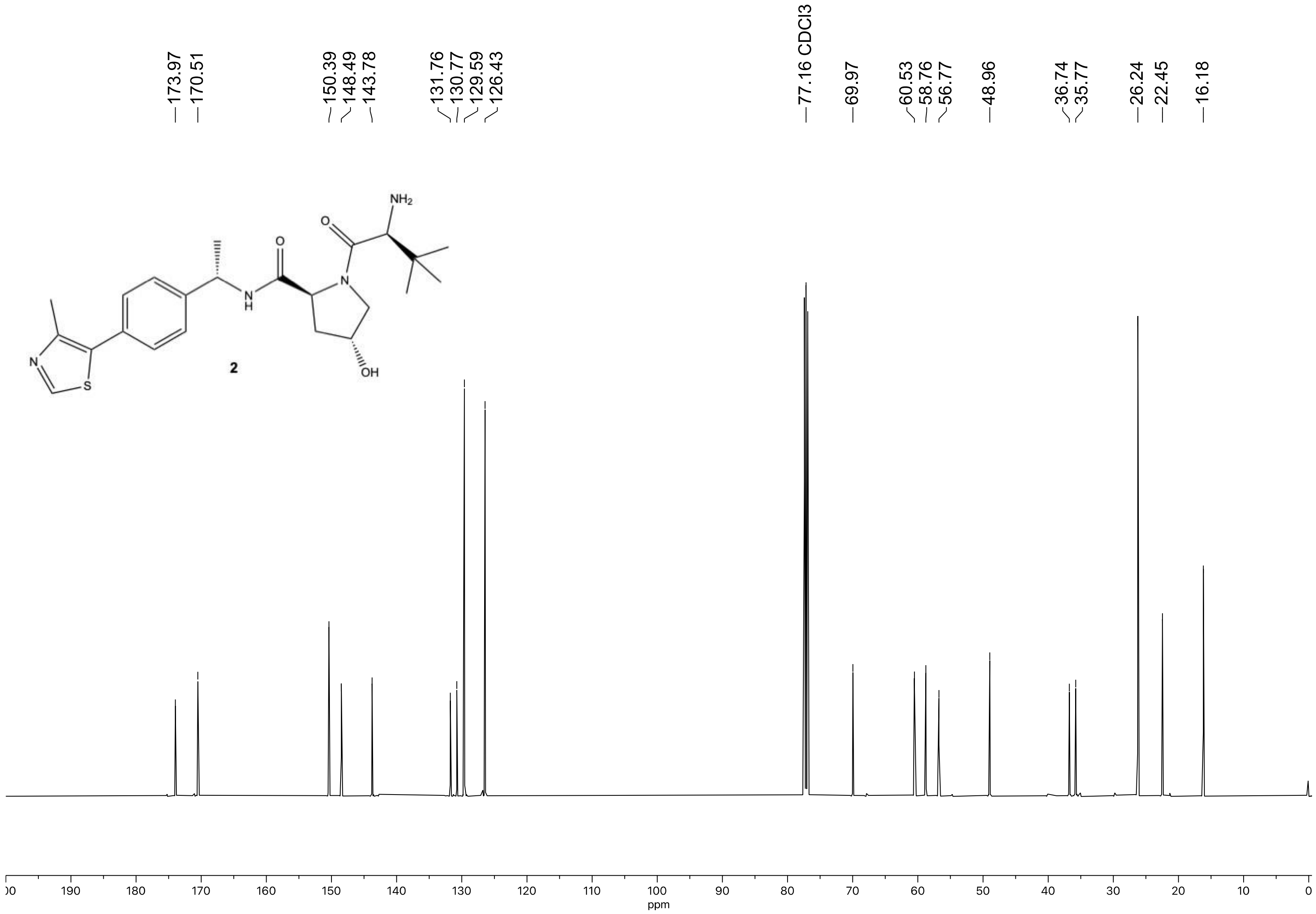

^{13}C NMR (126 MHz, $CDCl_3$) spectrum of **2**

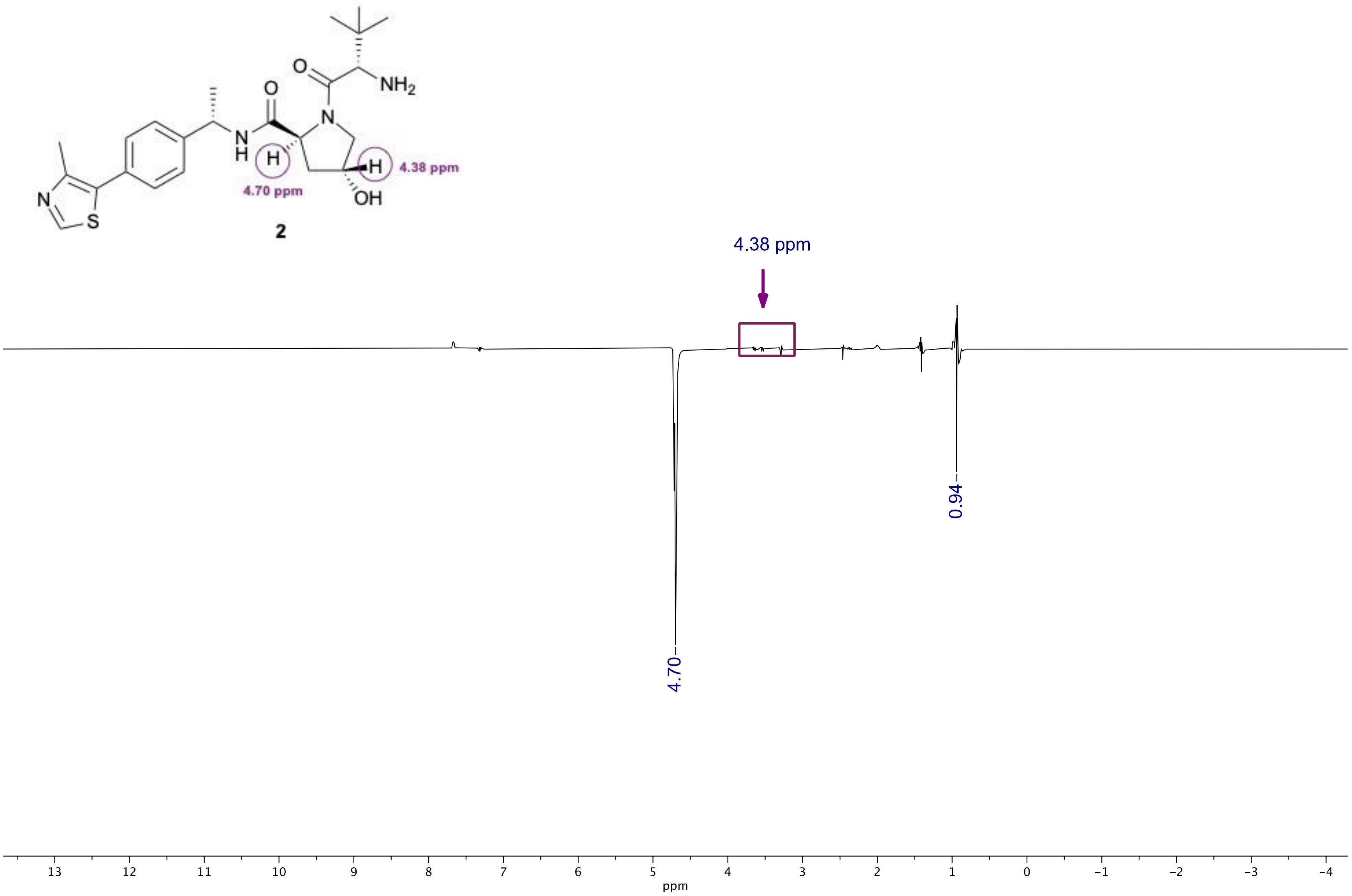

^{1}D NOE NMR (500 MHz, $CDCl_3$) spectrum of **2**

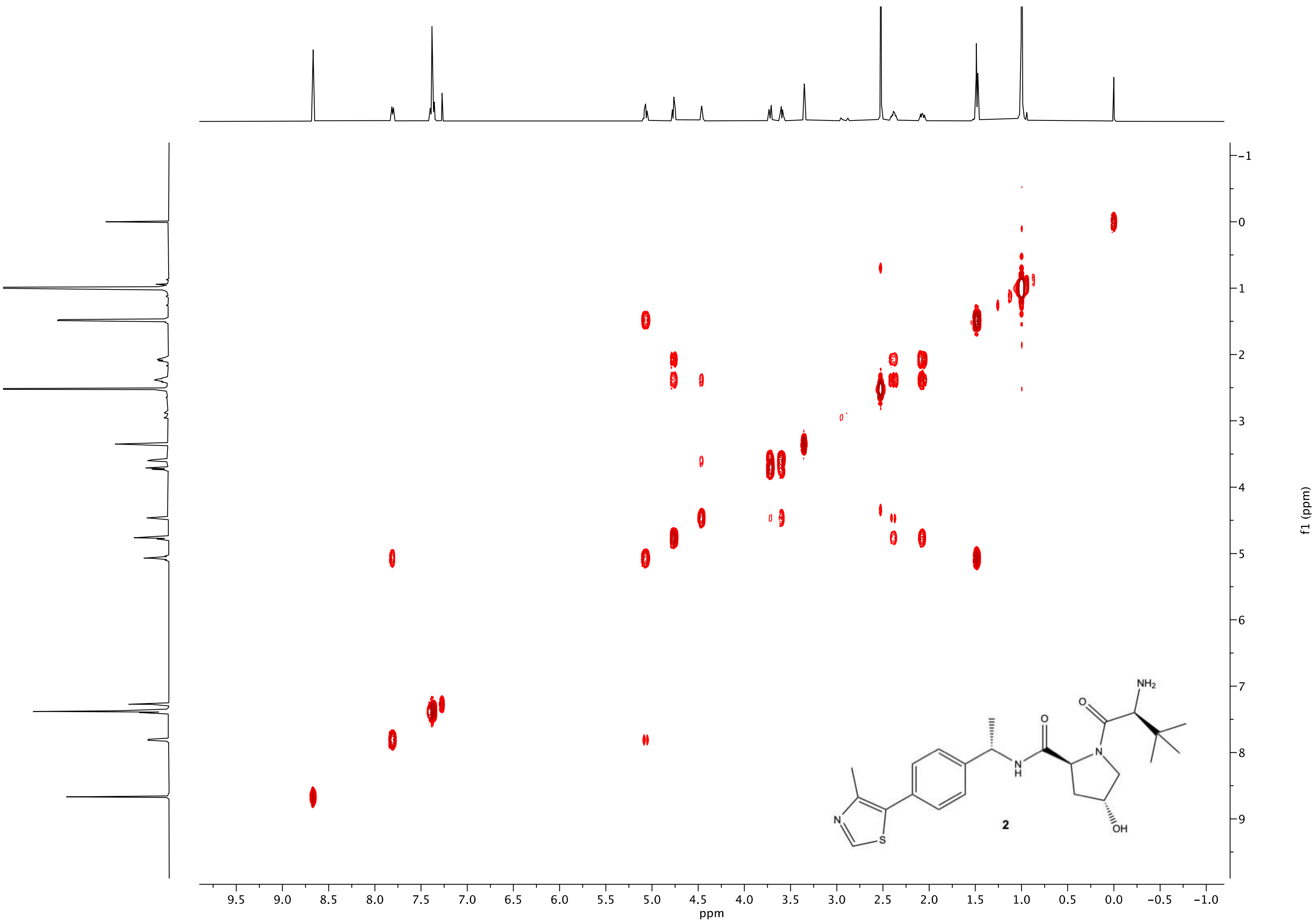

^{1}H-^{1}H COSY NMR (500 MHz, $CDCl_3$) spectrum of **2**

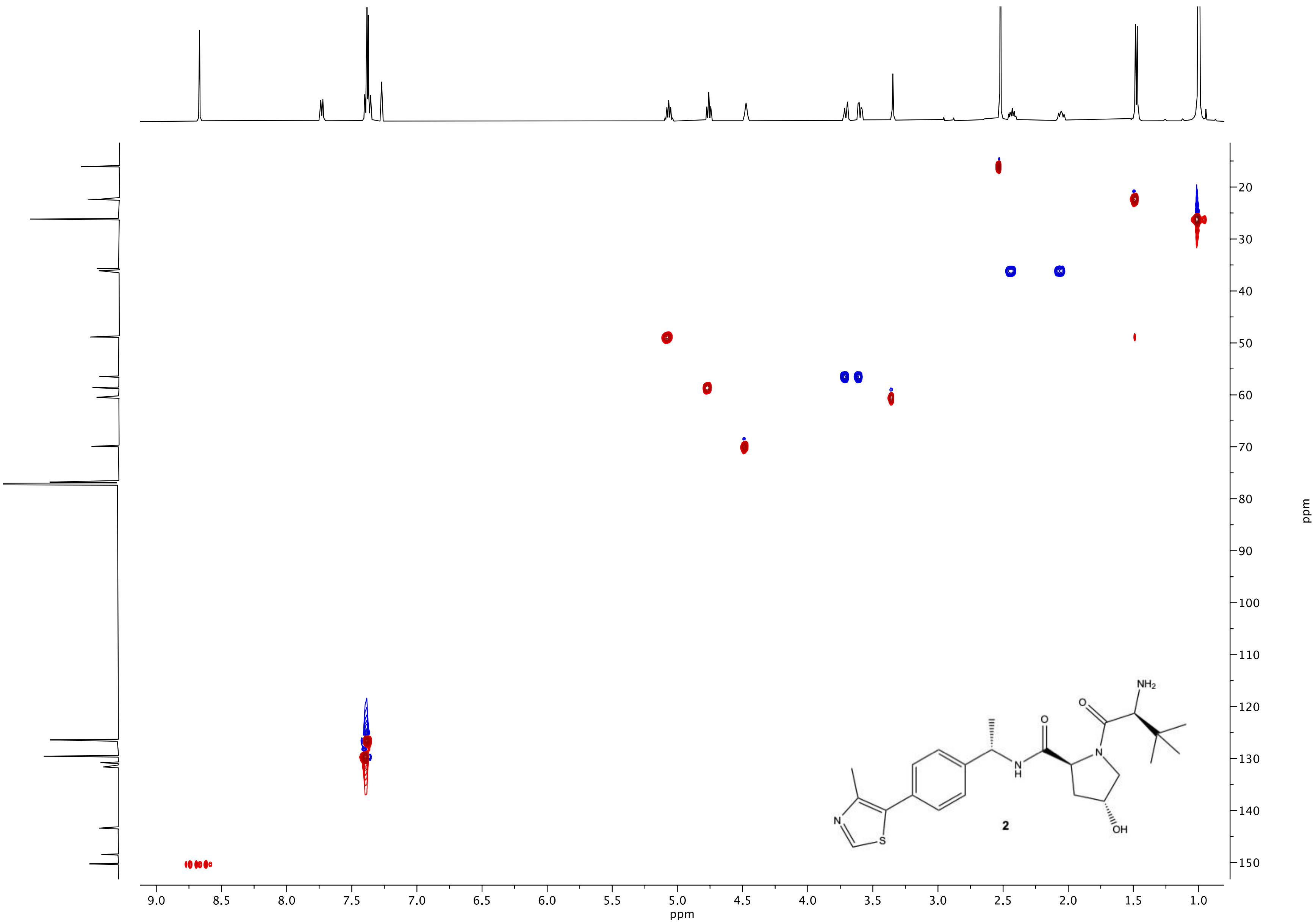

^{1}H-^{13}C HSQC NMR (500 MHz, $CDCl_3$) spectrum of **2**

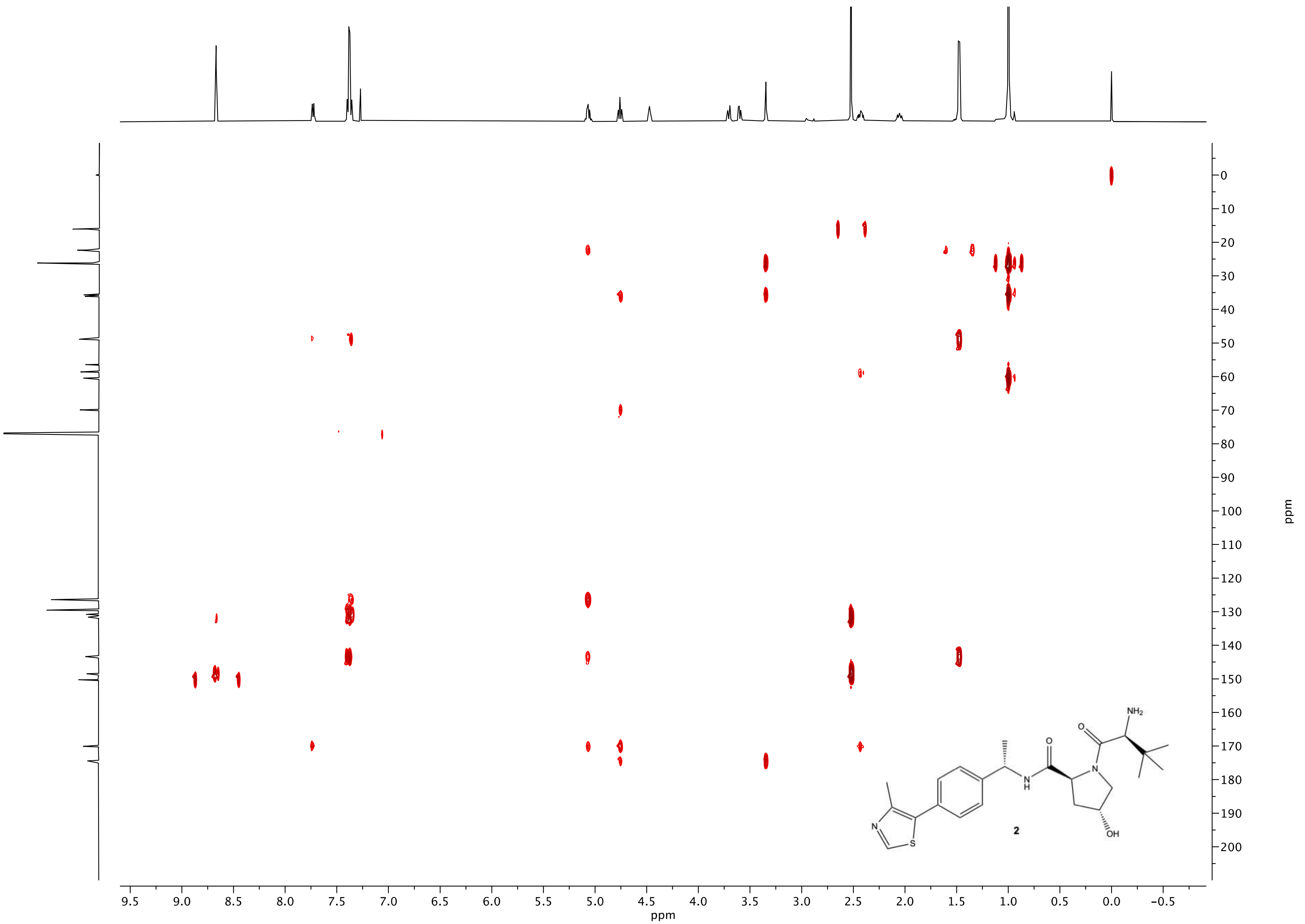

H-^{13}C HMBC NMR (500 MHz, $CDCl_3$) spectrum of **2**

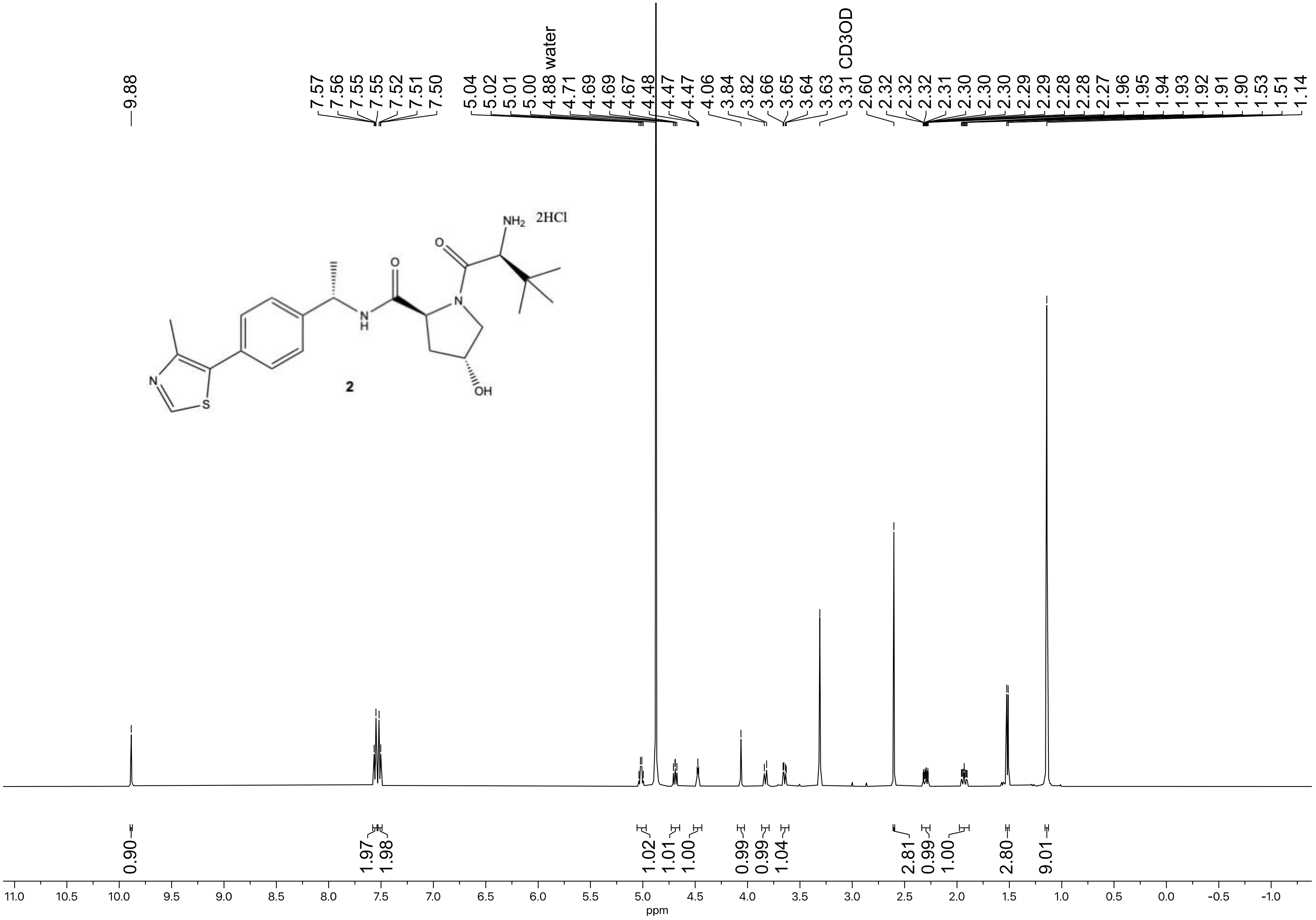

^{1}H NMR (500 MHz, CD_3OD) spectrum of **2** (hydrochloride salt)

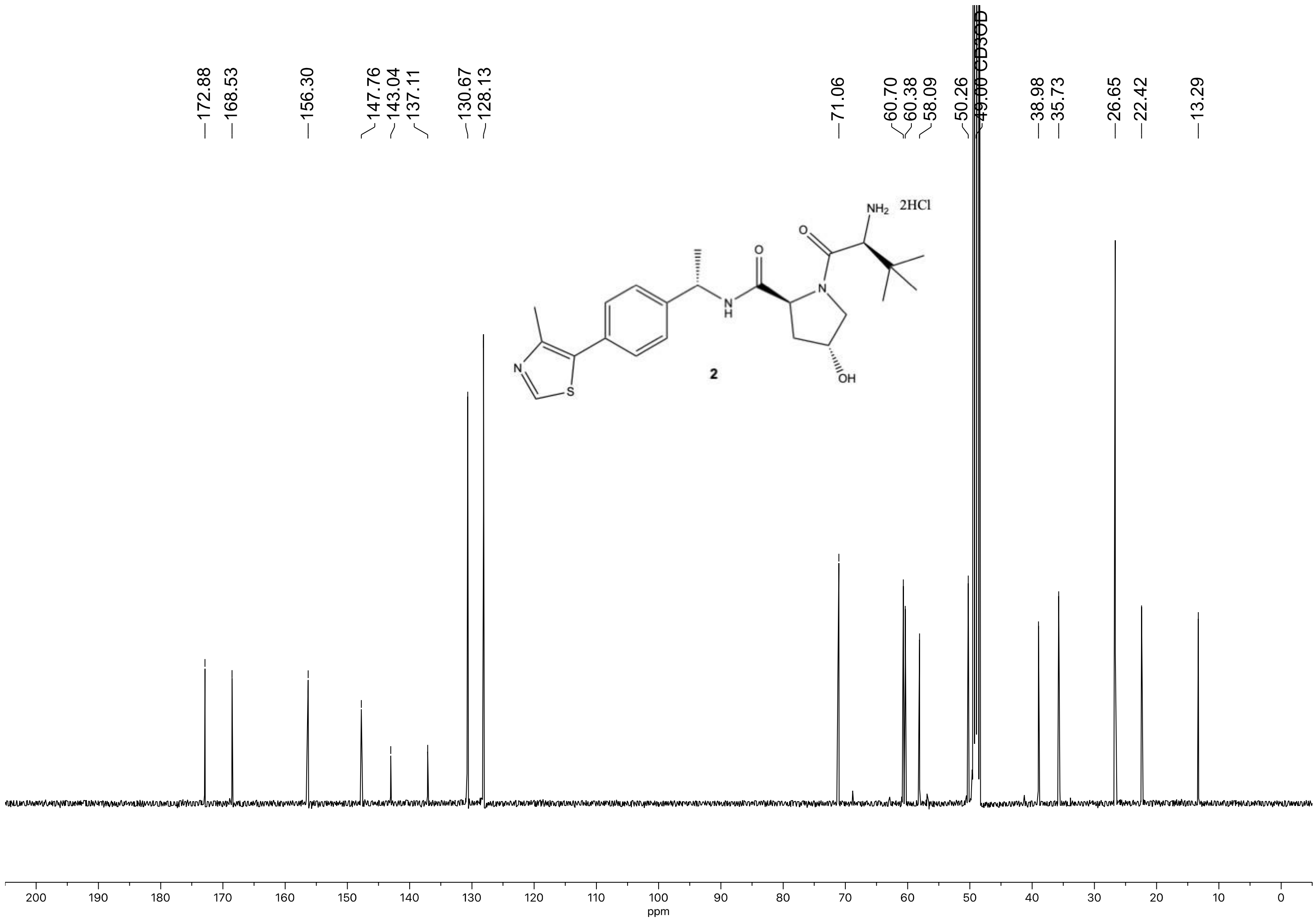

^{13}C NMR (125 MHz, CD_3OD) spectrum of **2** (hydrochloride salt)

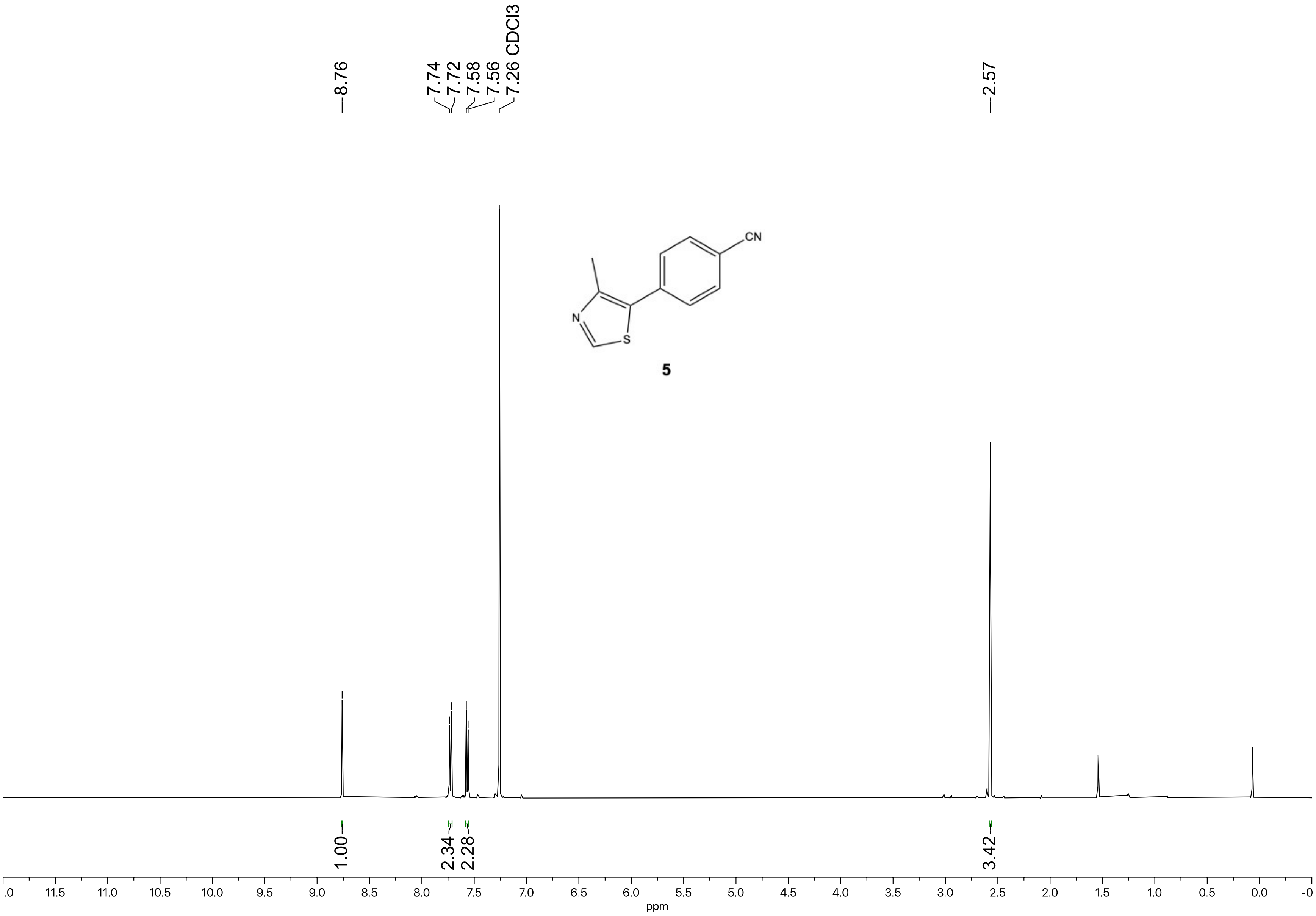

^{1}H NMR (500 MHz, $CDCl_3$) spectrum of **5**

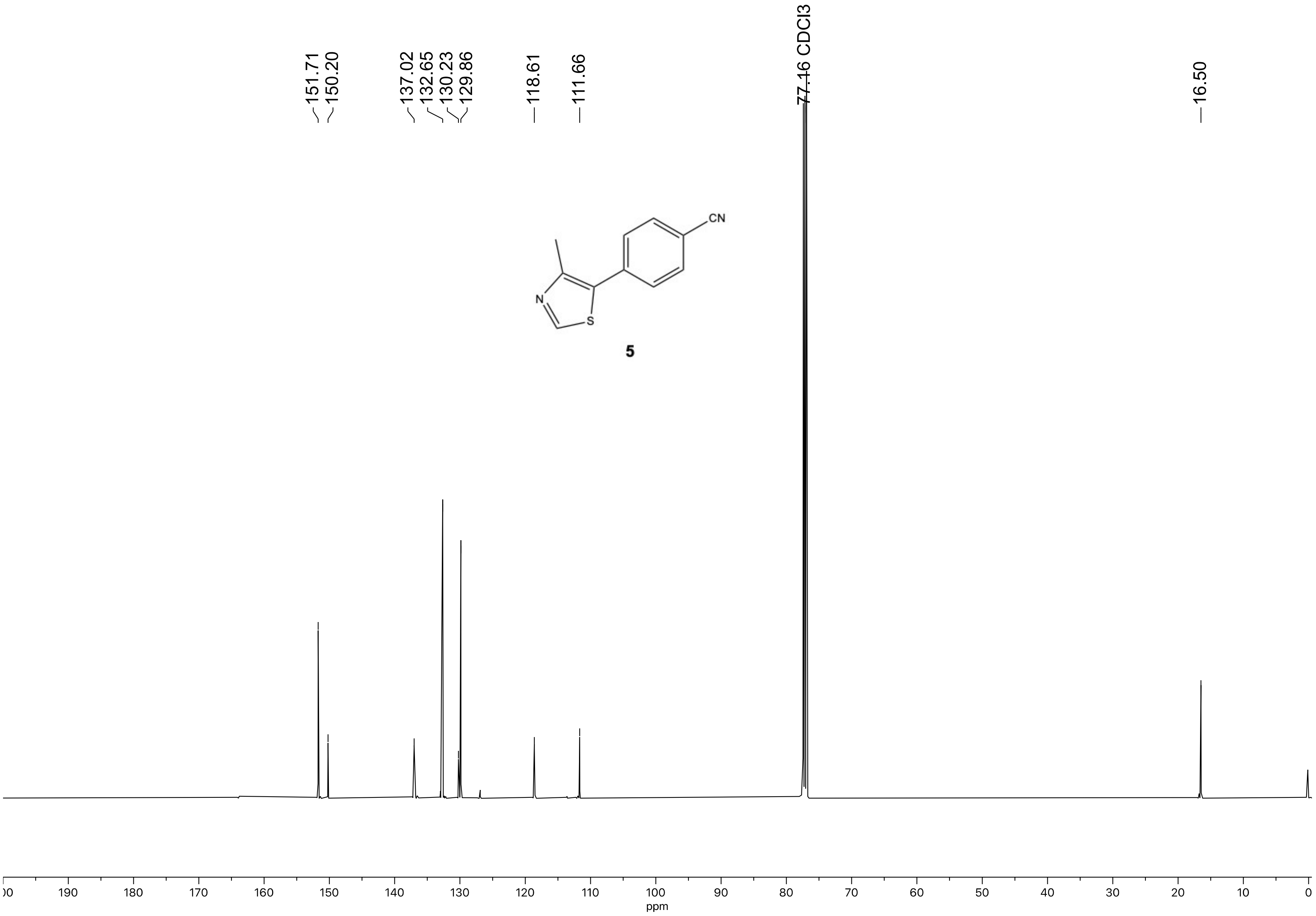

^{13}C NMR (126 MHz, $CDCl_3$) spectrum of **5**

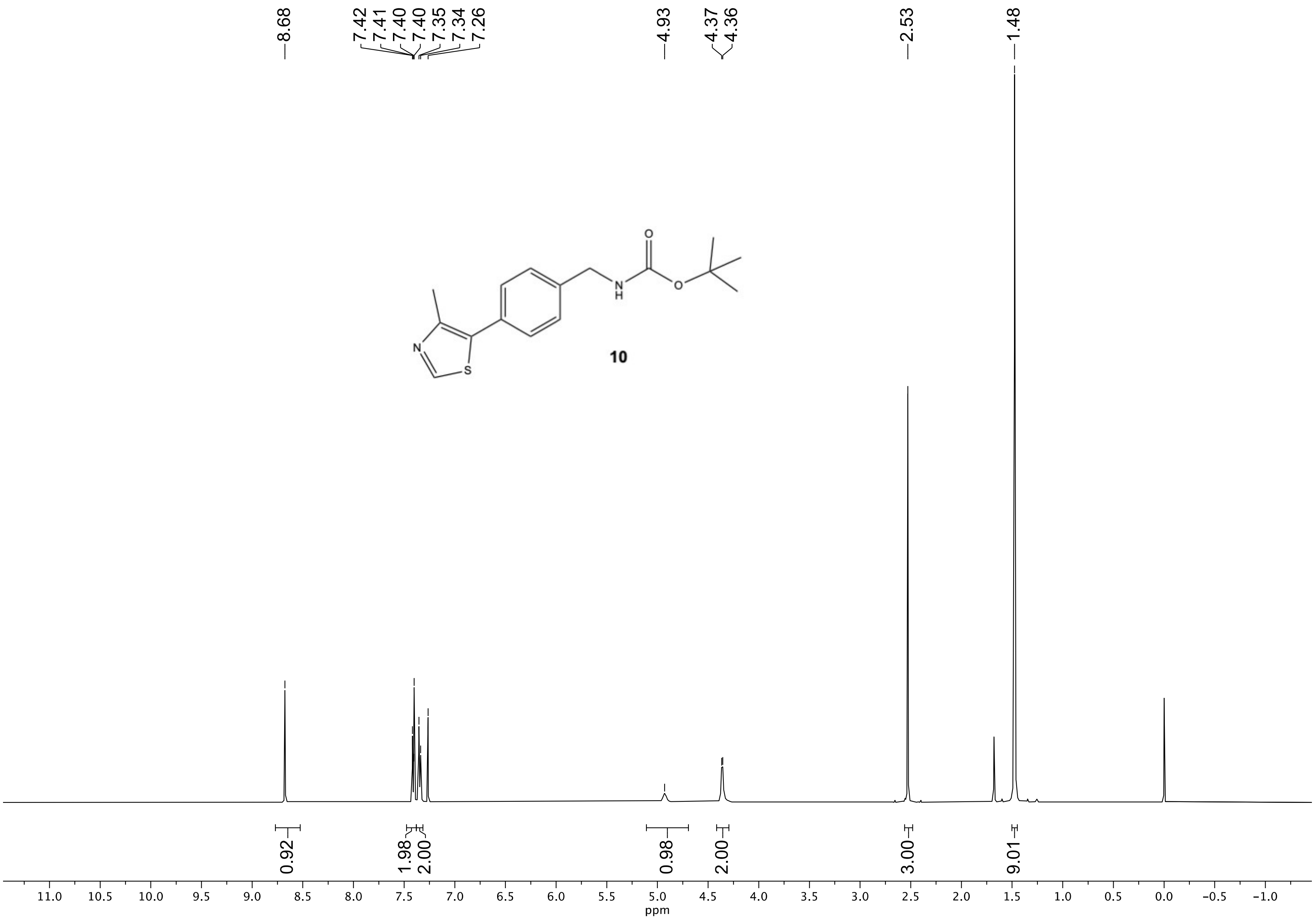

^{1}H NMR (500 MHz, $CDCl_3$) spectrum of **10**

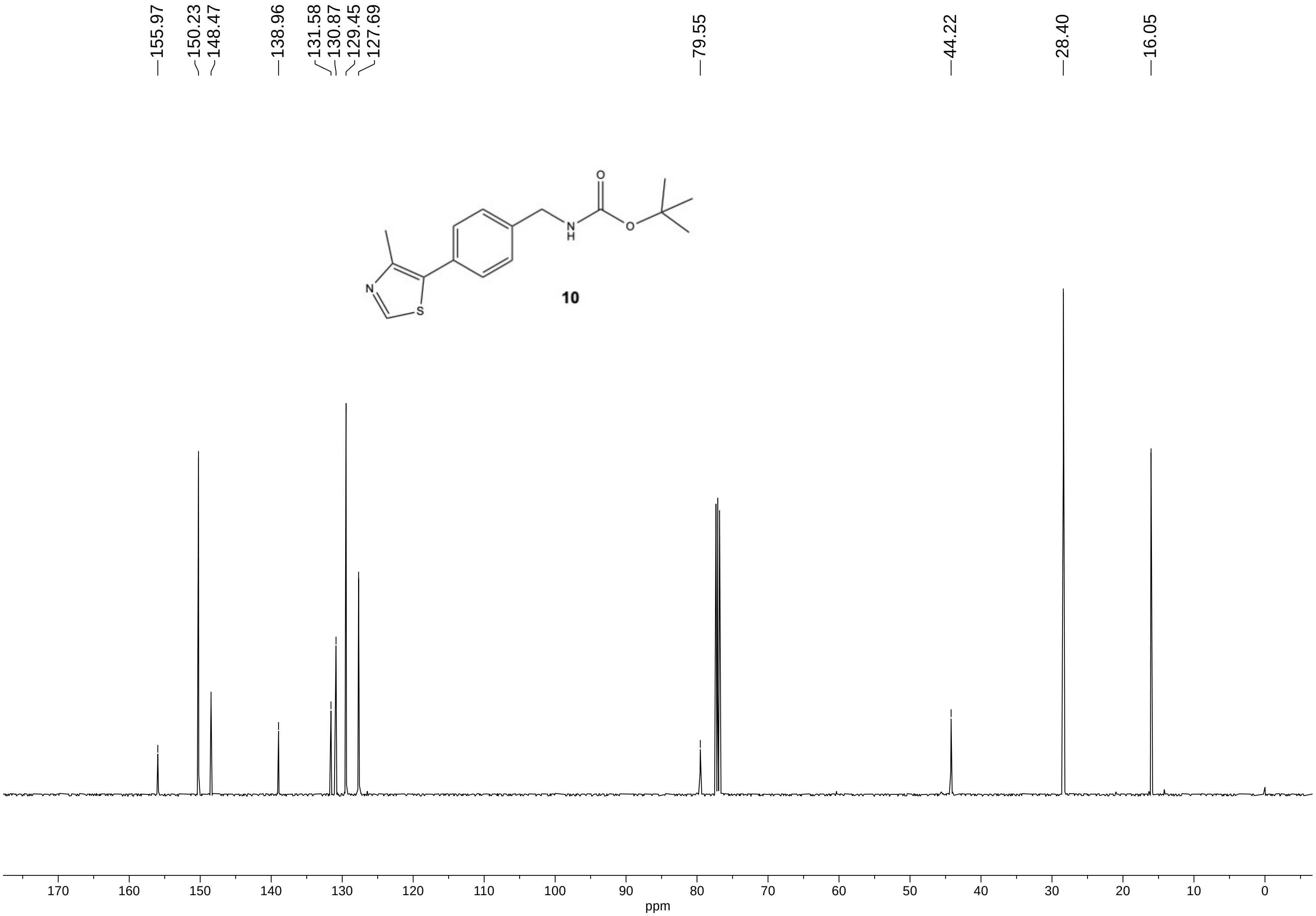

^{13}C NMR (126 MHz, $CDCl_3$) spectrum of **10**

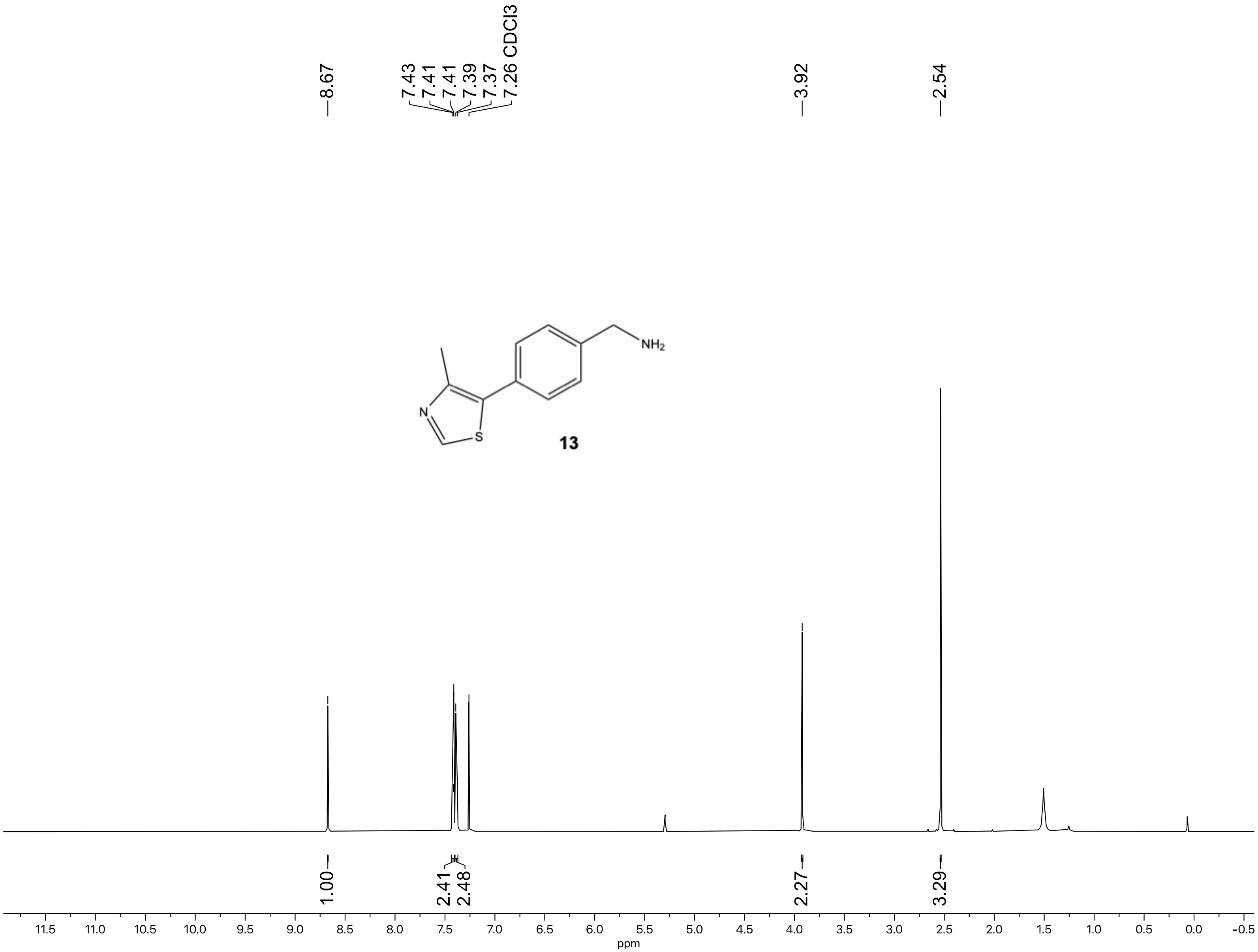

^{1}H NMR (500 MHz, $CDCl_3$) spectrum of **13**

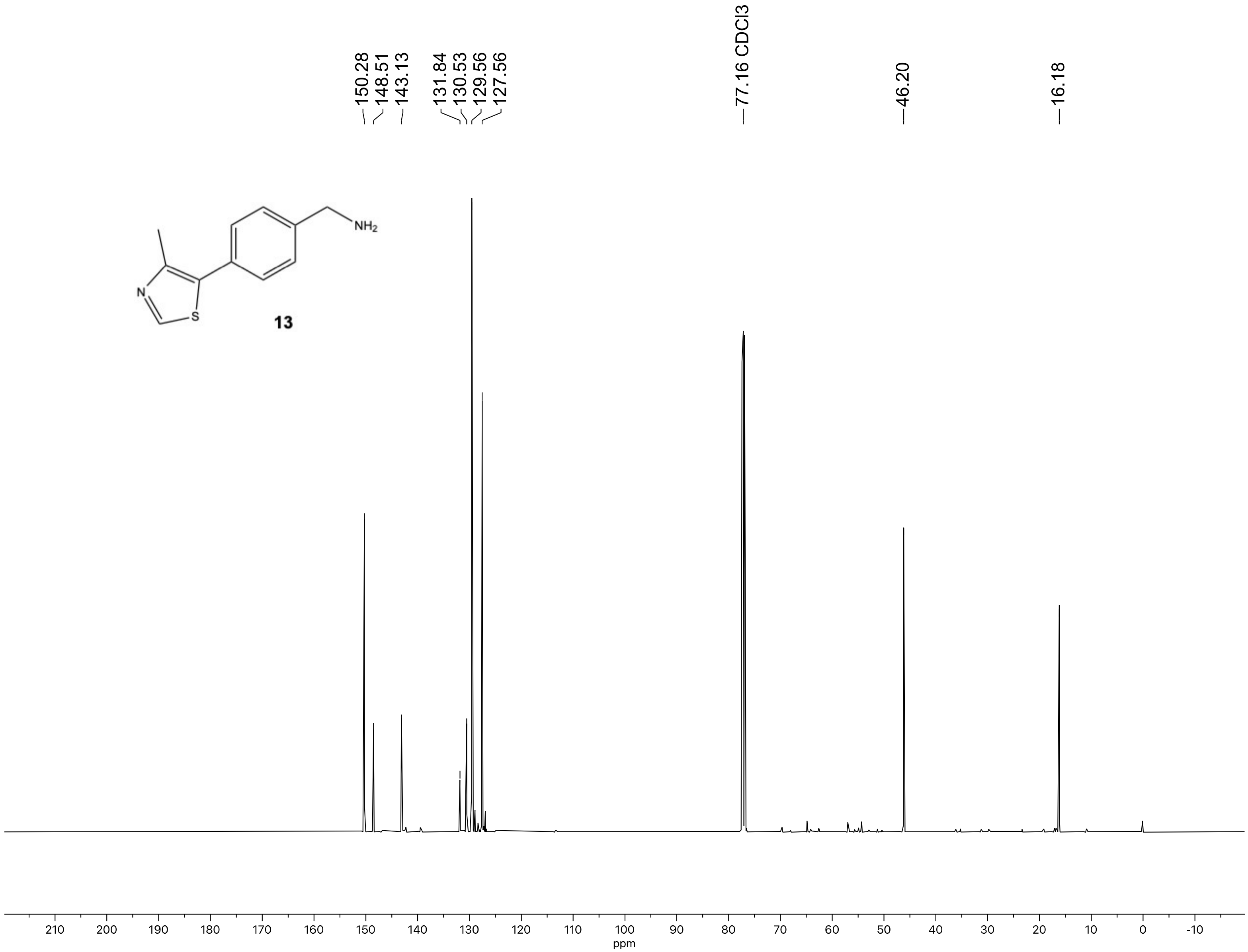

^{13}C NMR (126 MHz, CDCl$_3$) spectrum of **13**

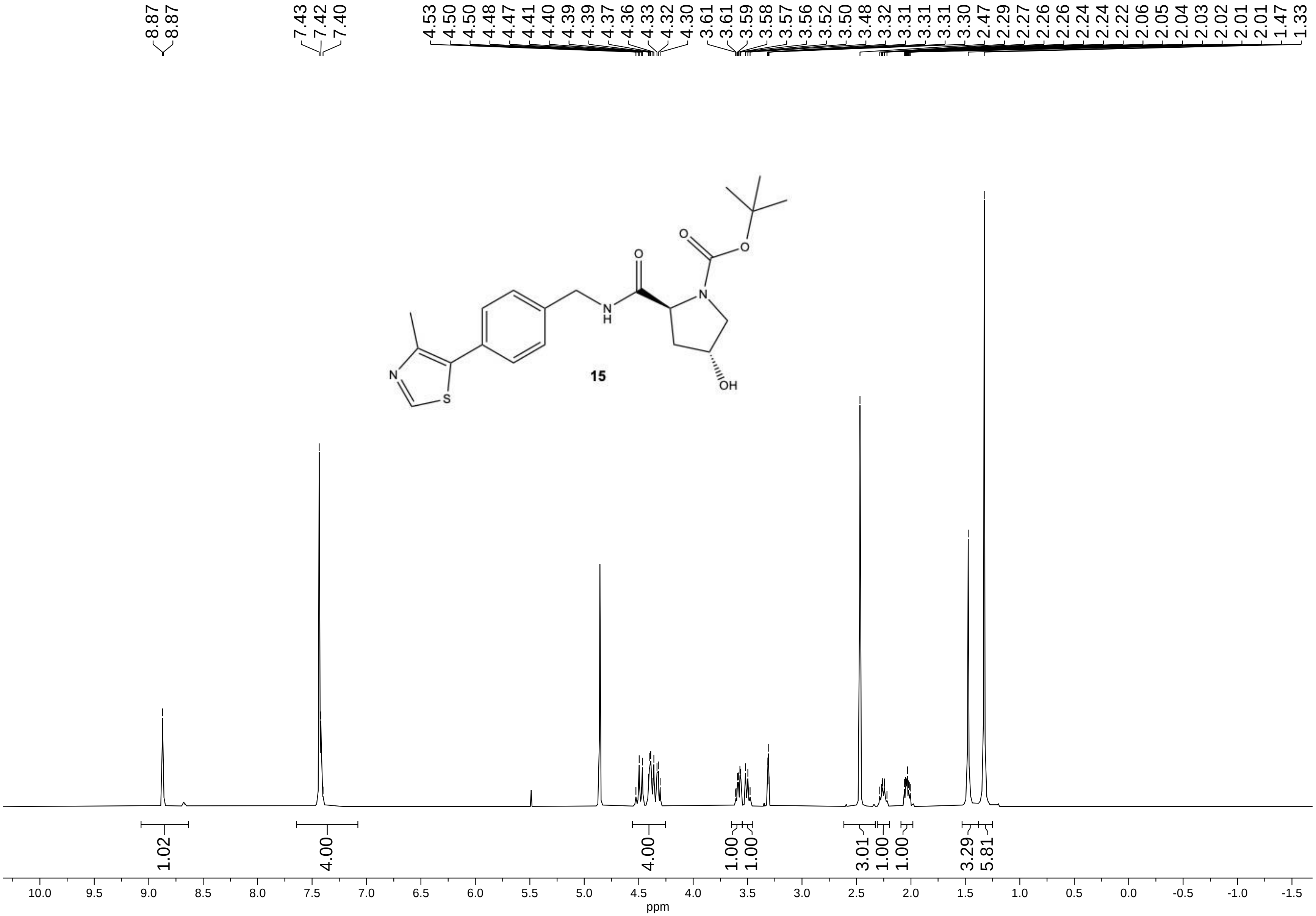

^{1}H NMR (500 MHz, $(CD_3)_2CO$) spectrum of **15**

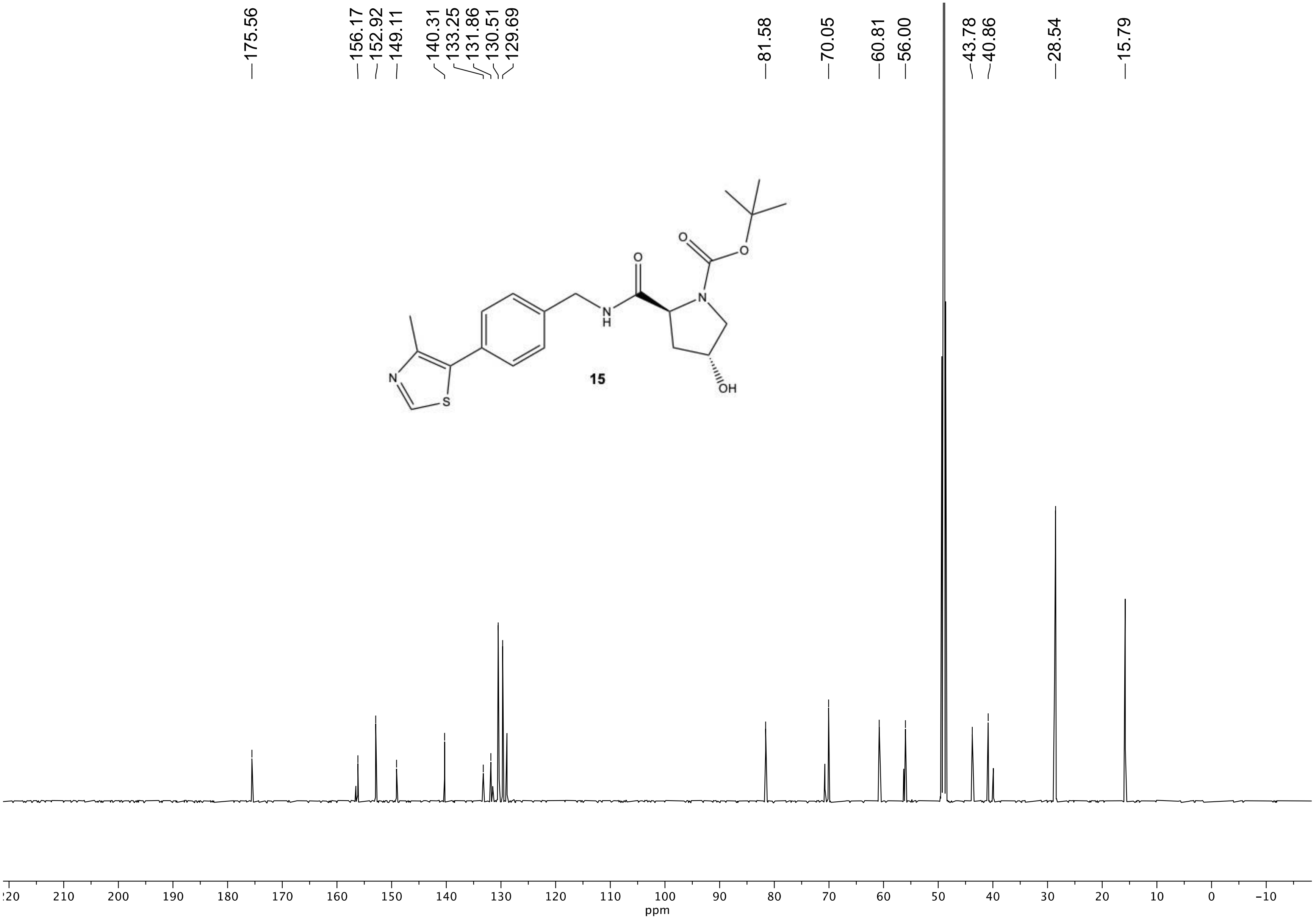

^{13}C NMR (126 MHz, $(CD_3)_2CO$) spectrum of **15**

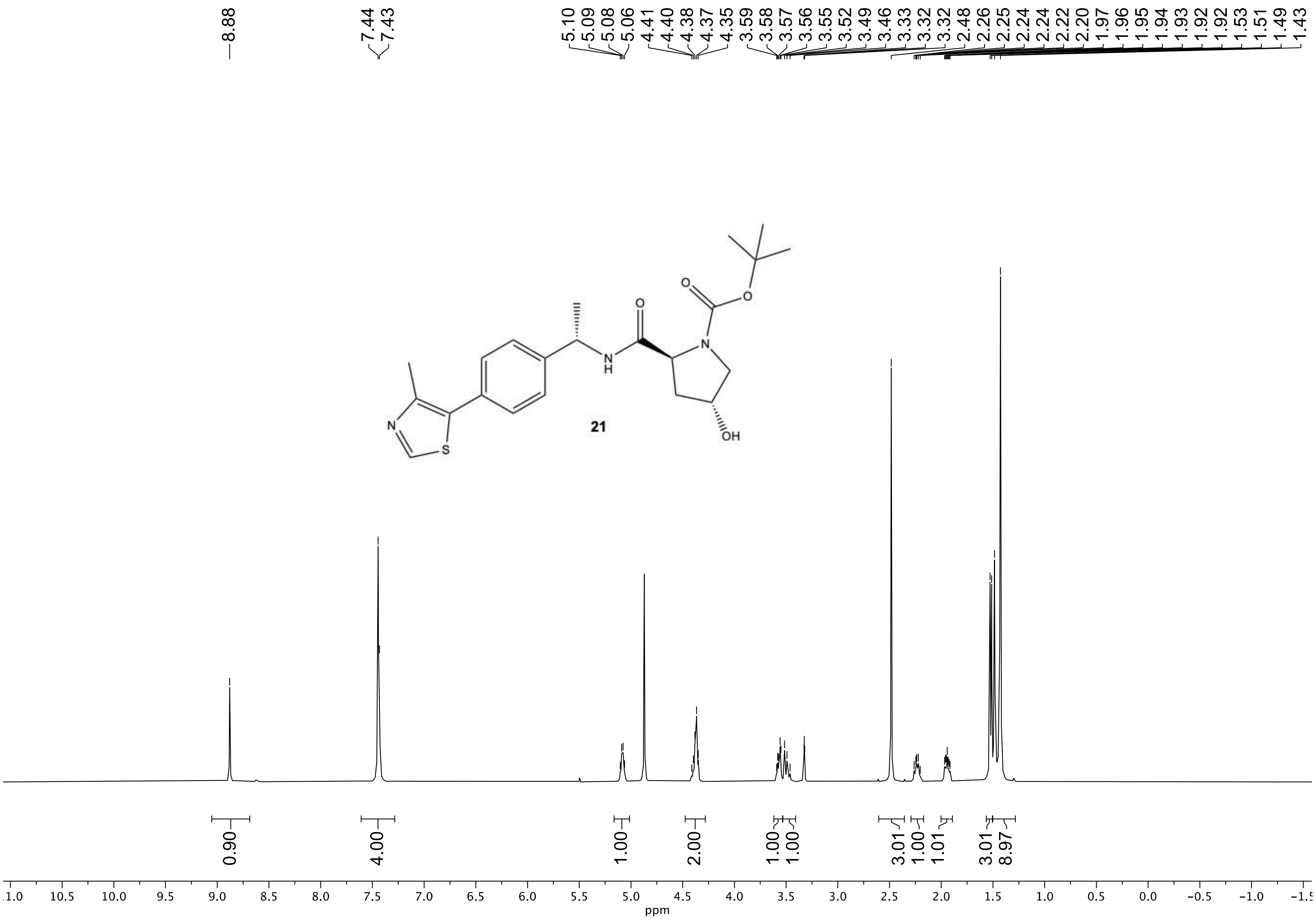

^{1}H NMR (500 MHz, $(CD_3)_2CO$) spectrum of **21**

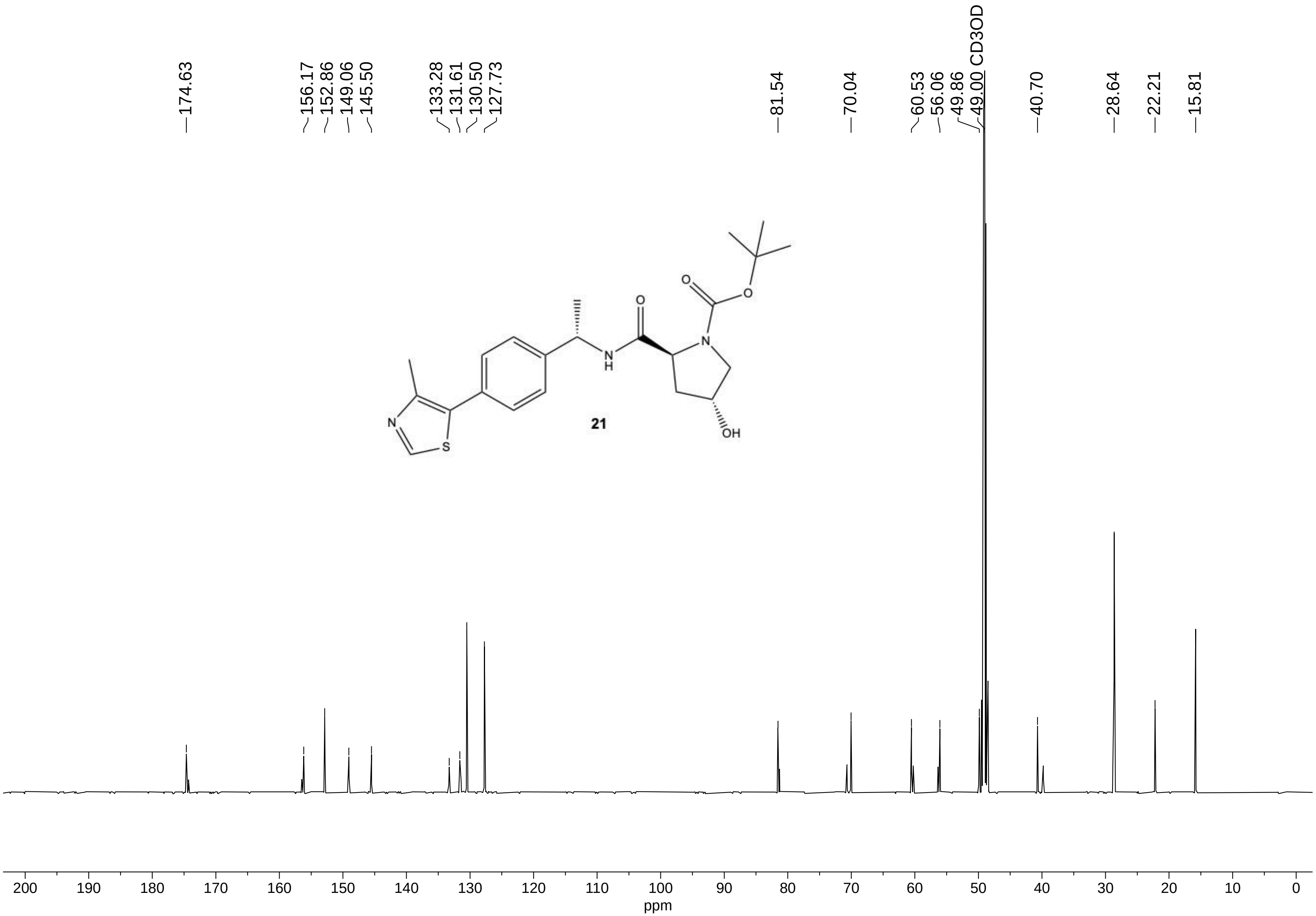

^{13}C NMR (126 MHz, $(CD_3)_2CO$) spectrum of **21**

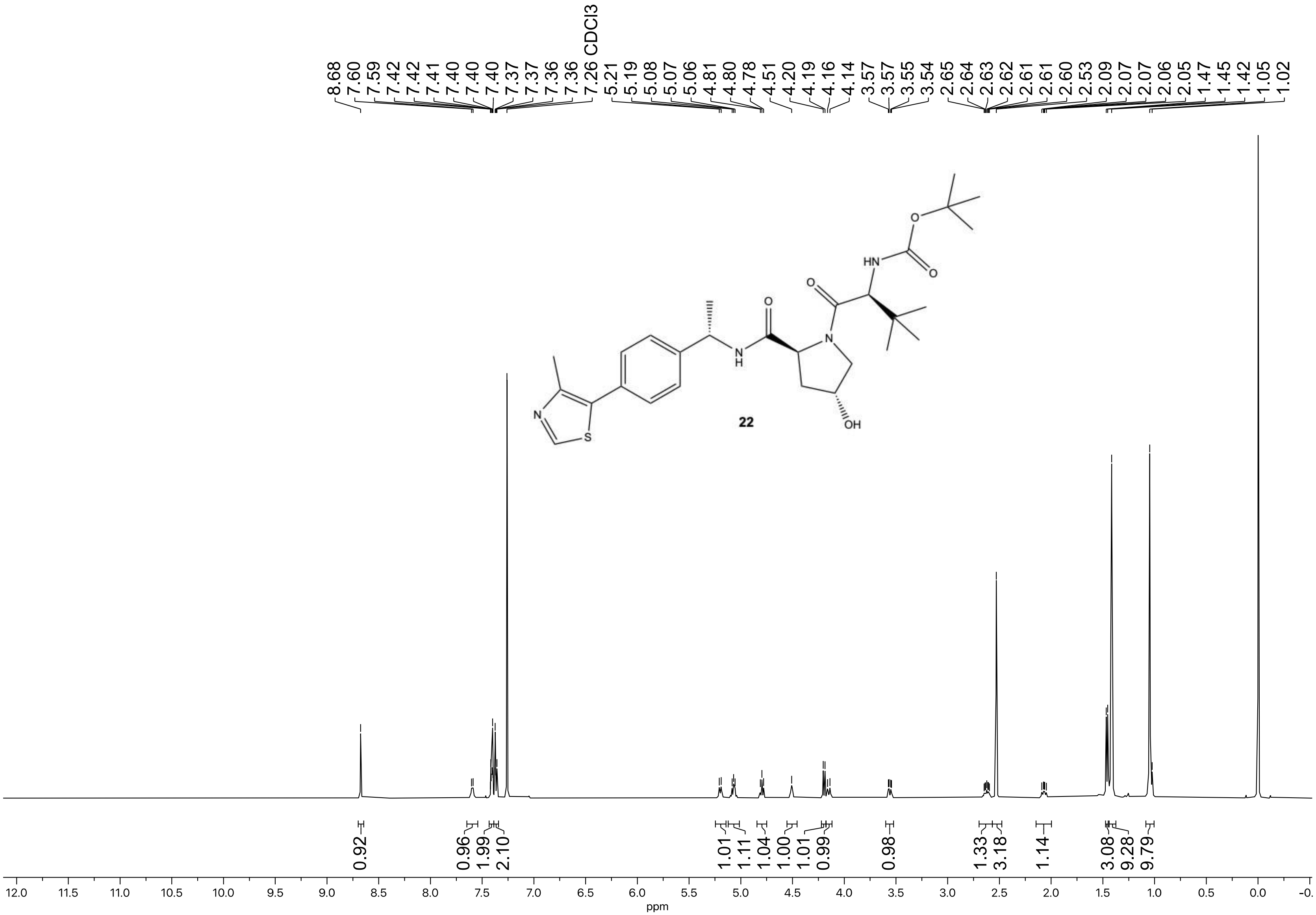

^{1}H NMR (500 MHz, $CDCl_3$) spectrum of **22**

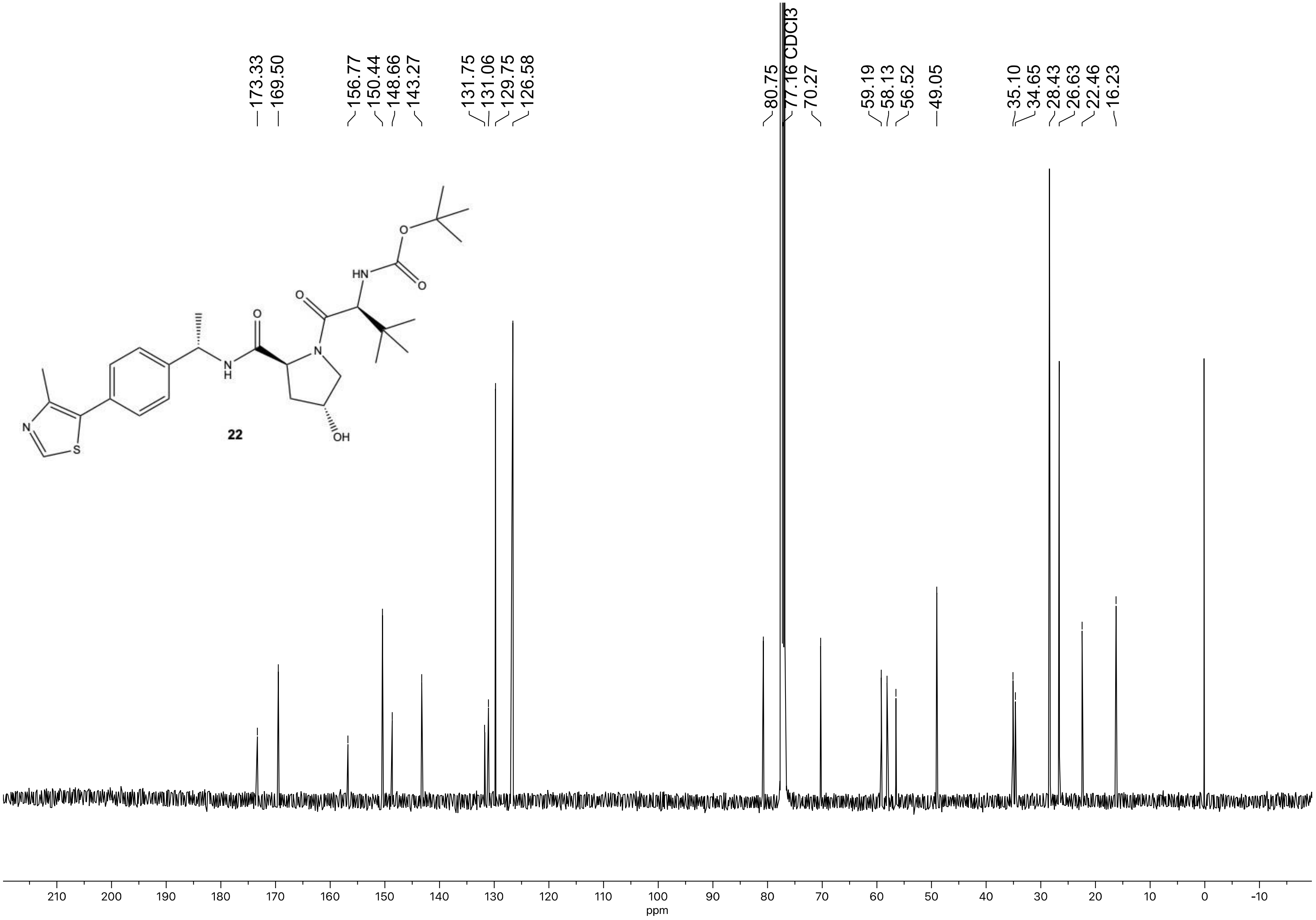

^{13}C NMR (126 MHz, $CDCl_3$) spectrum of **22**

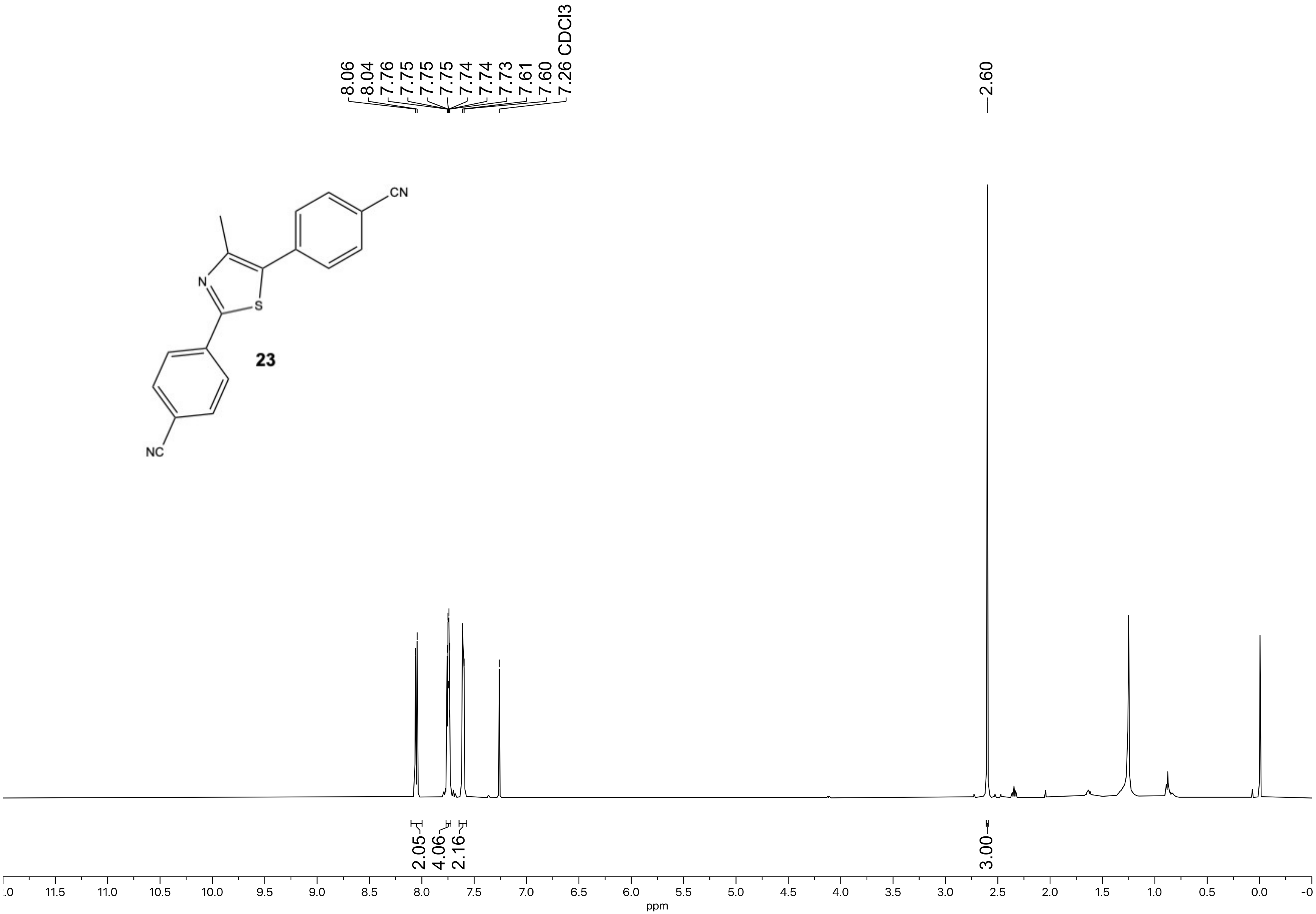

^{1}H NMR (500 MHz, $CDCl_3$) spectrum of **23**

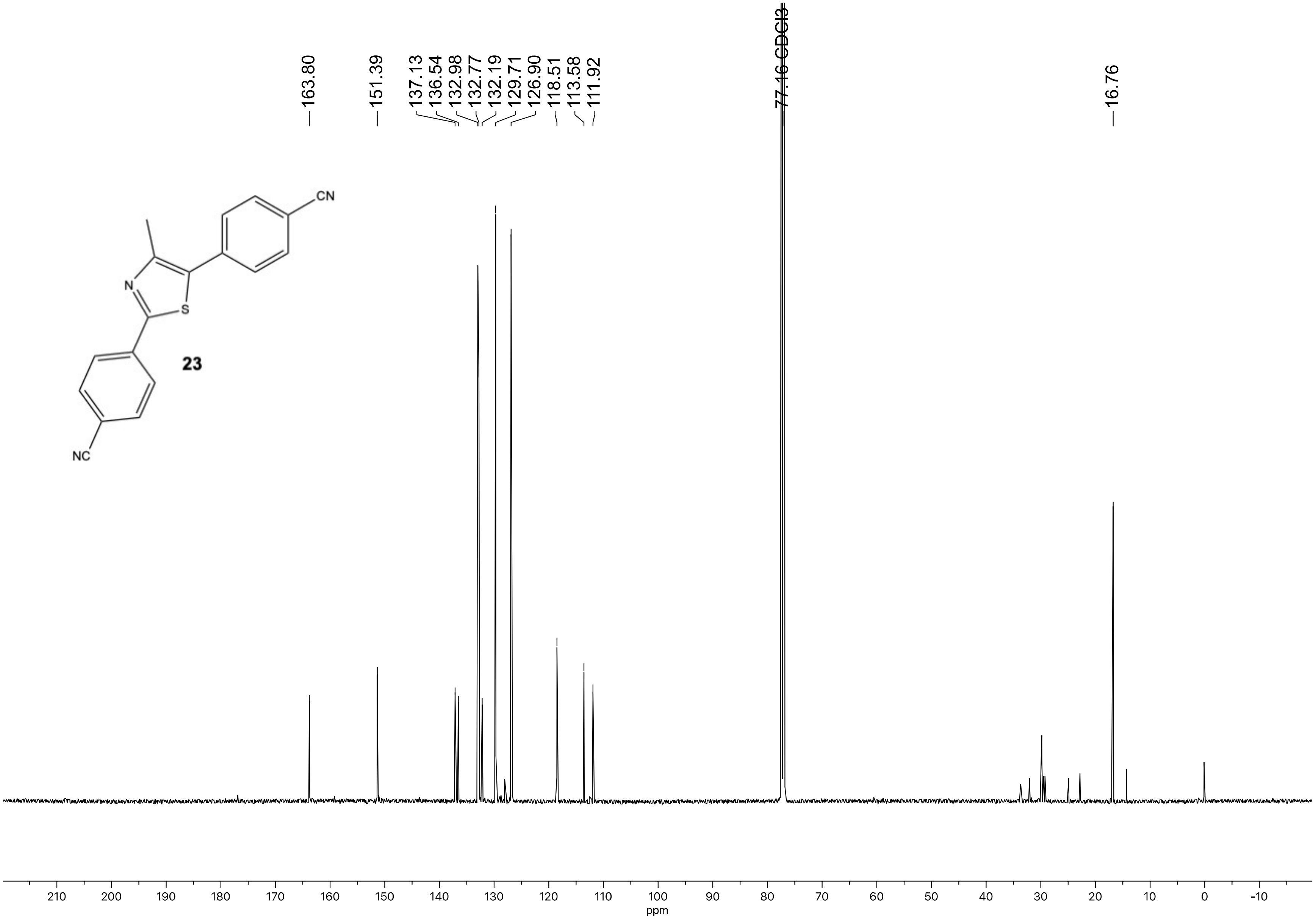

^{13}C NMR (126 MHz, $CDCl_3$) spectrum of **23**

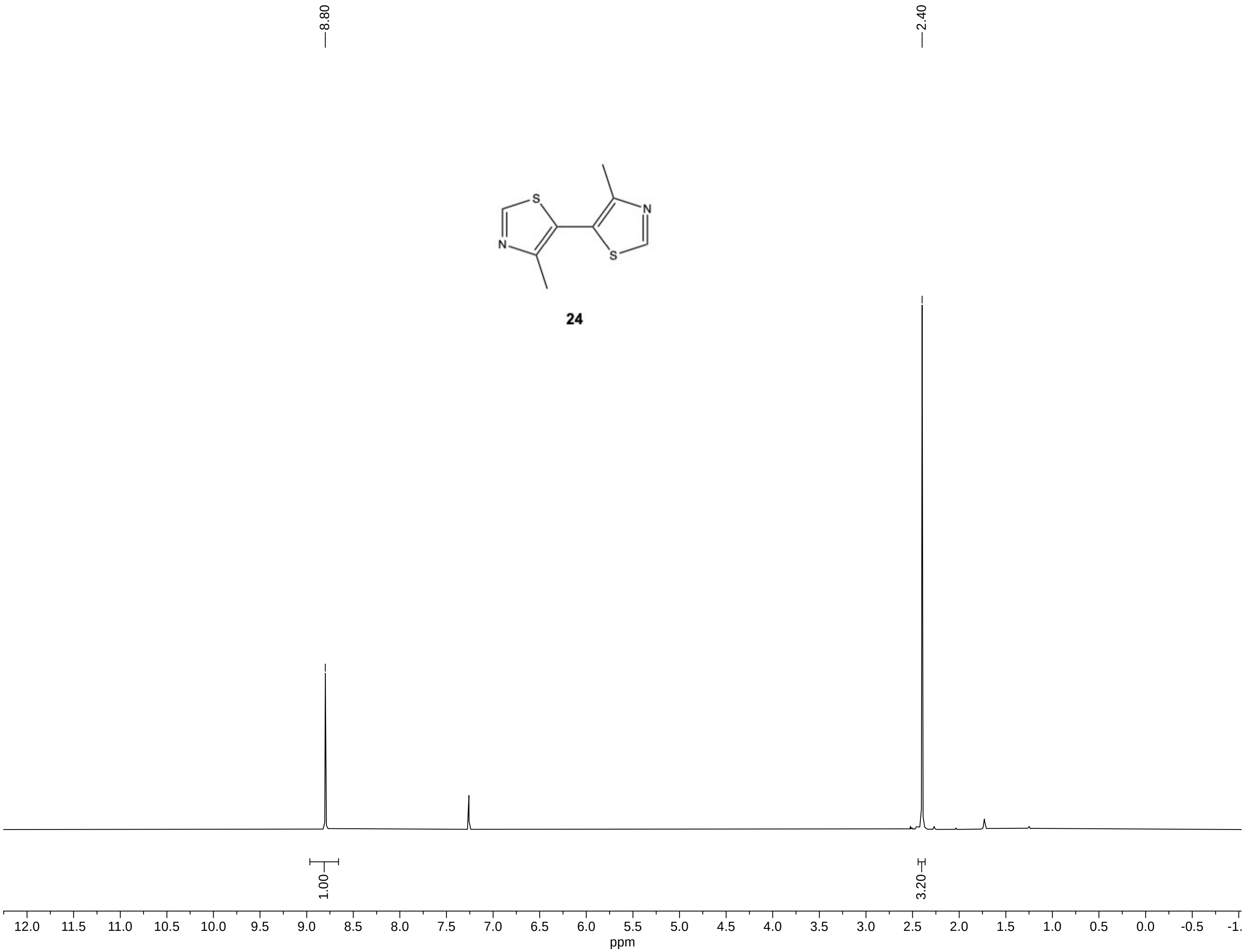

^{1}H NMR (500 MHz, $CDCl_3$) spectrum of **24**

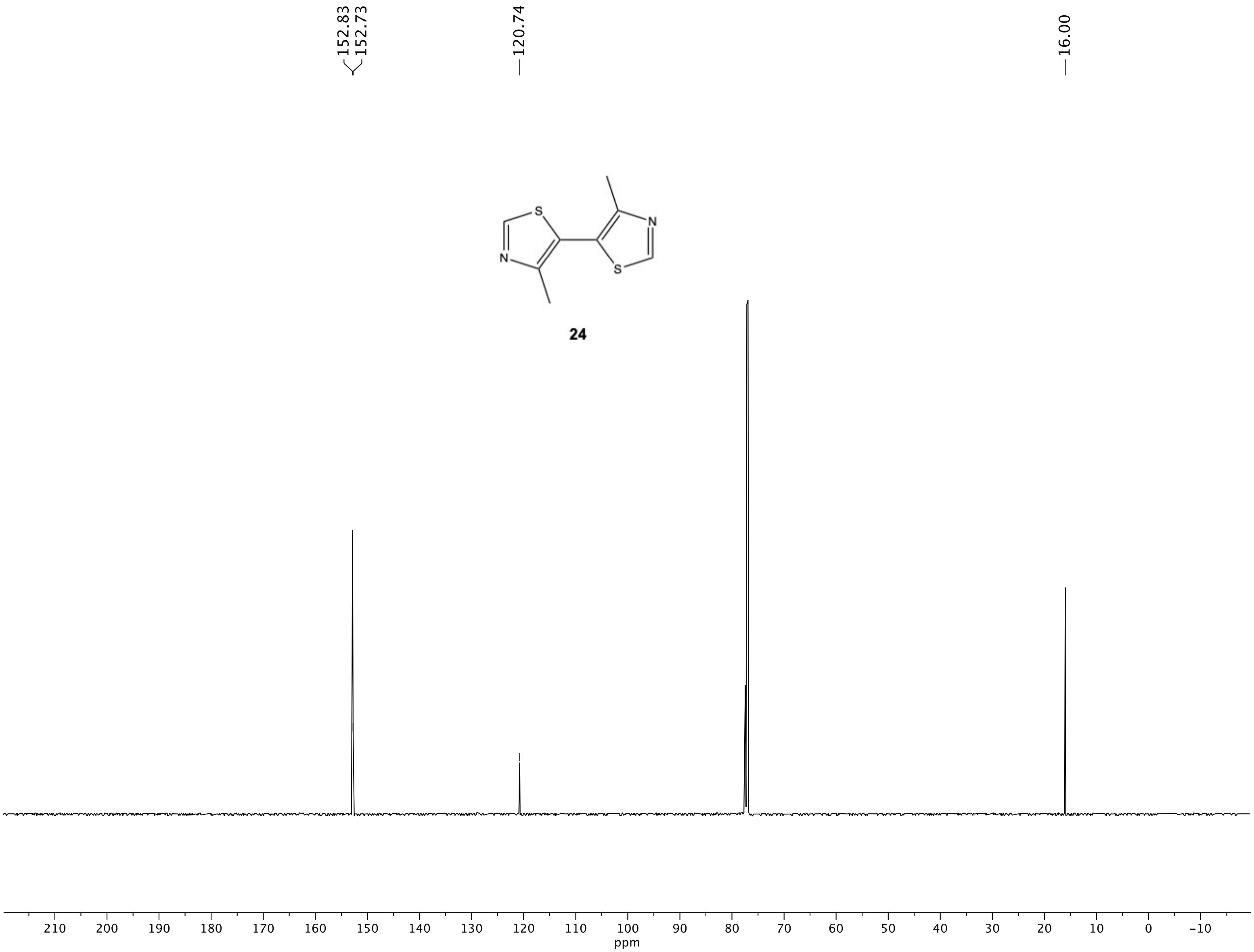

^{13}C NMR (126 MHz, $CDCl_3$) spectrum of **24**

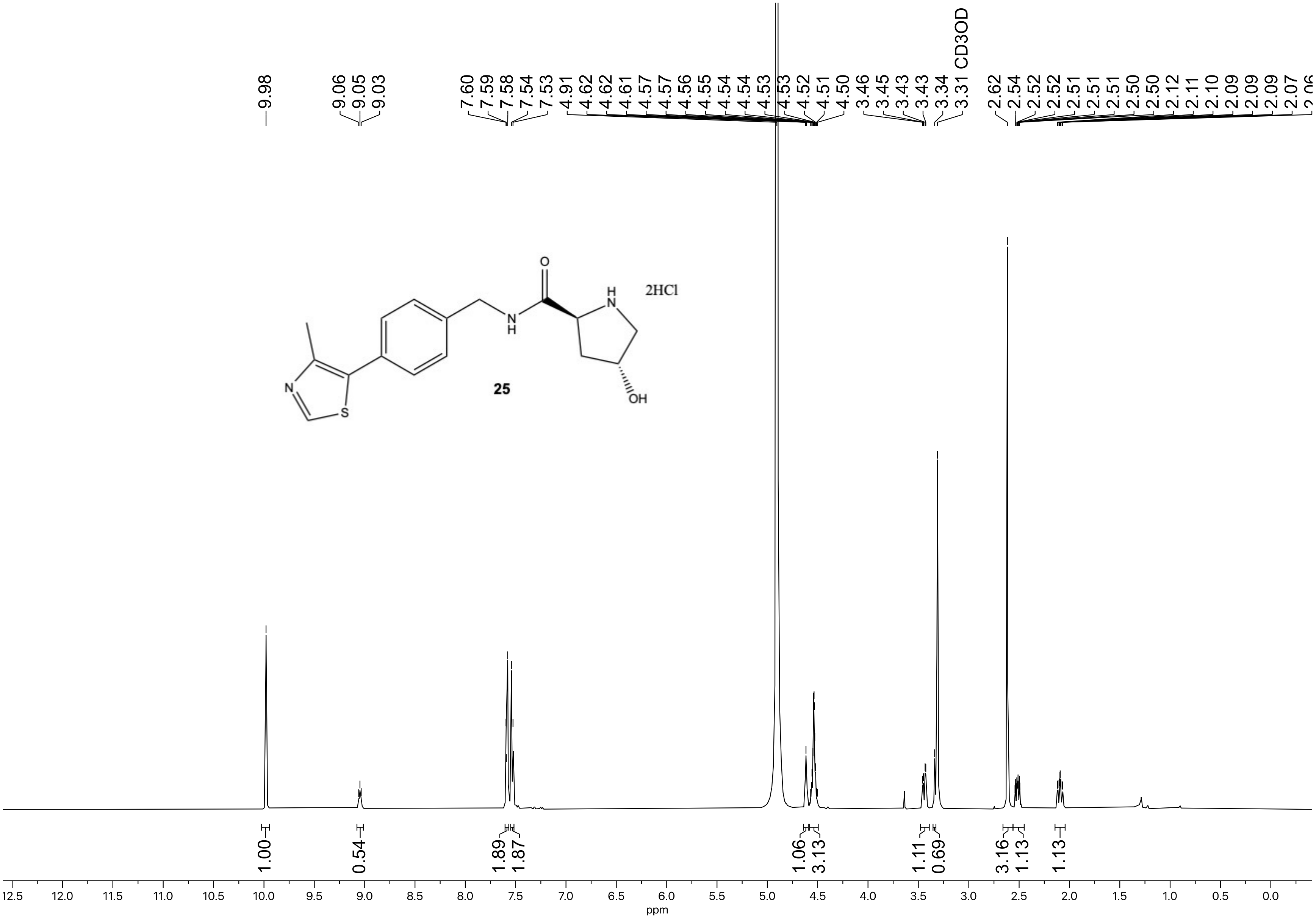

^{1}H NMR (500 MHz, CD_3OD) spectrum of **25** (Hydrochloride salt)

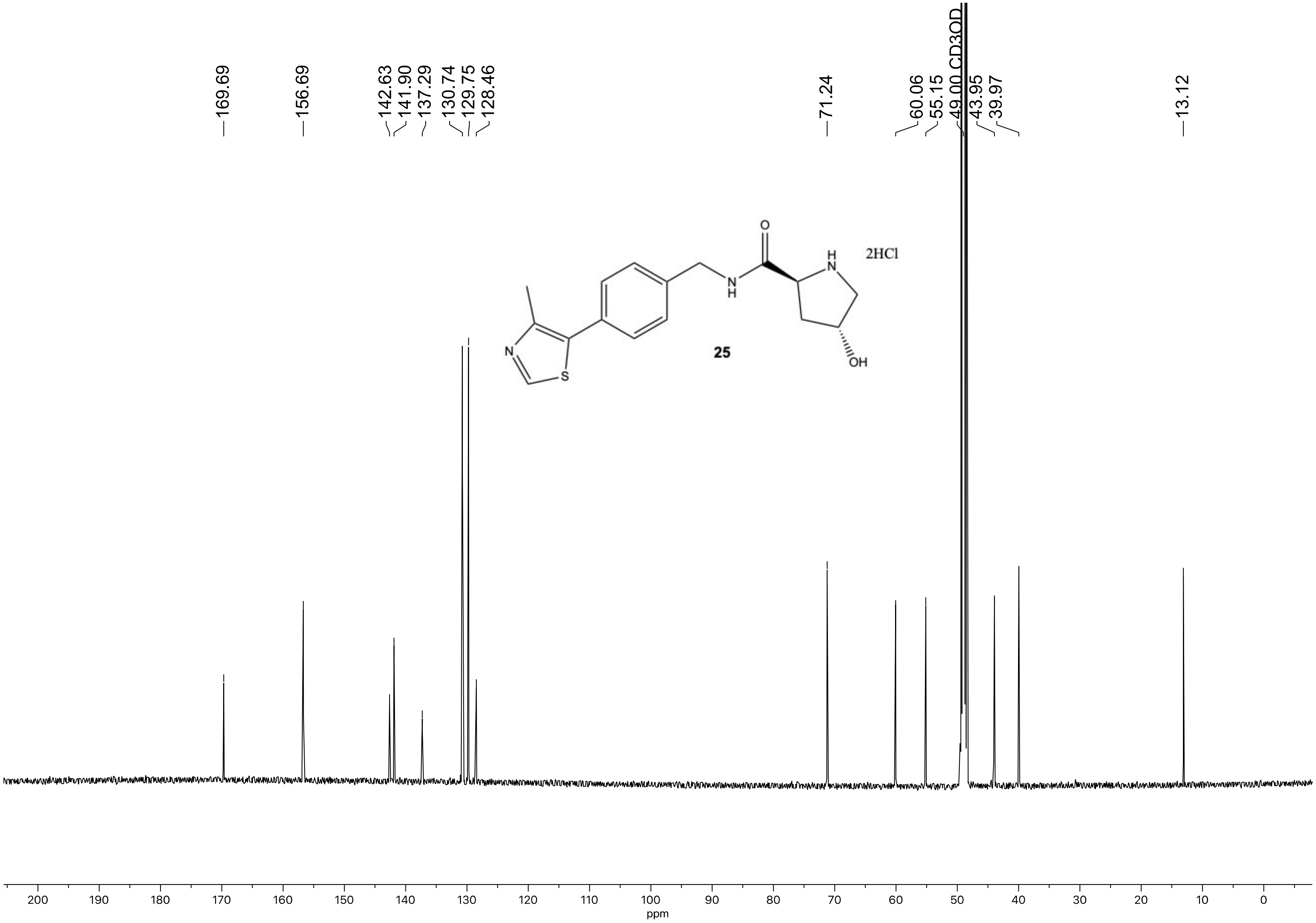

^{13}C NMR (126 MHz, CD_3OD) spectrum of **25** (Hydrochloride salt)

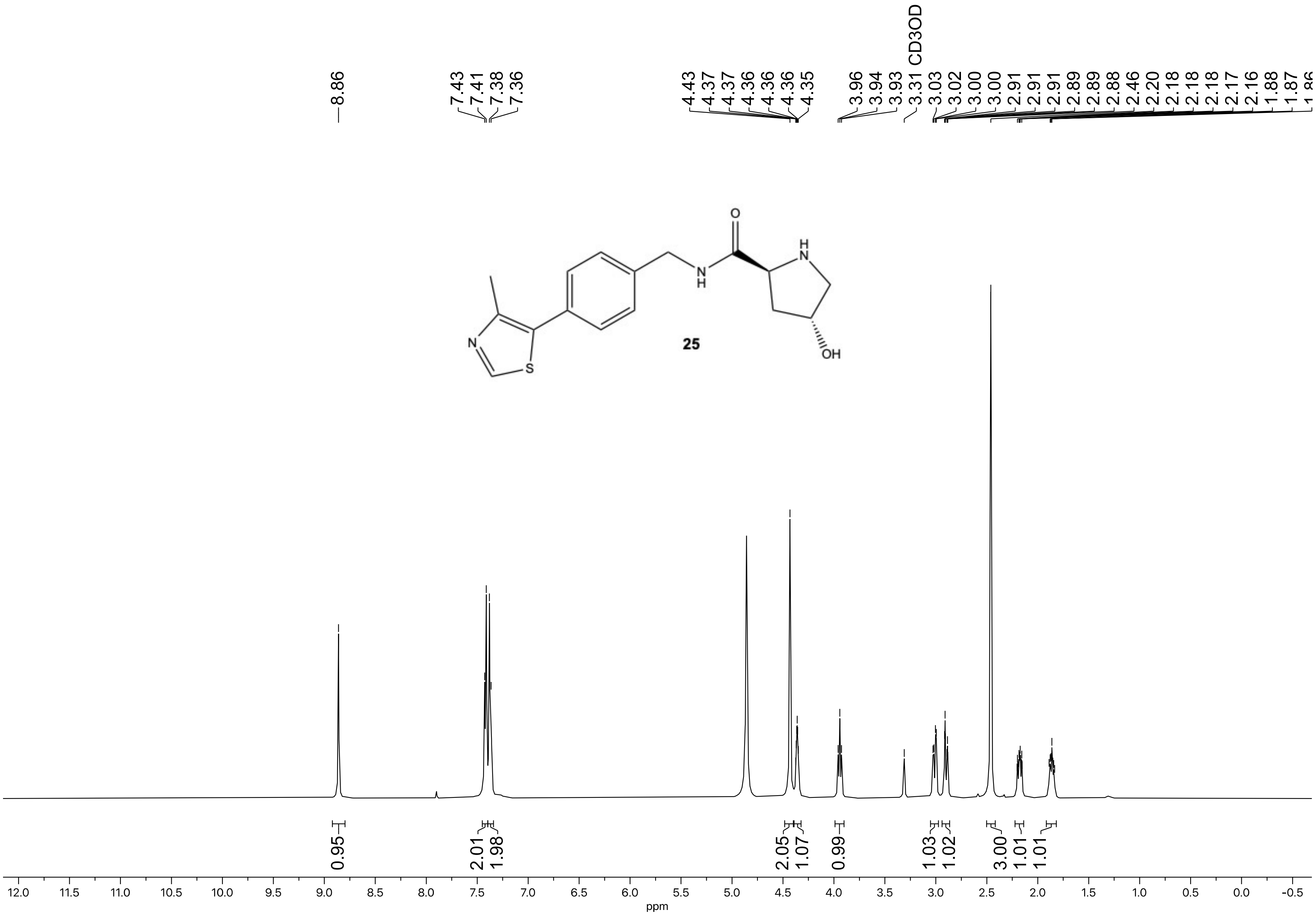

^{1}H NMR (500 MHz, $CDCl_3$) spectrum of **25**

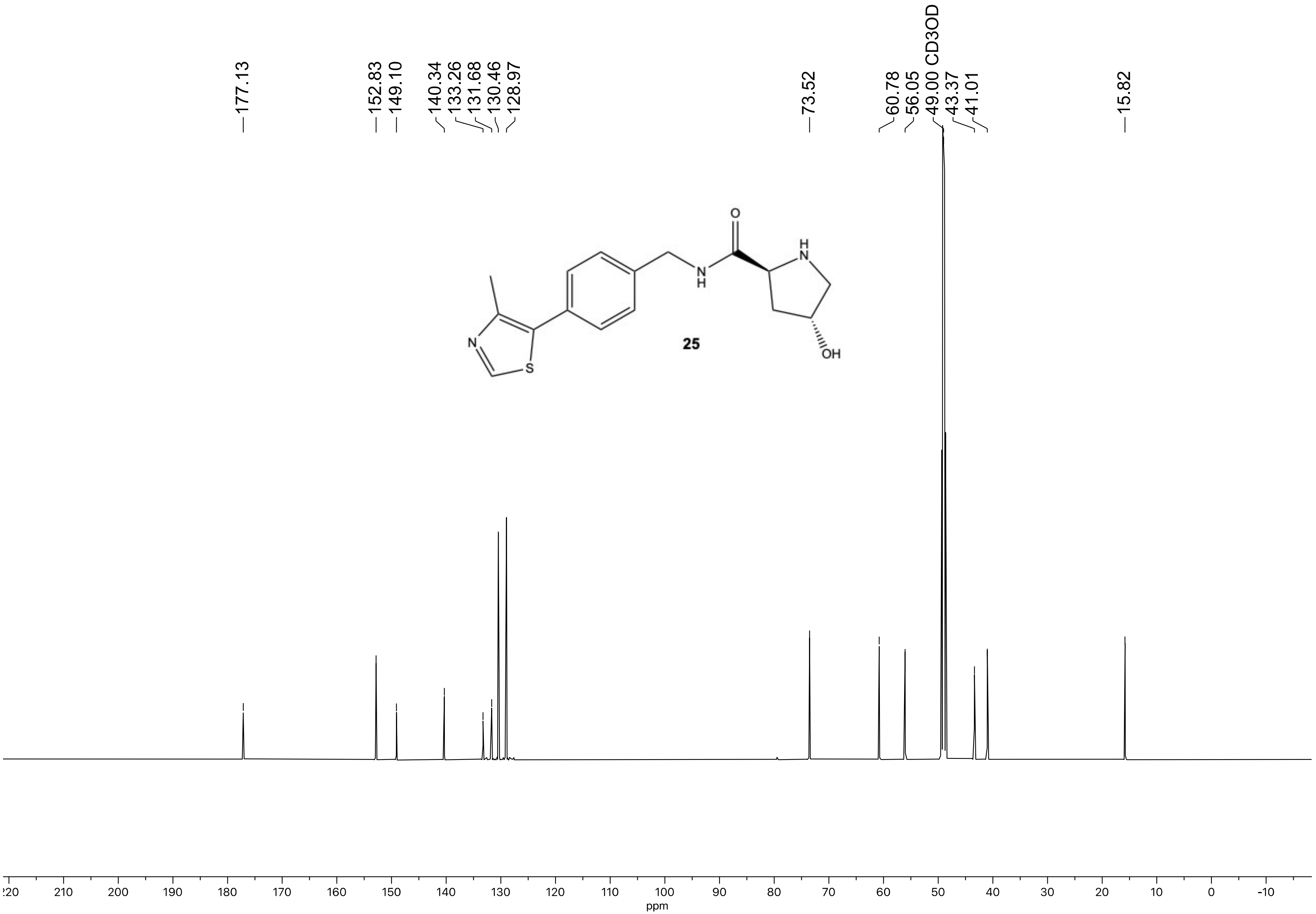

^{13}C NMR (126 MHz, $CDCl_3$) spectrum of **25**

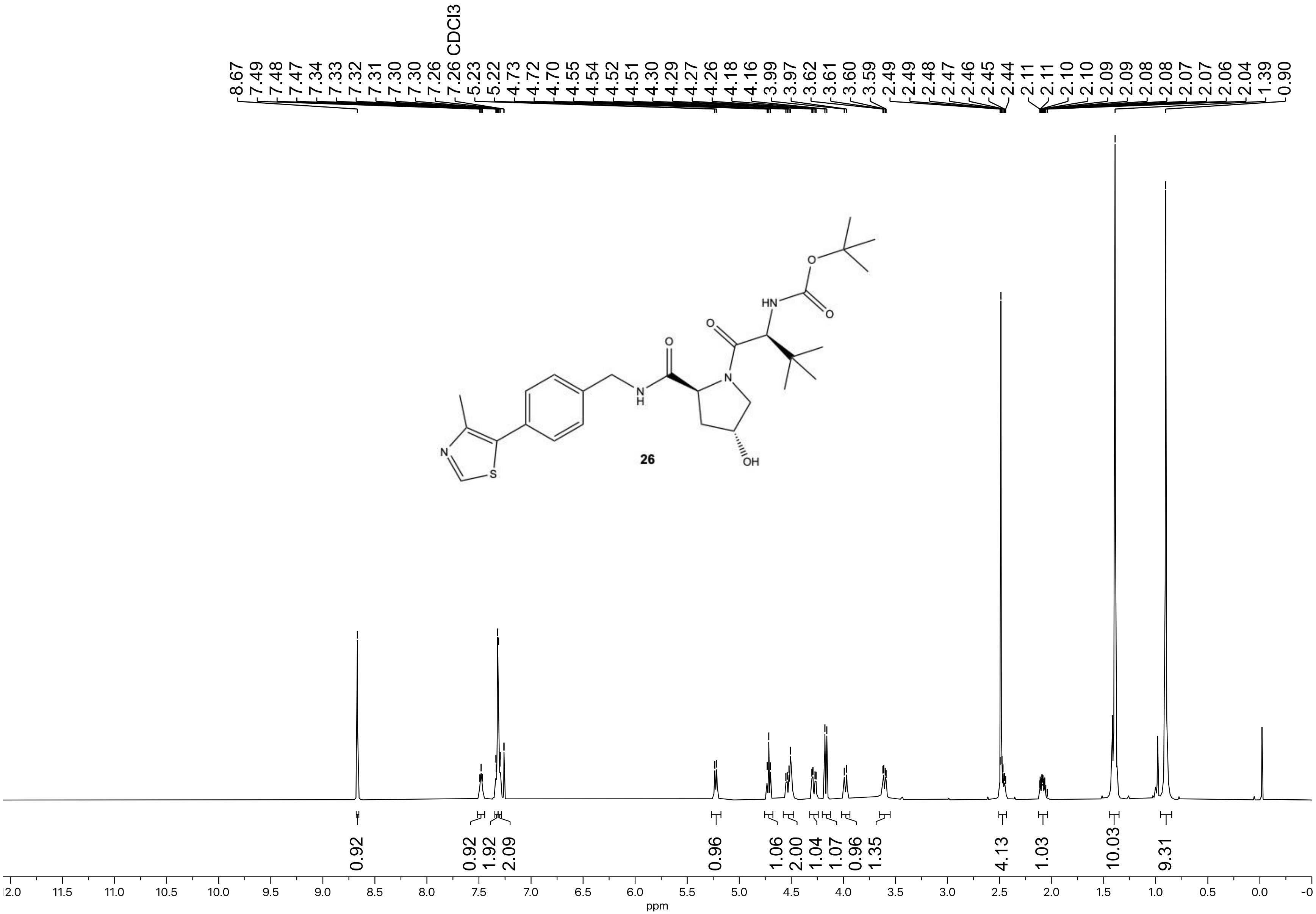

^{1}H NMR (500 MHz, $CDCl_3$) spectrum of **26**

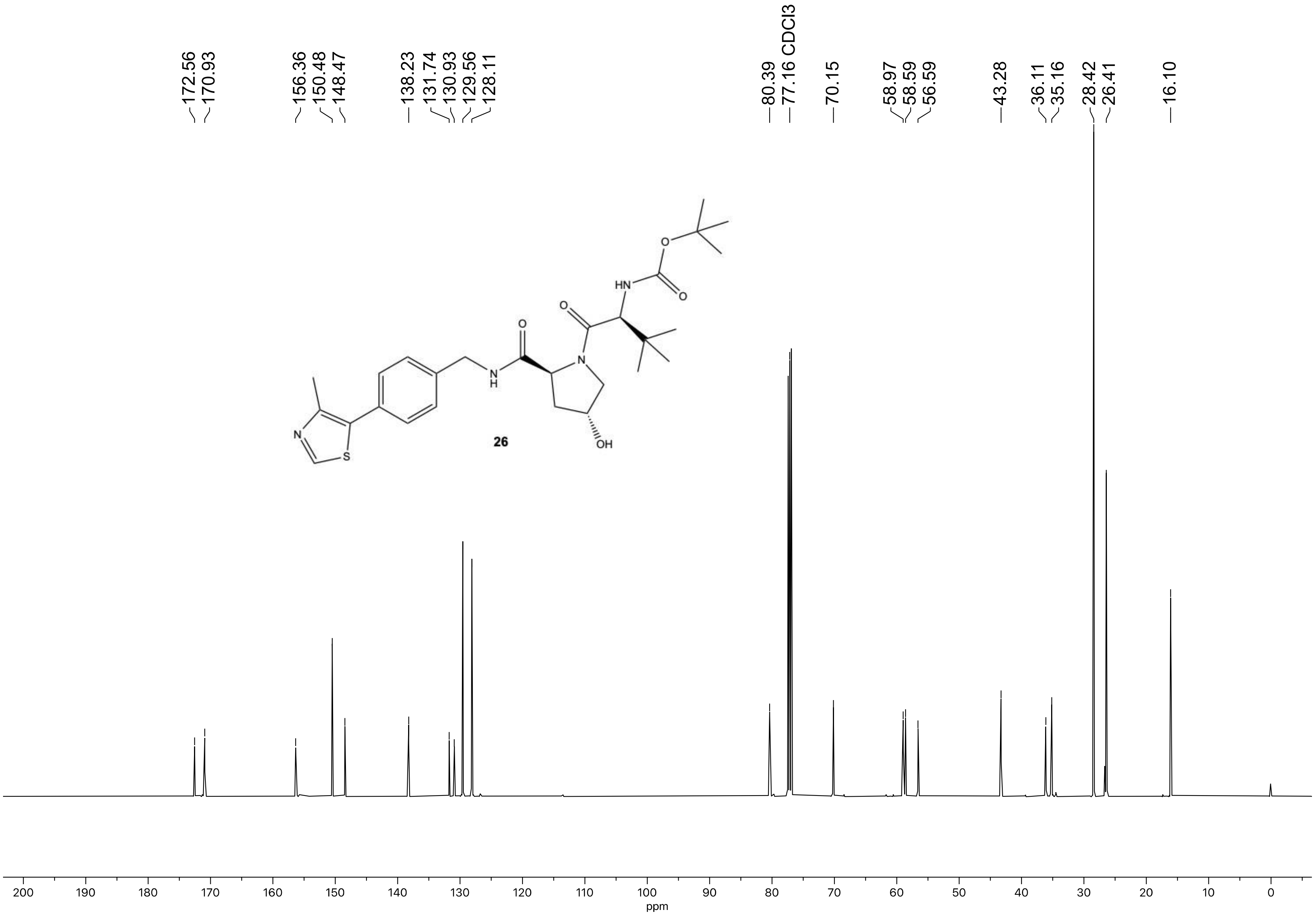

^{13}C NMR (126 MHz, $CDCl_3$) spectrum of **26**

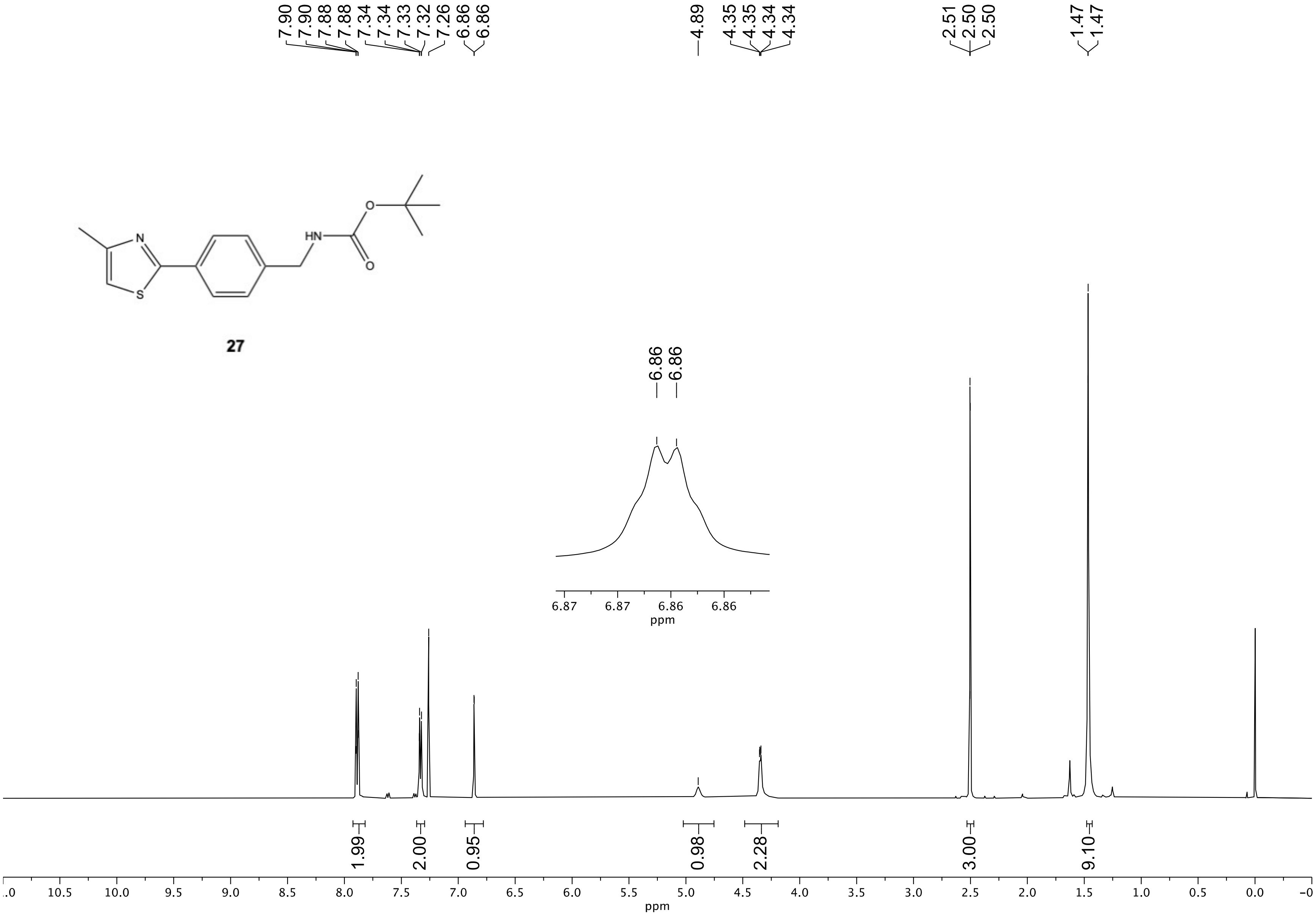

^{1}H NMR (500 MHz, $CDCl_3$) spectrum of **27**

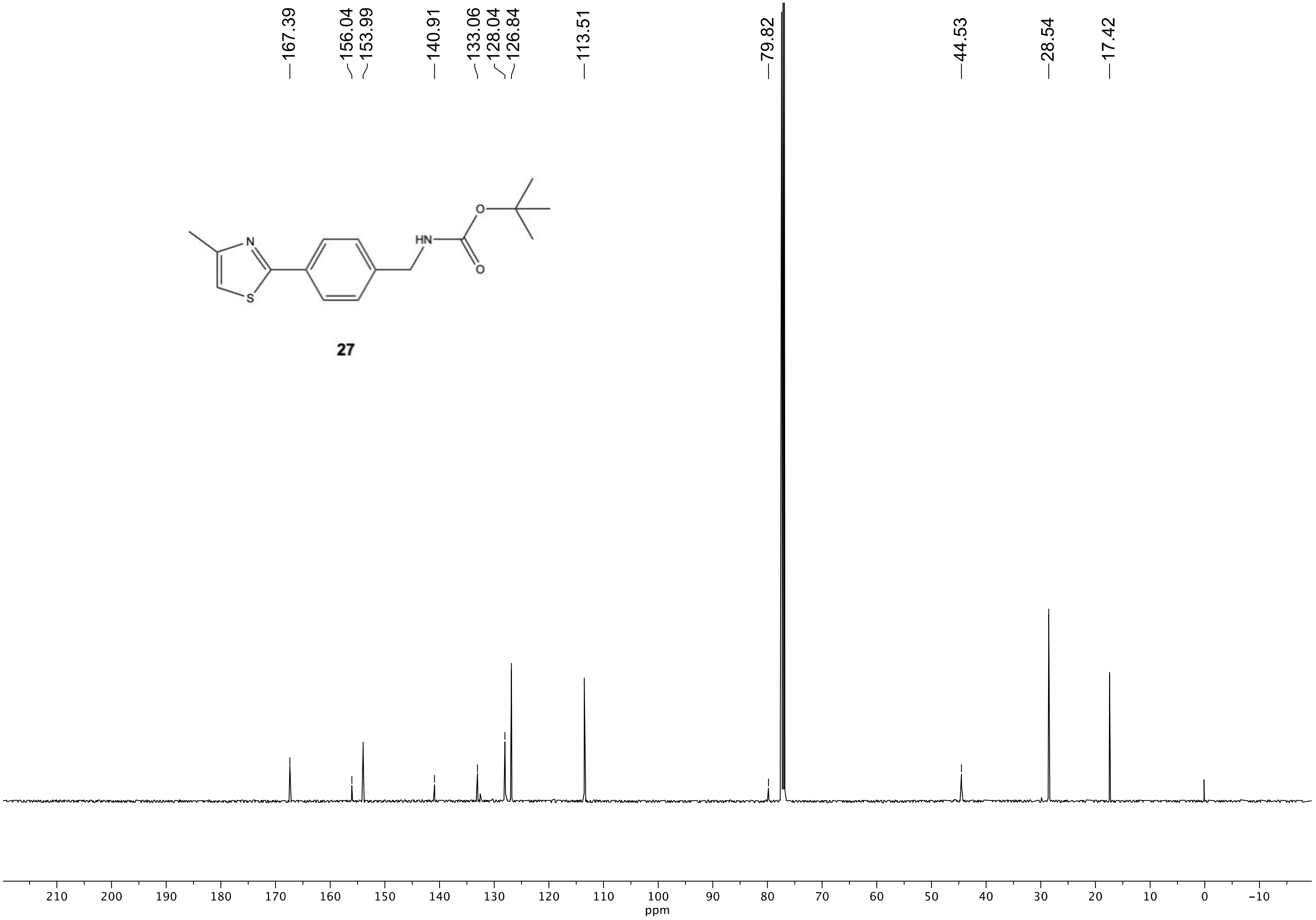

^{13}C NMR (126 MHz, $CDCl_3$) spectrum of **27**

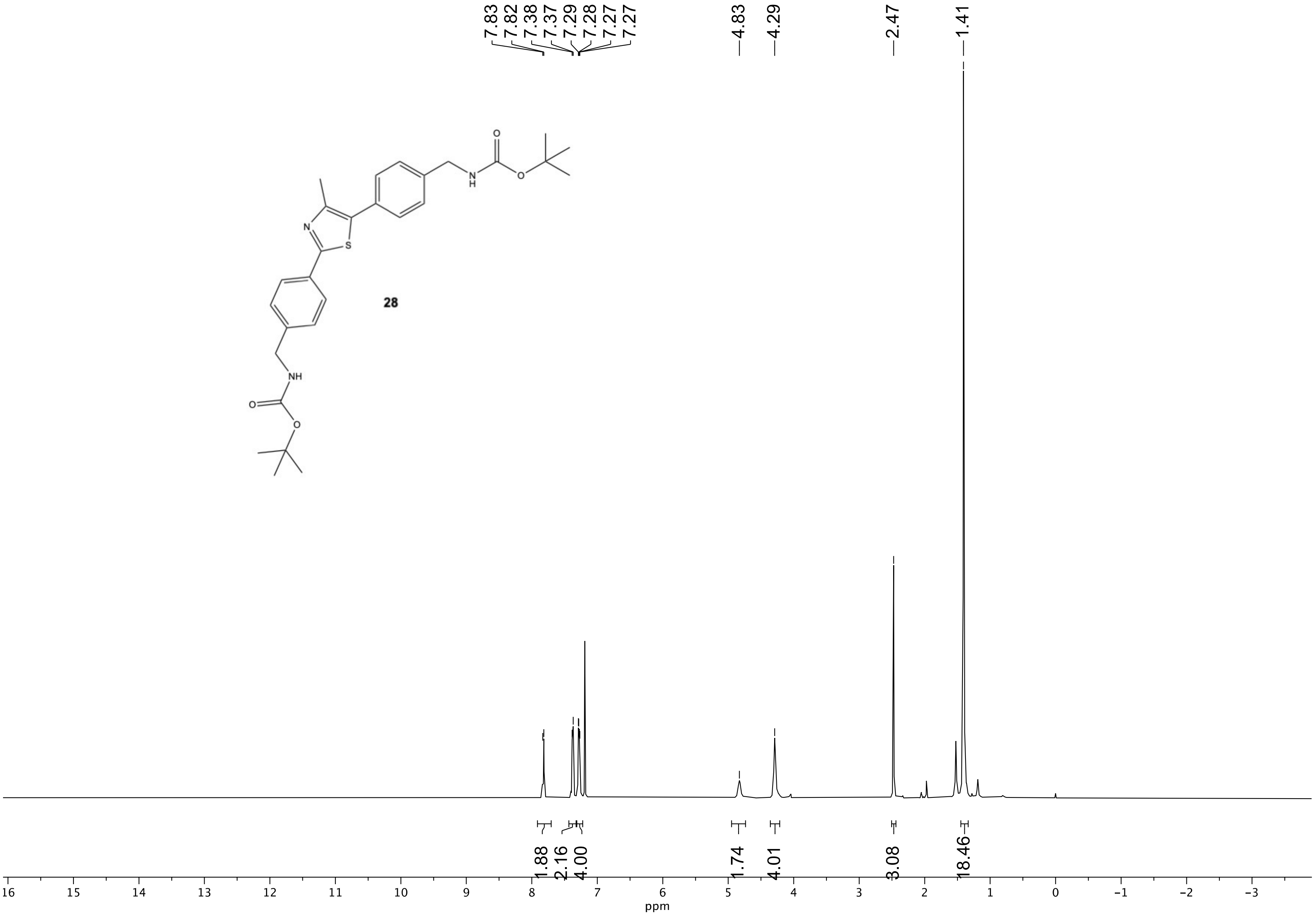

^{1}H NMR (500 MHz, $CDCl_3$) spectrum of **28**

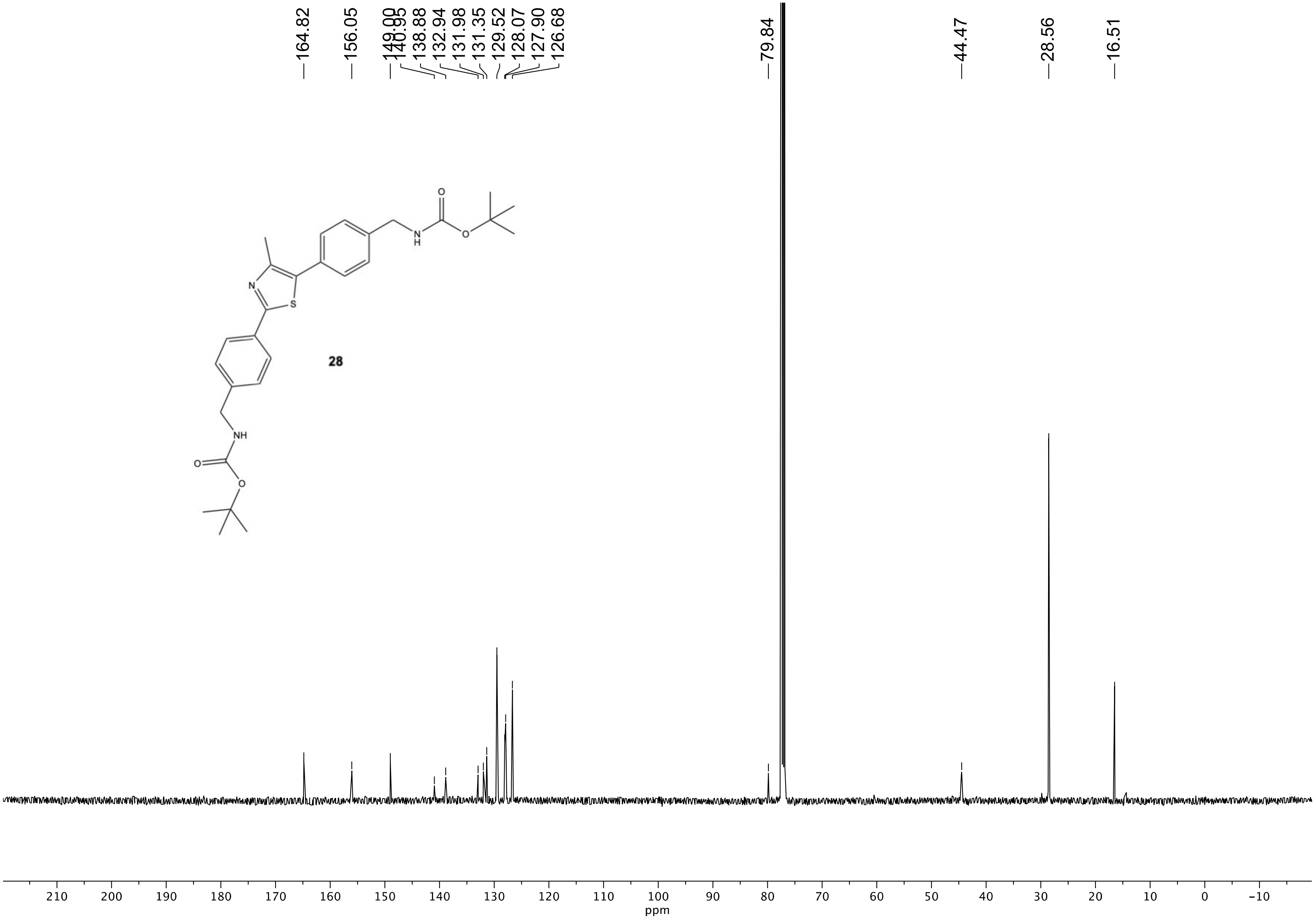

^{13}C NMR (126 MHz, $CDCl_3$) spectrum of **28**

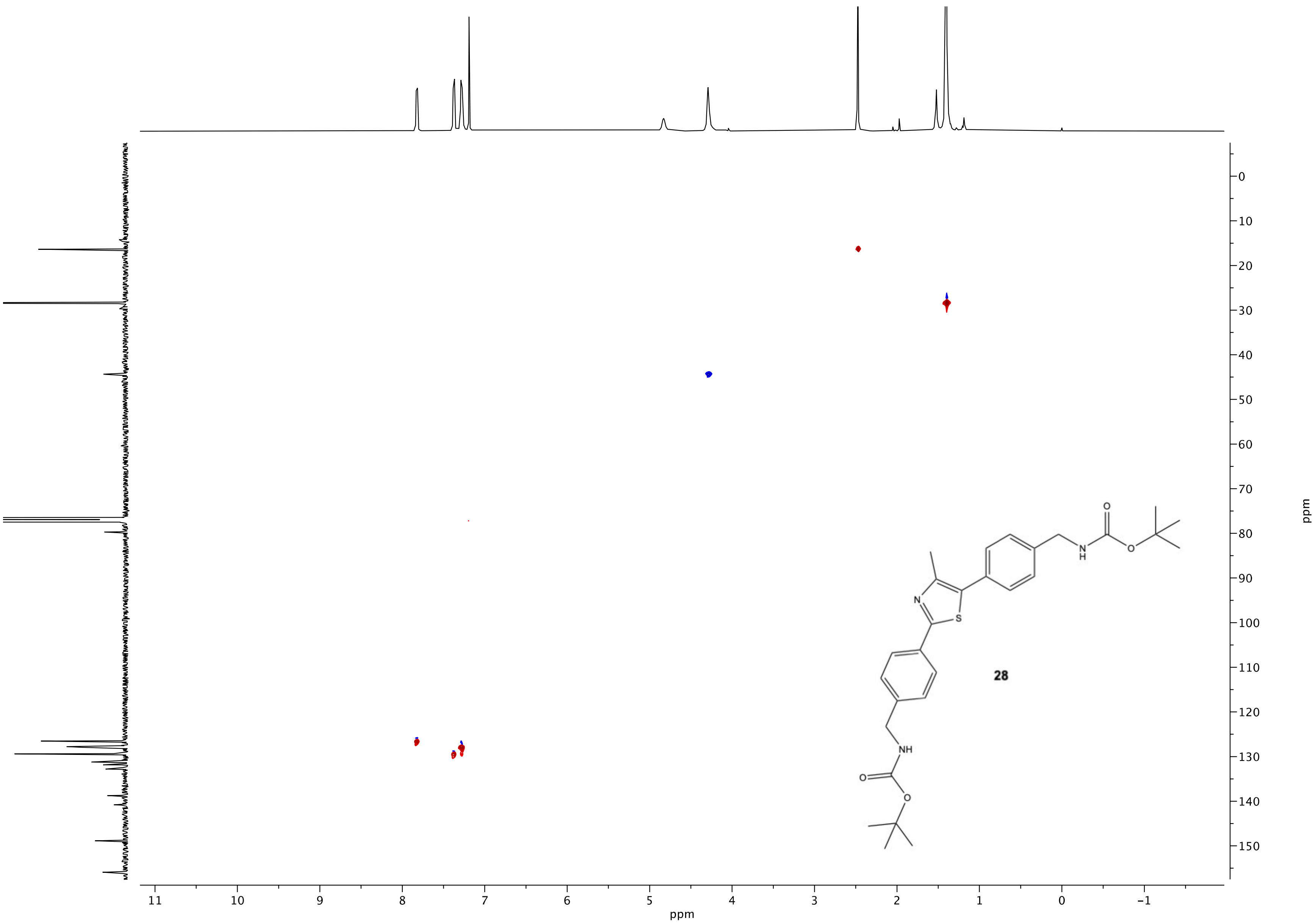

^{1}H-^{13}C HSQC NMR (500 MHz, $CDCl_3$) spectrum of **28**

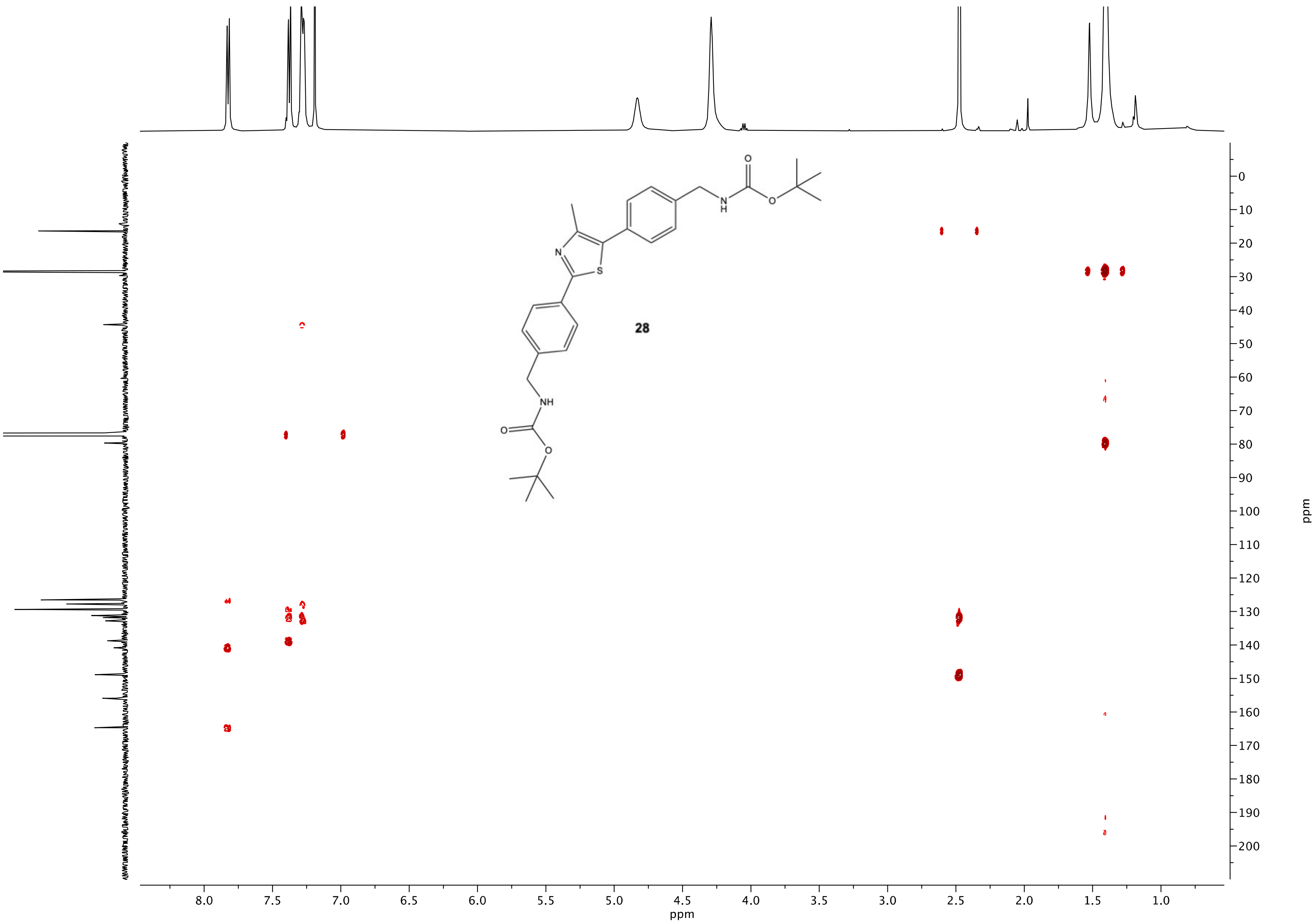

^1H-^{13}C HMBC NMR (500 MHz, $CDCl_3$) spectrum of **28**

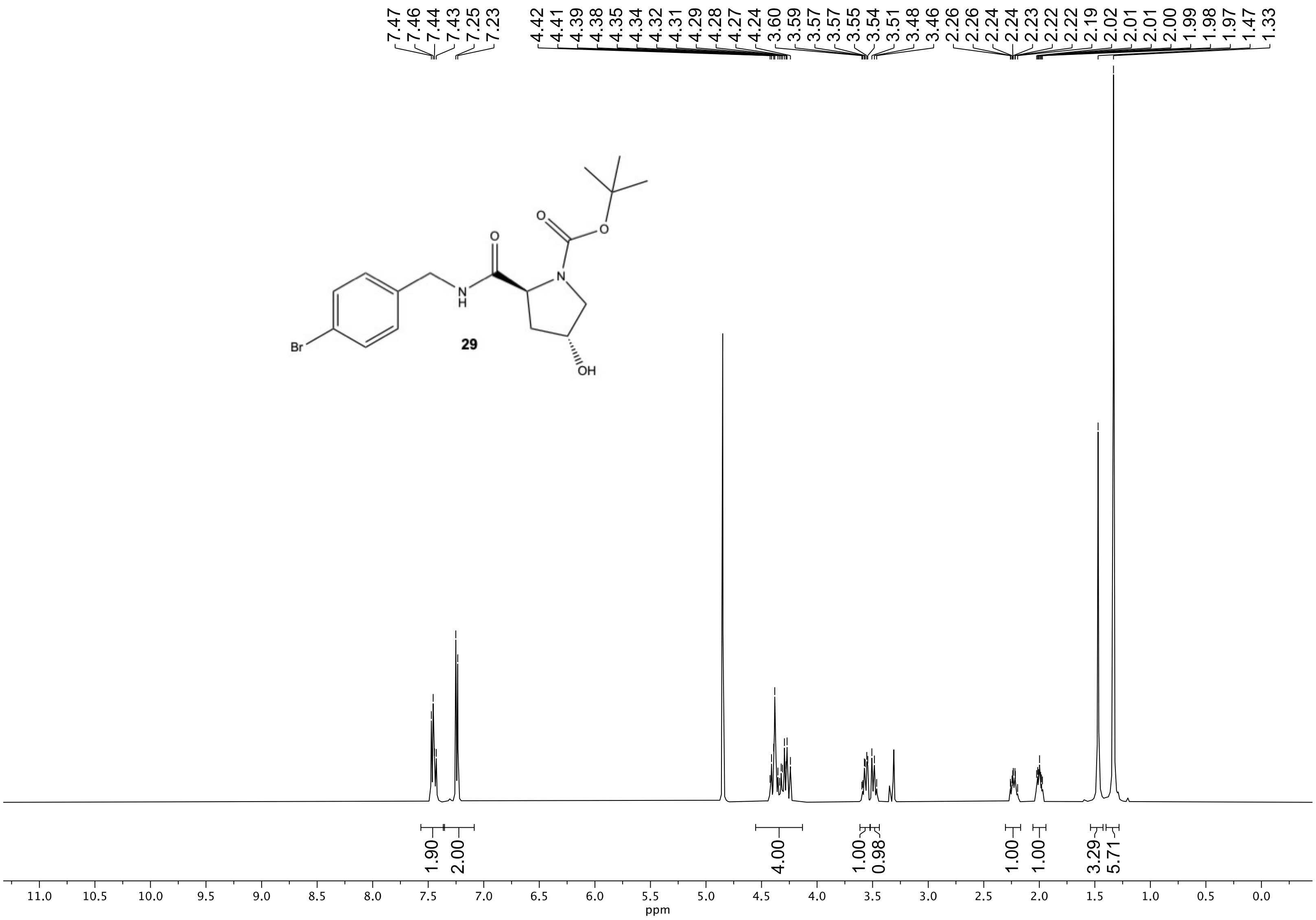

^{1}H NMR (500 MHz, $(CD_3)_2CO$) spectrum of **29**

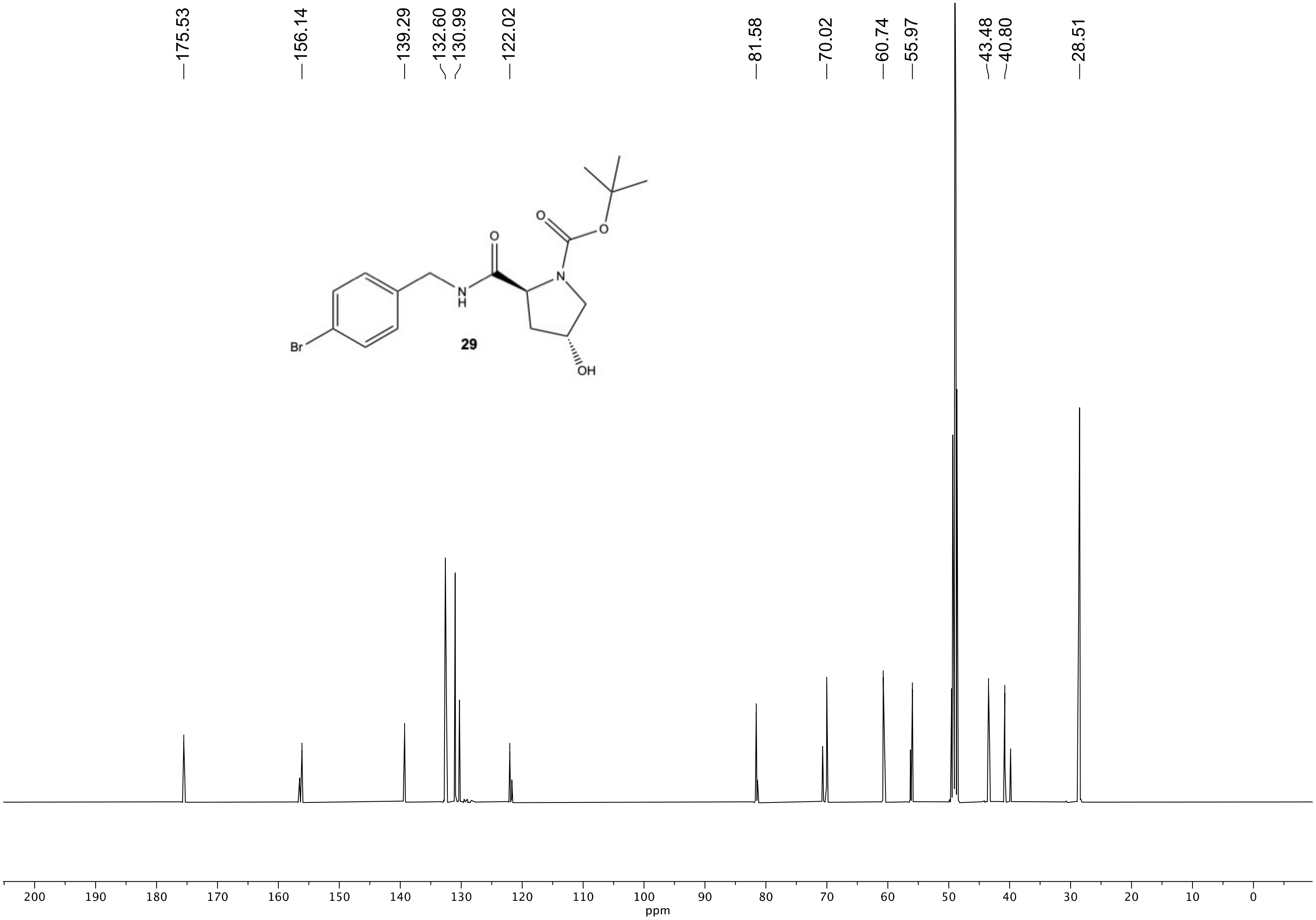

^{13}C NMR (126 MHz, $(CD_3)_2CO$) spectrum of **29**

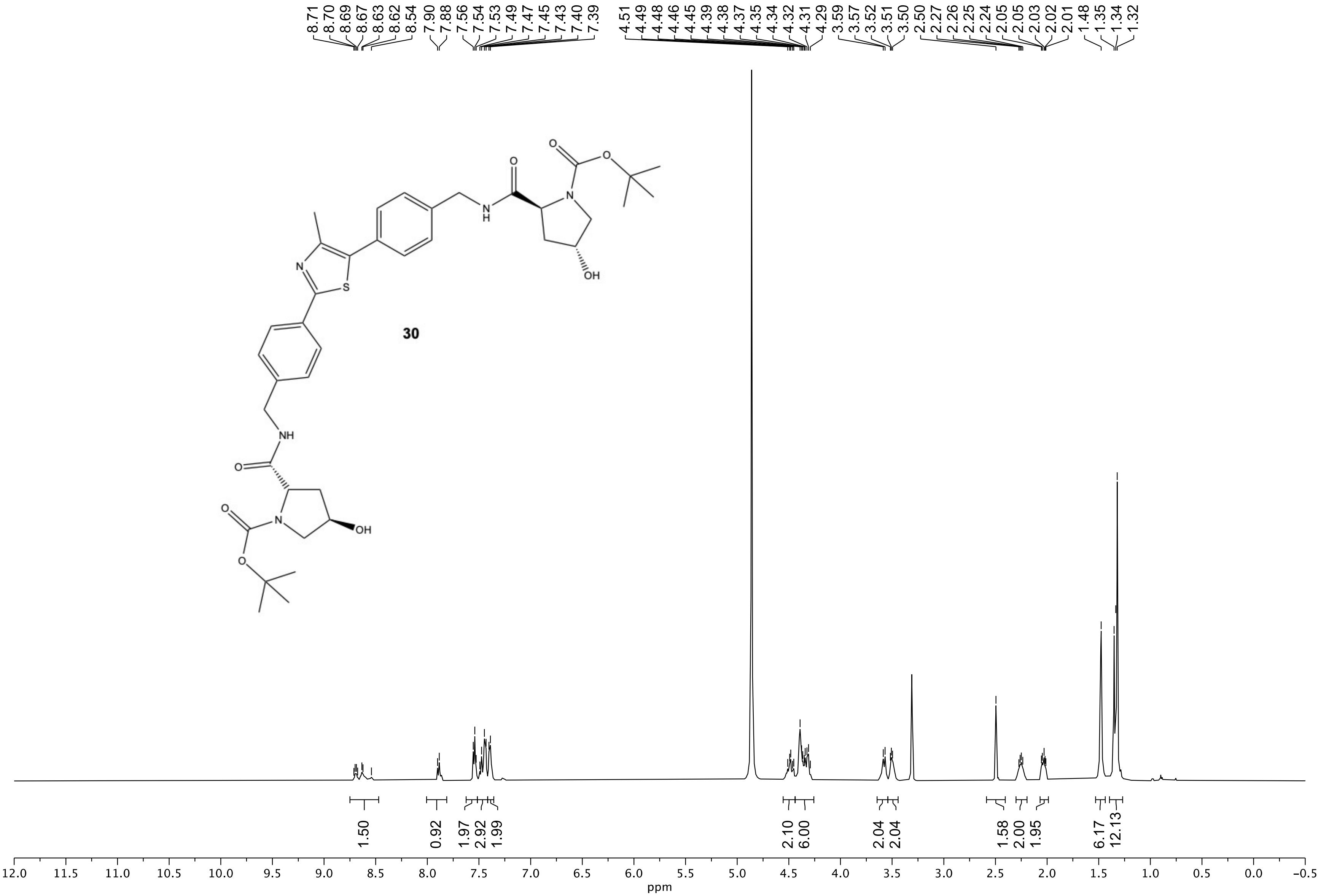

^{1}H NMR (500 MHz, CD_3OD) spectrum of **30**

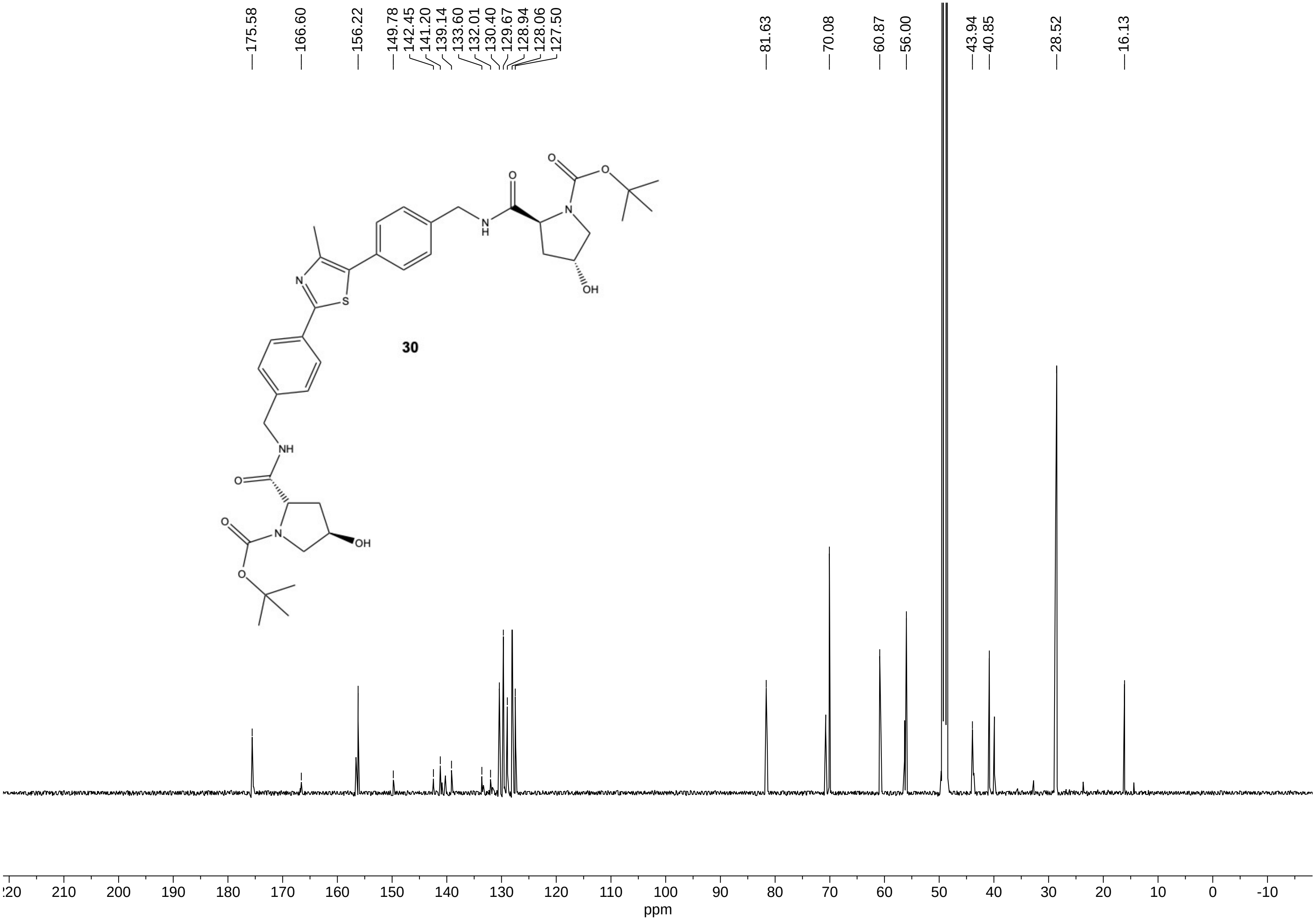

^{13}C NMR (126 MHz, CD_3OD) spectrum of **30**

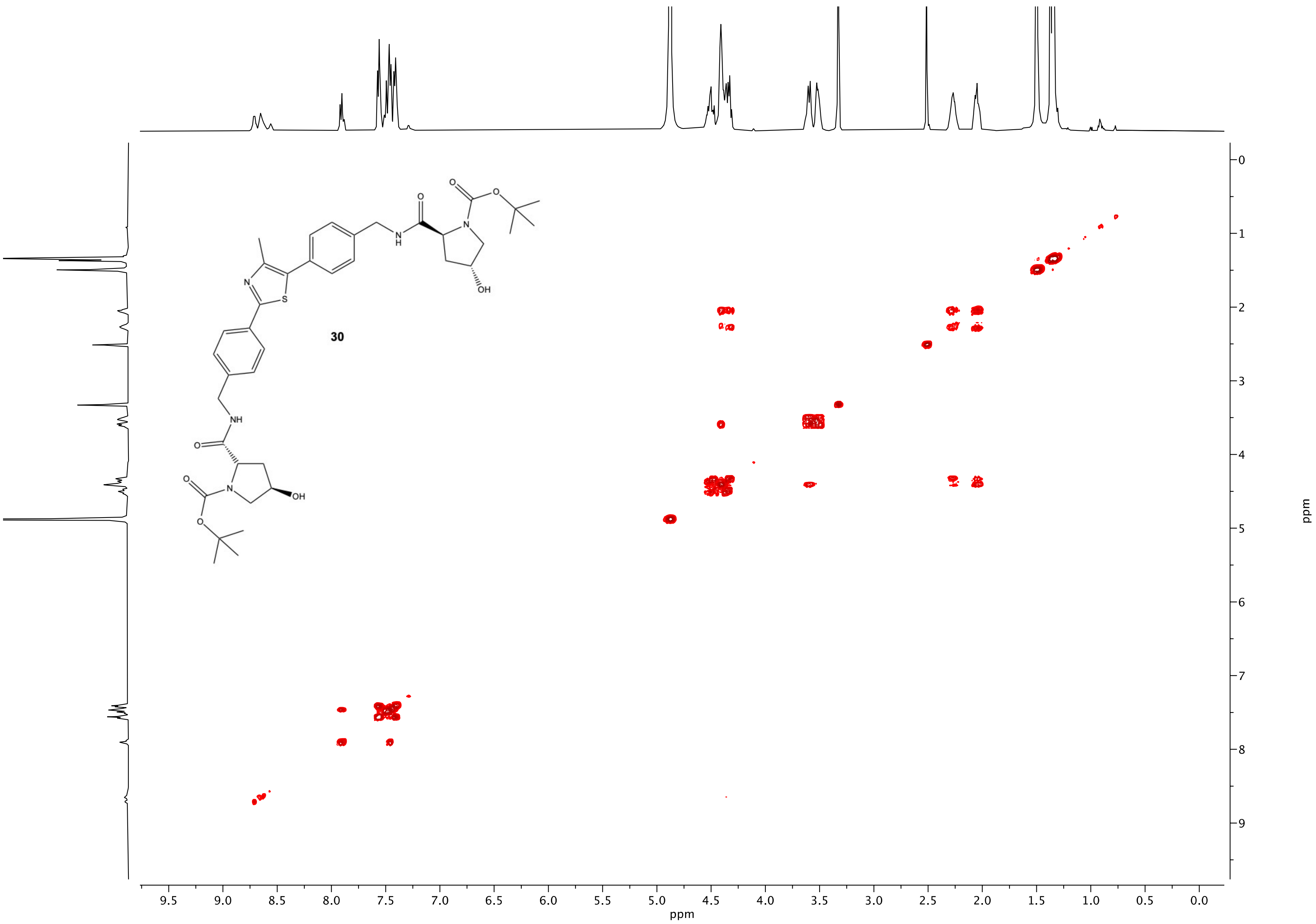

^{1}H-^{1}H COSY NMR (500 MHz, CD_3OD) spectrum of **30**

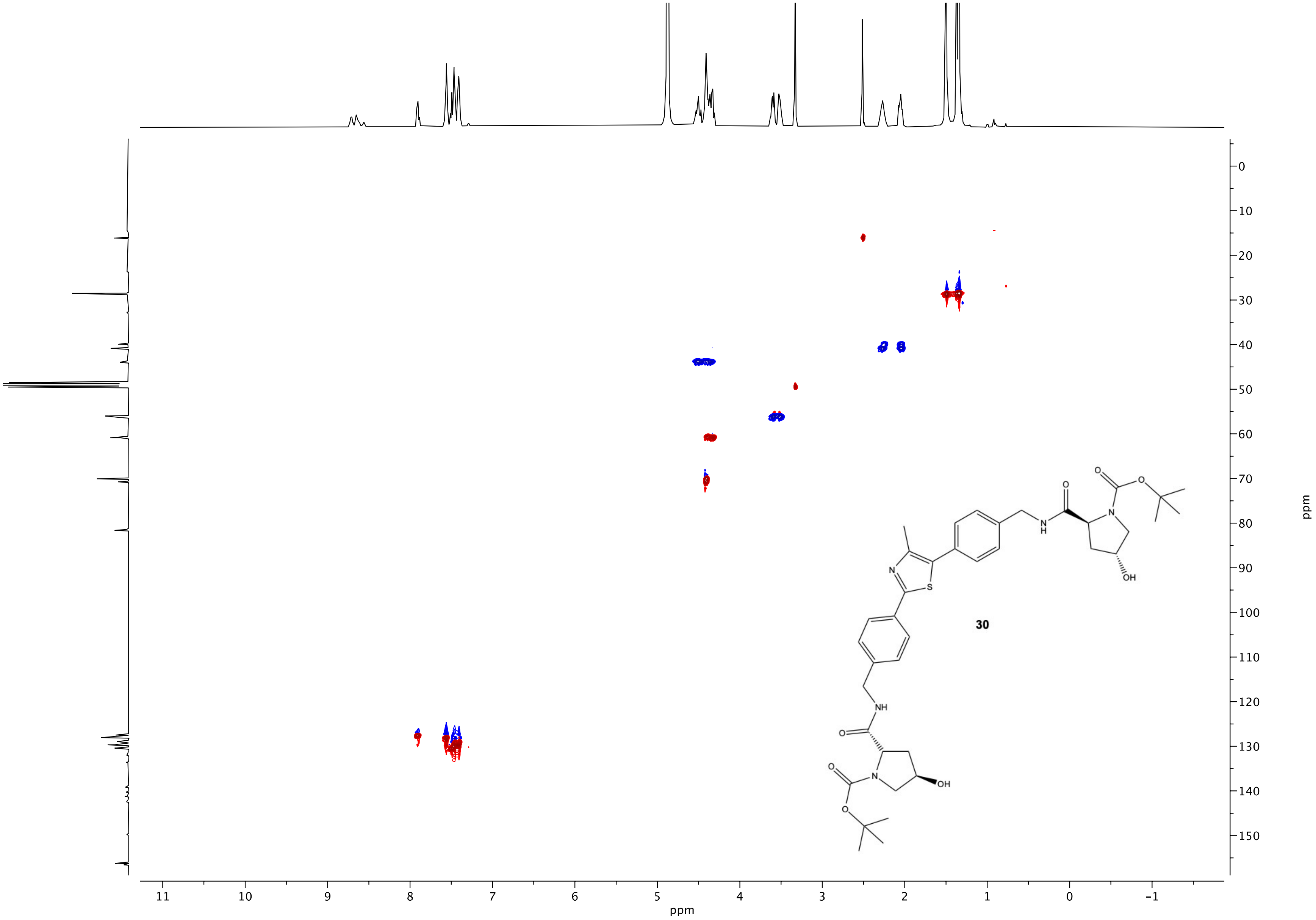

^{1}H-^{13}C HSQC NMR (500 MHz, CD_3OD) spectrum of **30**

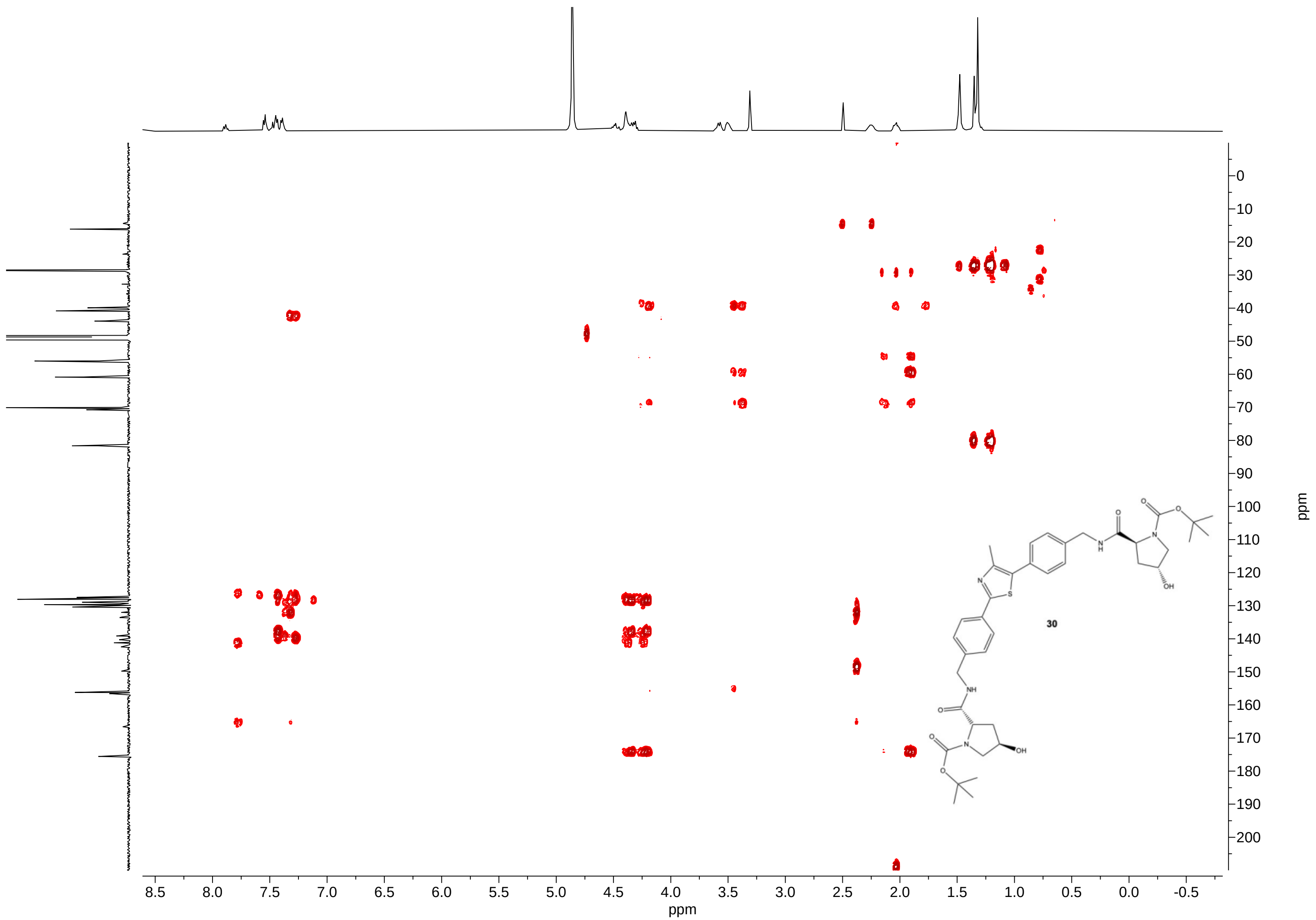

^{1}H-^{13}C HMBC NMR (500 MHz, CD_3OD) spectrum of **30**

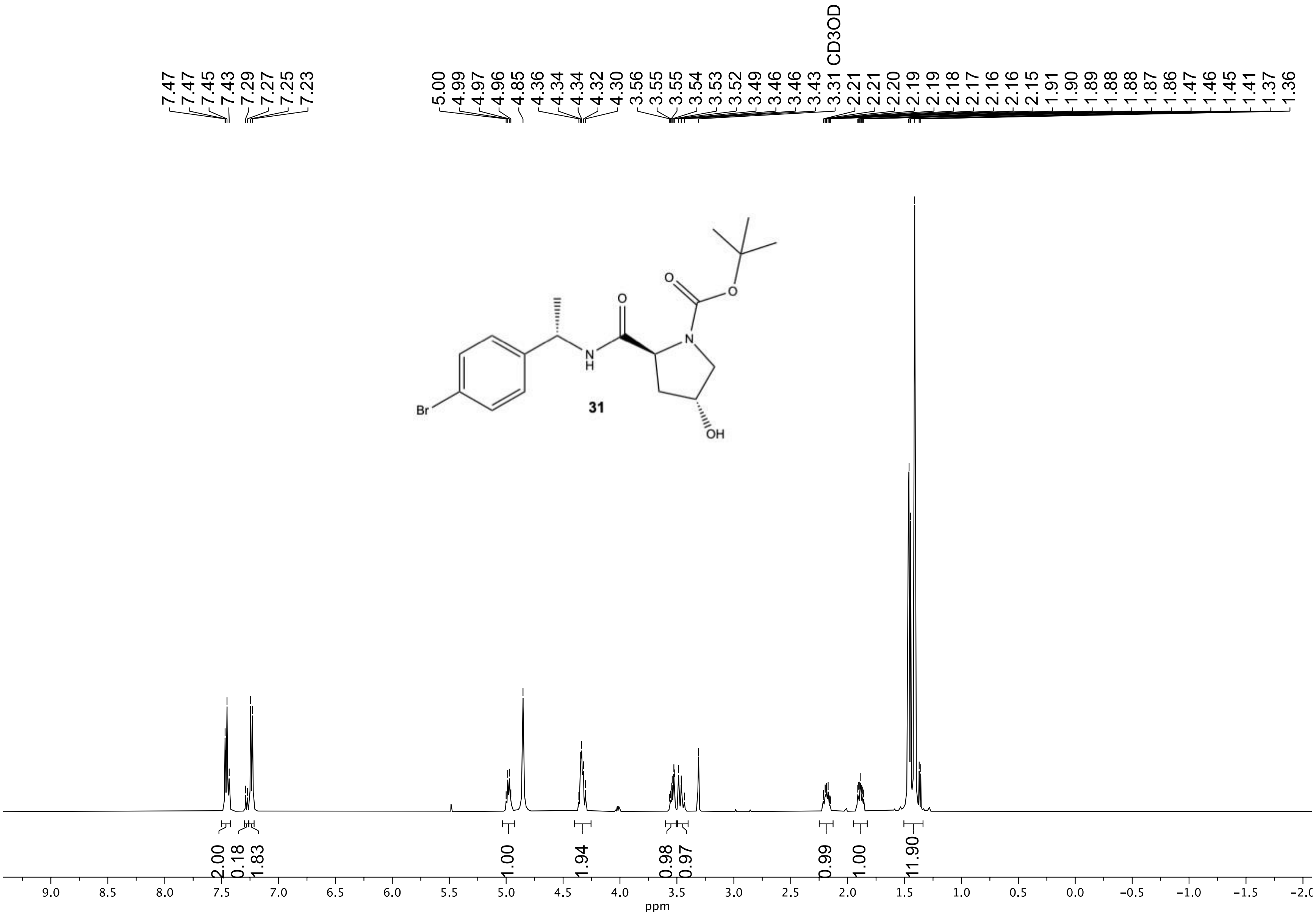

^{1}H NMR (500 MHz, $(CD_3)_2CO$) spectrum of **31**

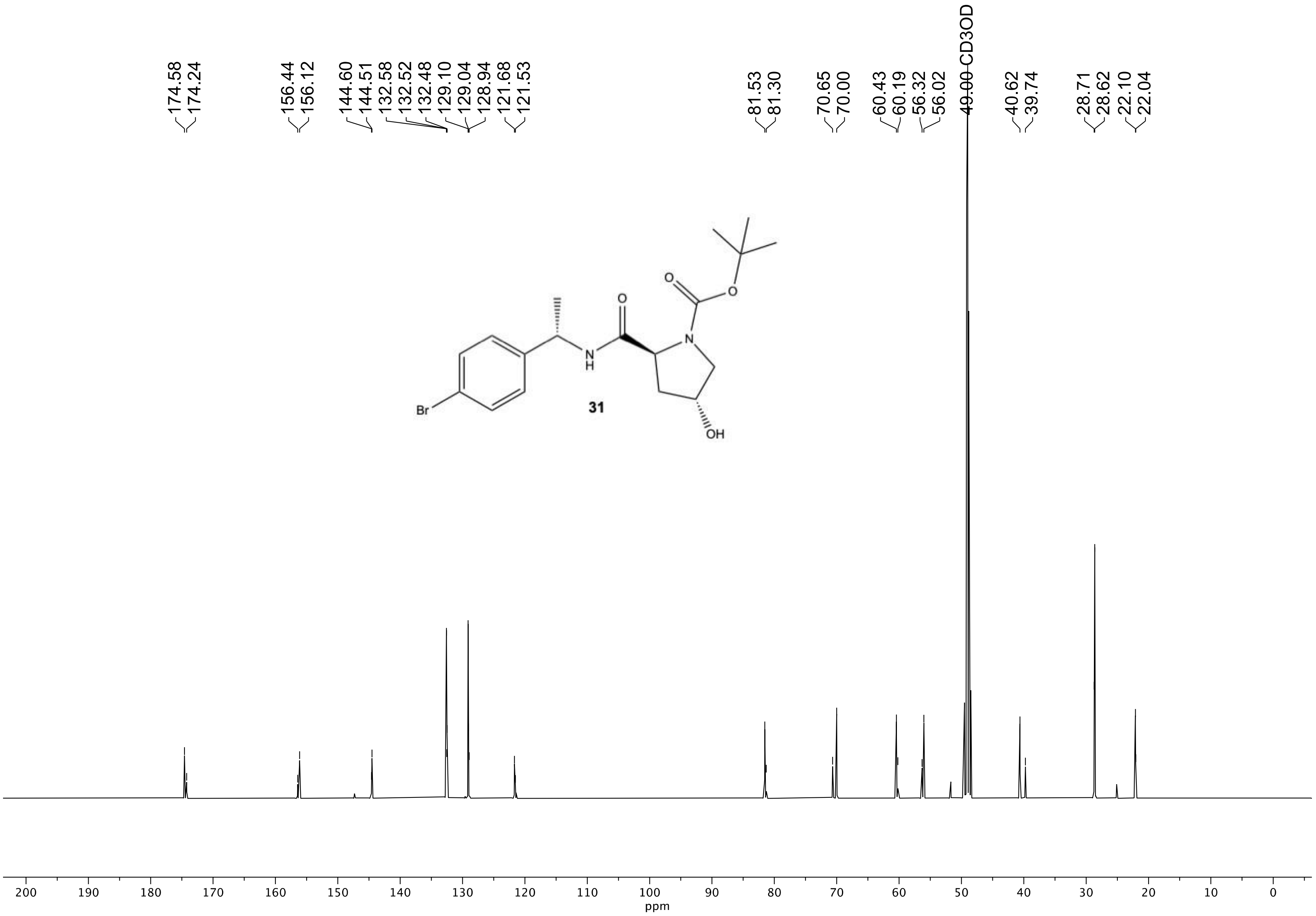

^{13}C NMR (126 MHz, $(CD_3)_2CO$) spectrum of **31**

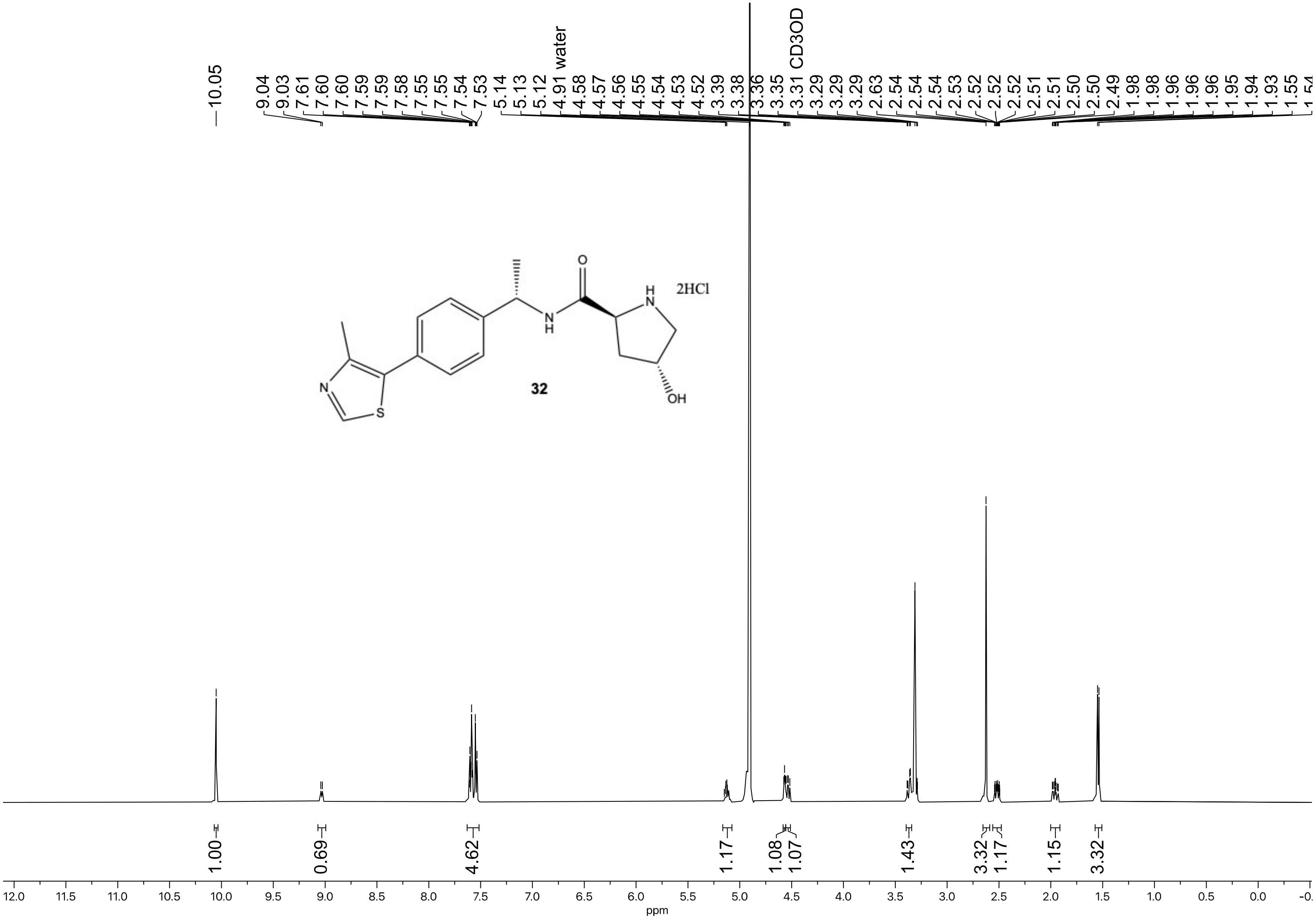

^{1}H NMR (500 MHz, CD_3OD) spectrum of **32** (Hydrochloride salt)

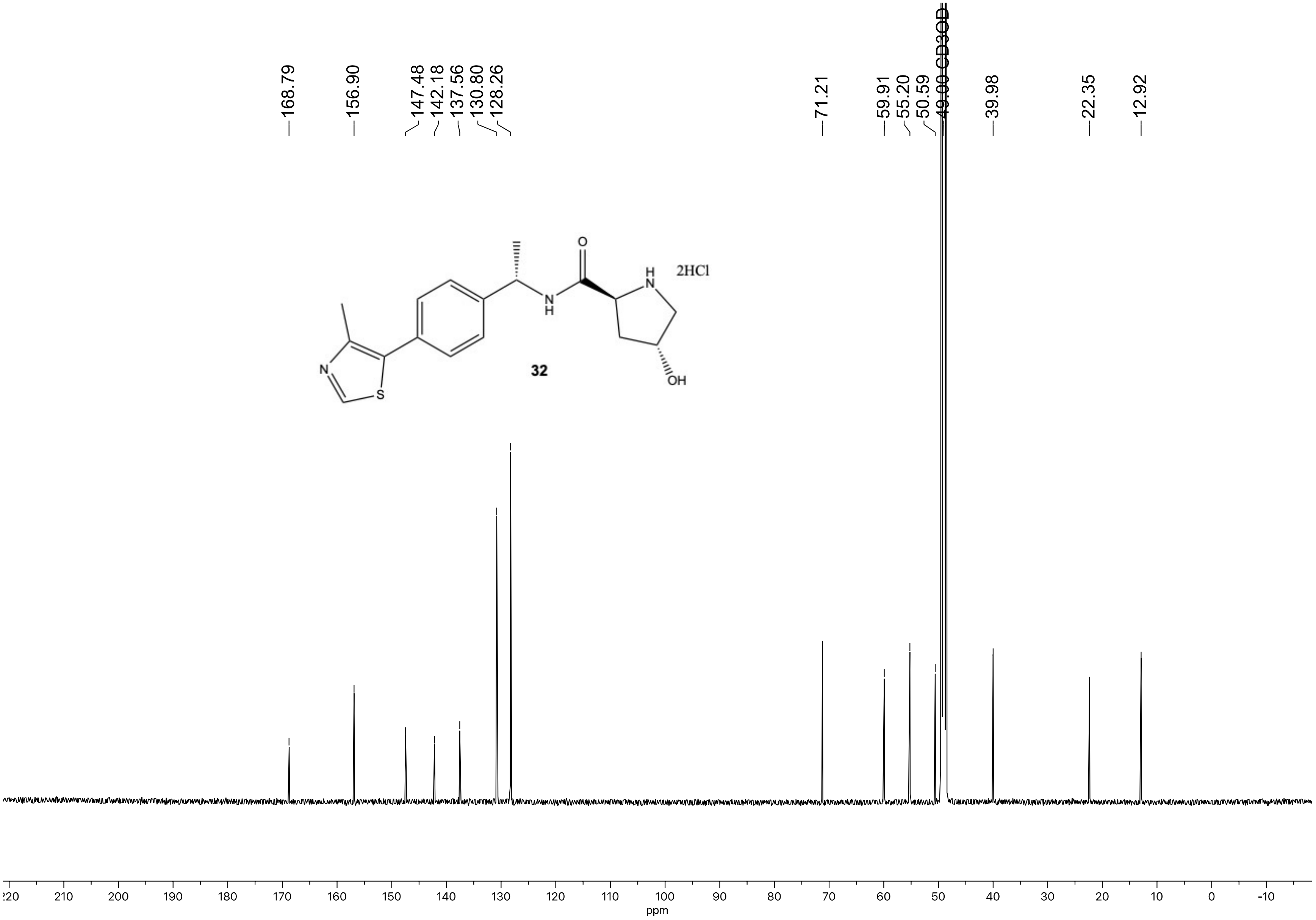

^{13}C NMR (125 MHz, CD_3OD) spectrum of **32** (Hydrochloride salt)

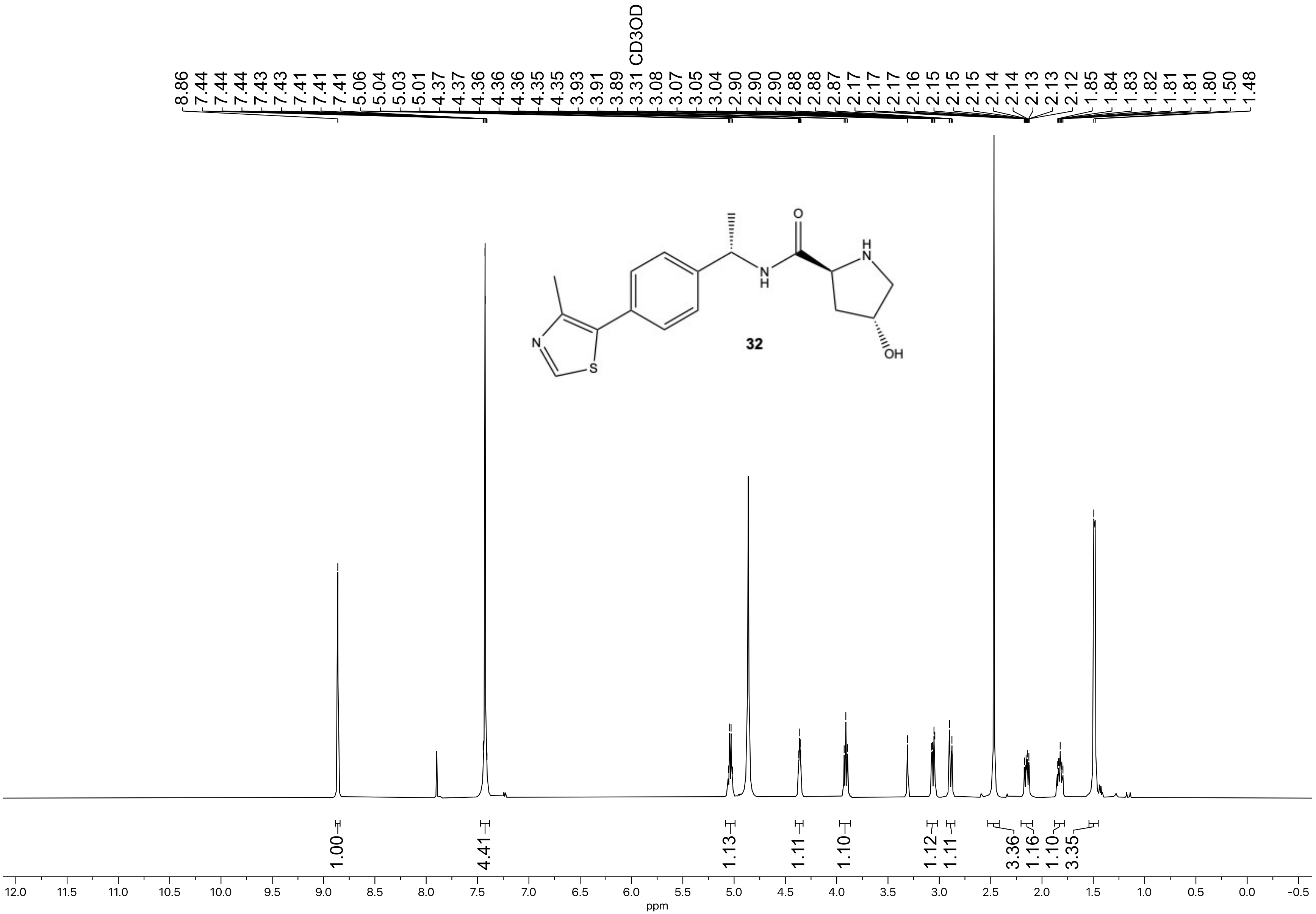

^{1}H NMR (500 MHz, CD_3OD) spectrum of **32**

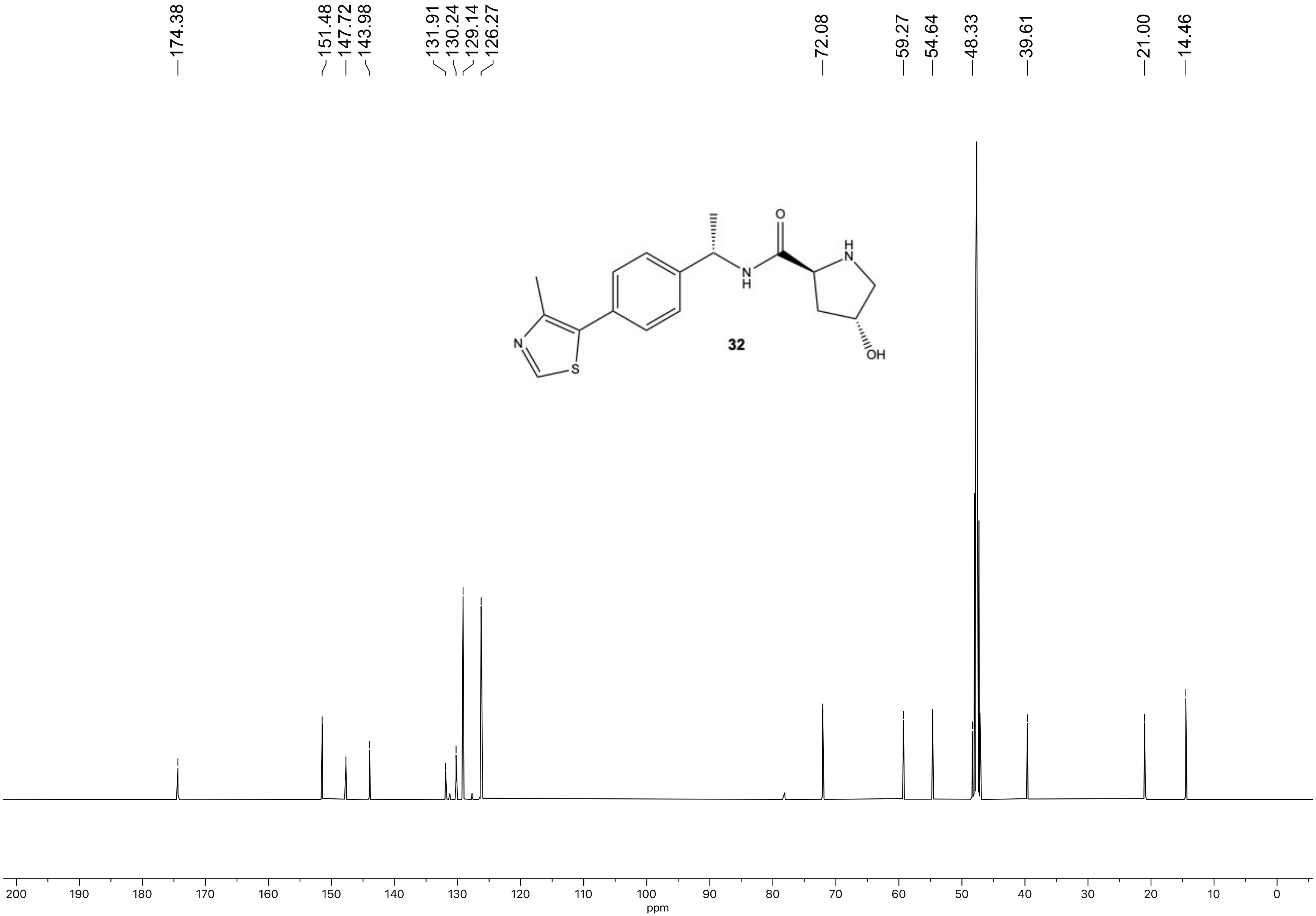

^{13}C NMR (126 MHz, CD_3OD) spectrum of **32**

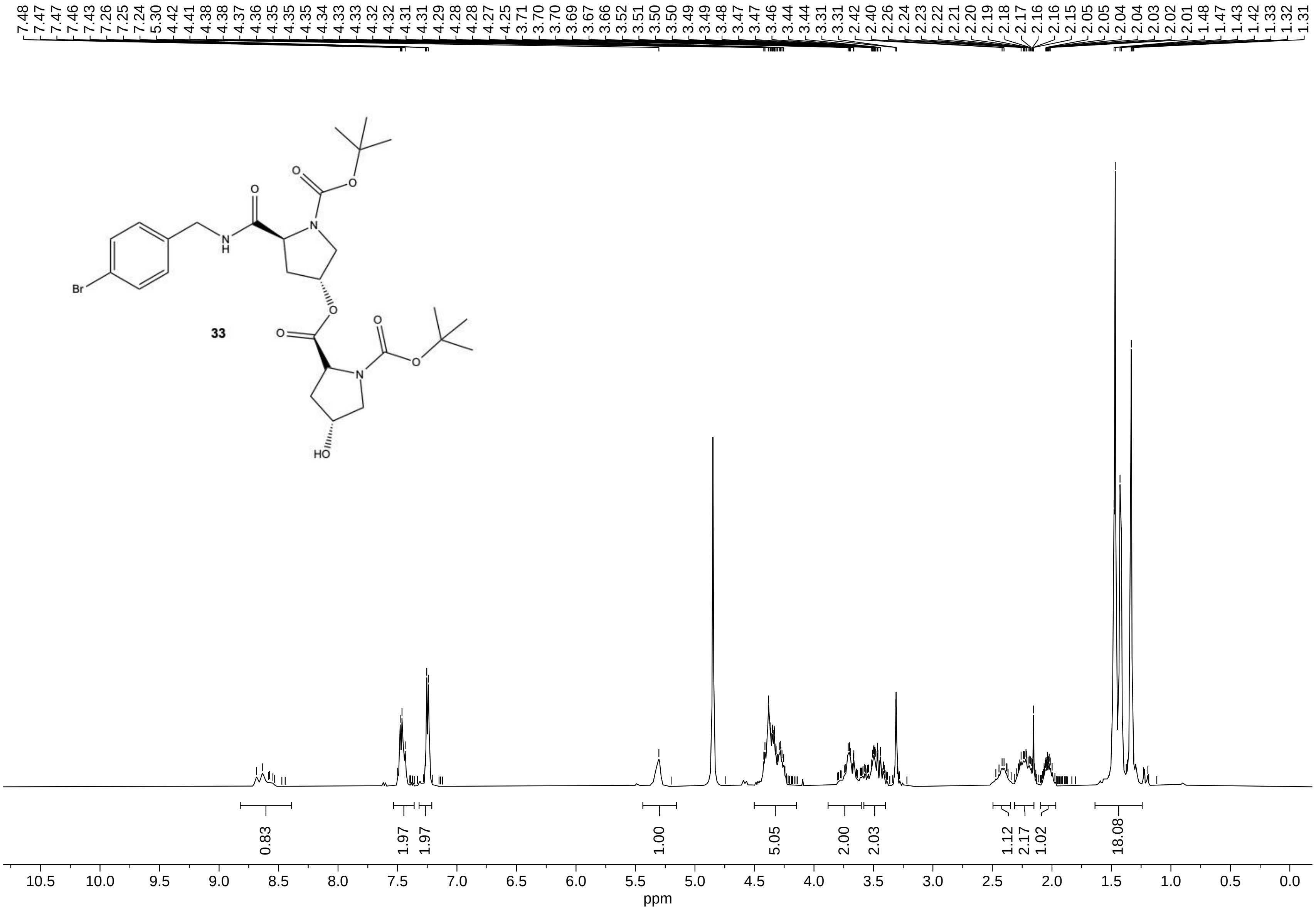

^{1}H NMR (500 MHz, CD_3OD) spectrum of **33**

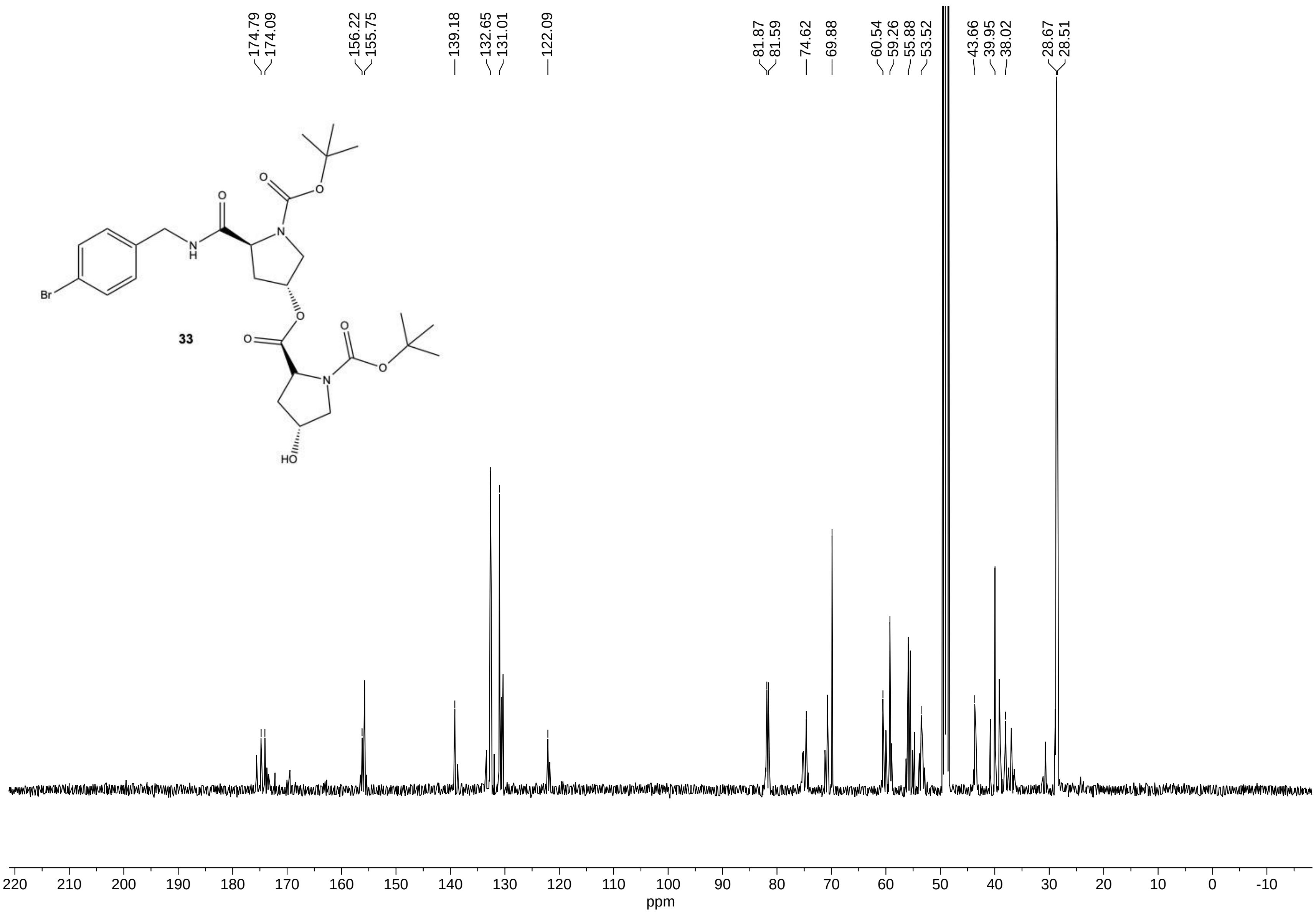

^{13}C NMR (126 MHz, CD_3OD) spectrum of **33**

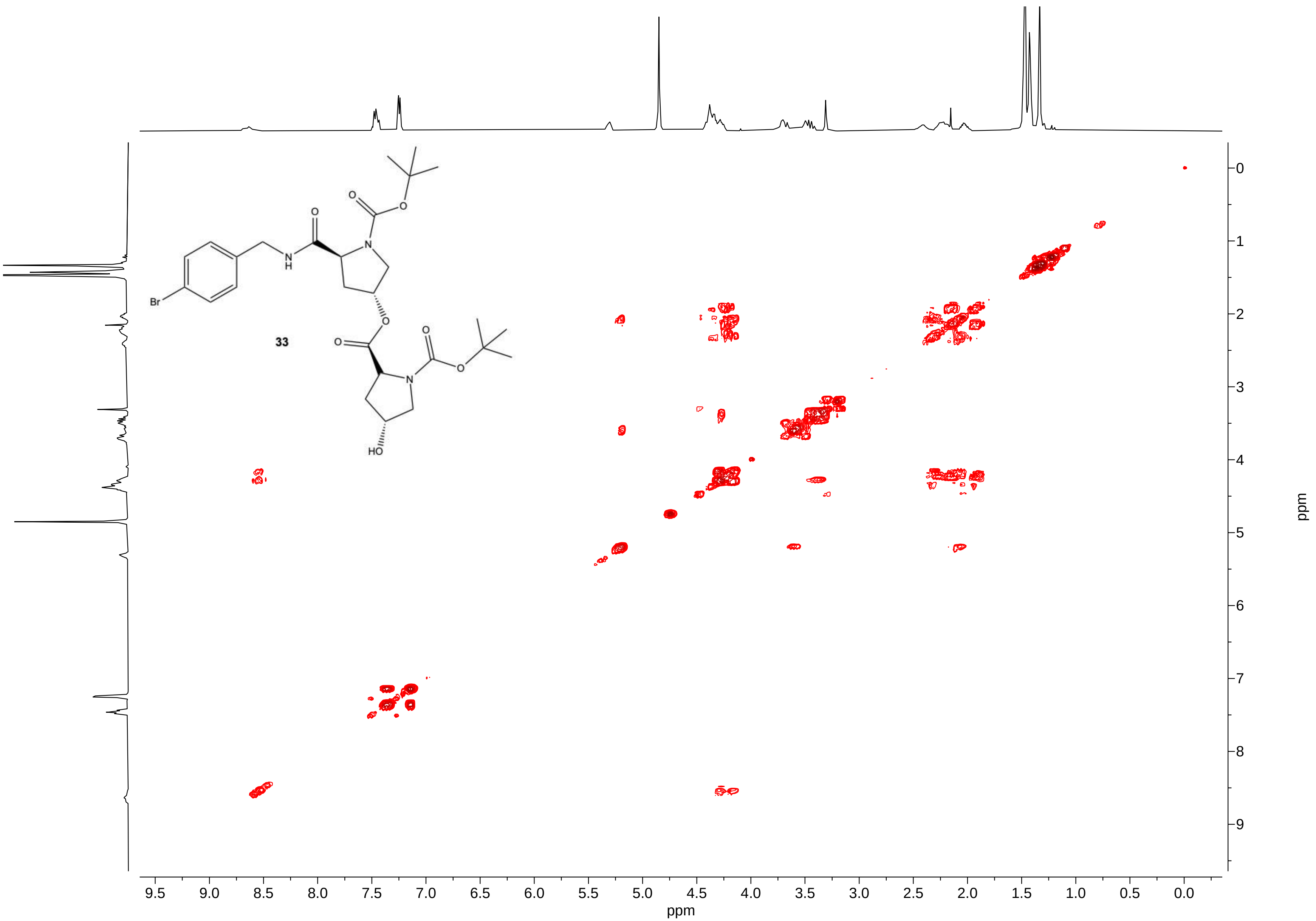

^{1}H-^{1}H COSY NMR (500 MHz, CD_3OD) spectrum of **33**

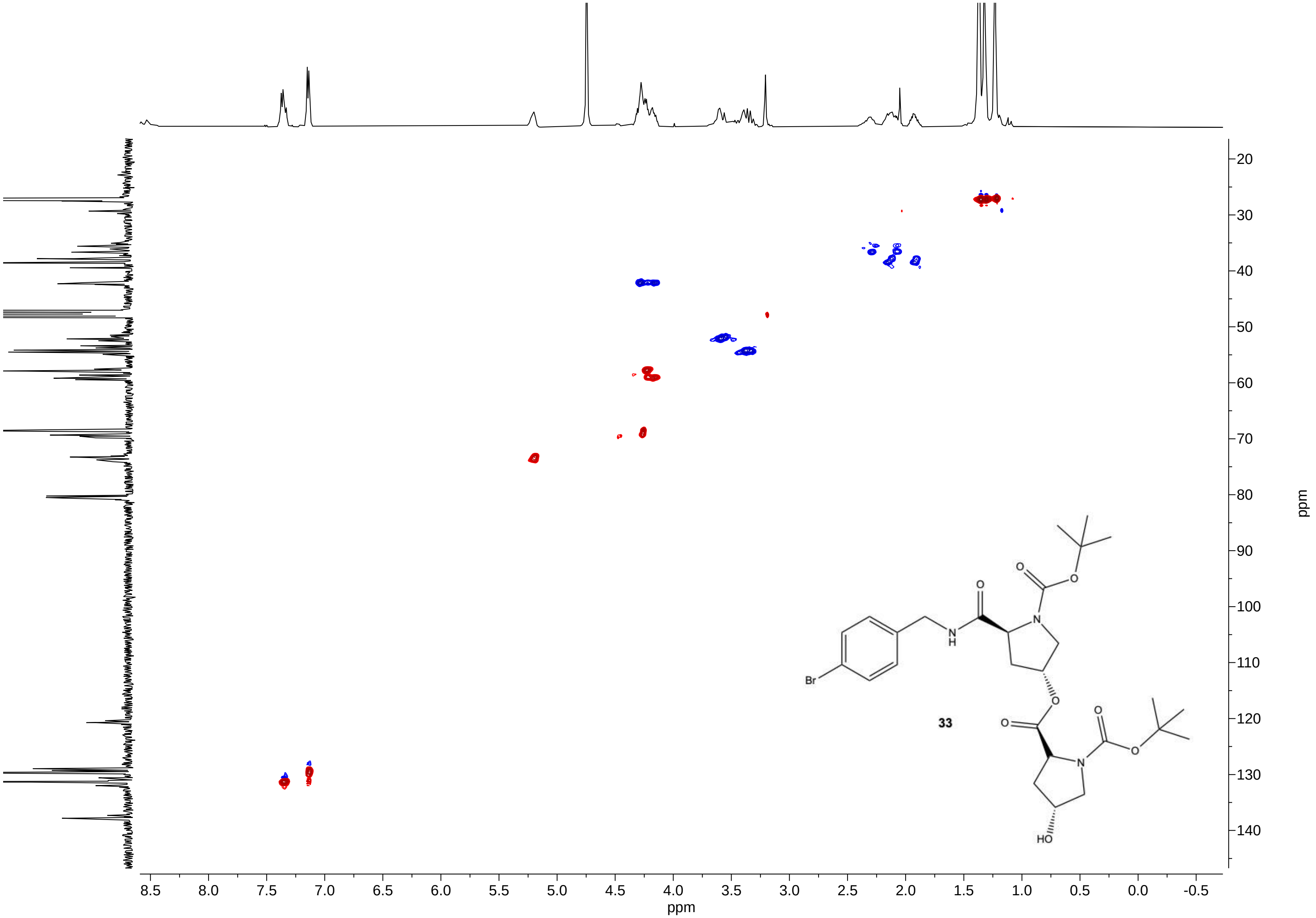

33

^{1}H-^{13}C HSQC NMR (500 MHz, CD_3OD) spectrum of **33**

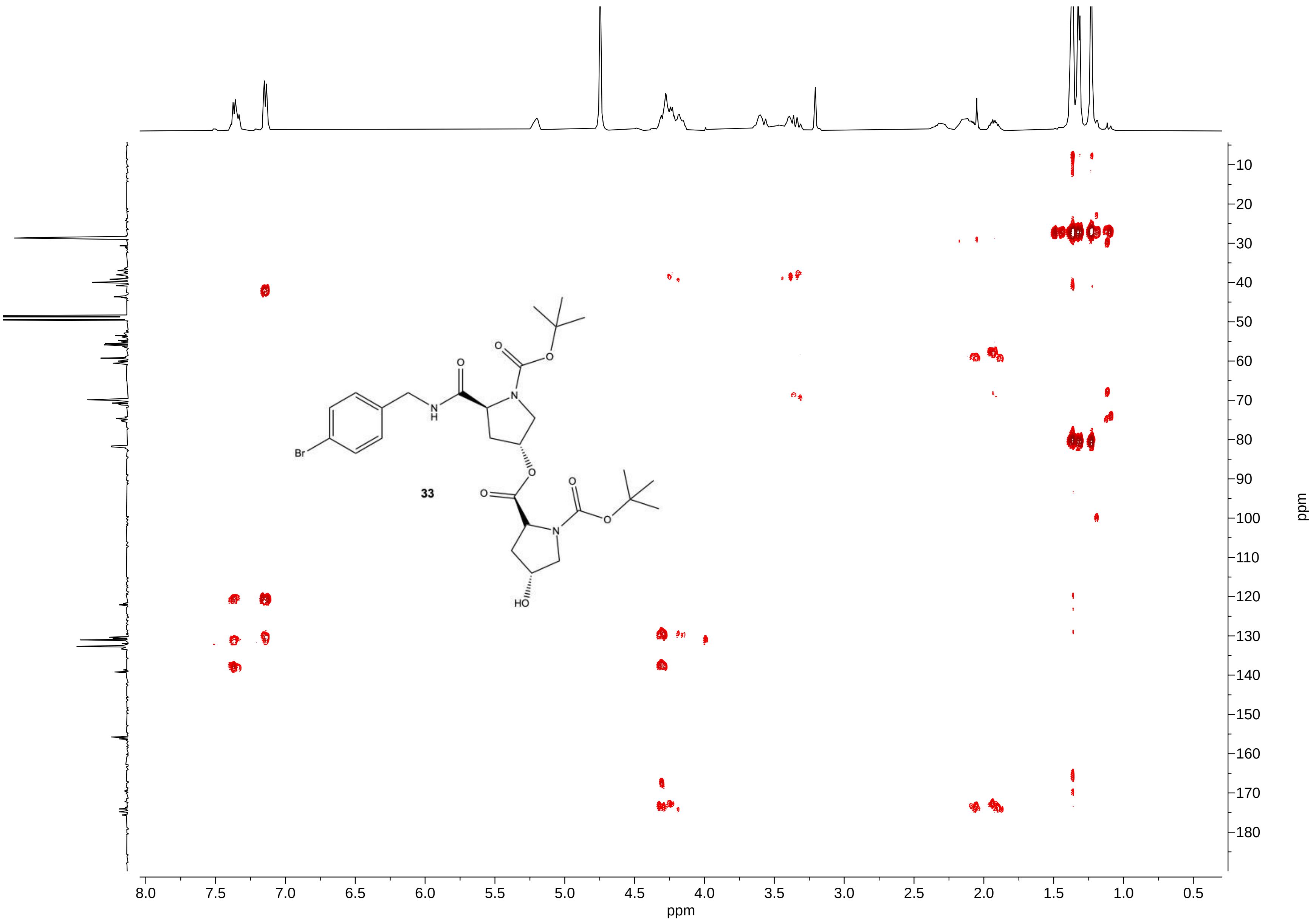

^{1}H-^{13}C HMBC NMR (500 MHz, CD_3OD) spectrum of **33**

7. Quantitative NMR spectra

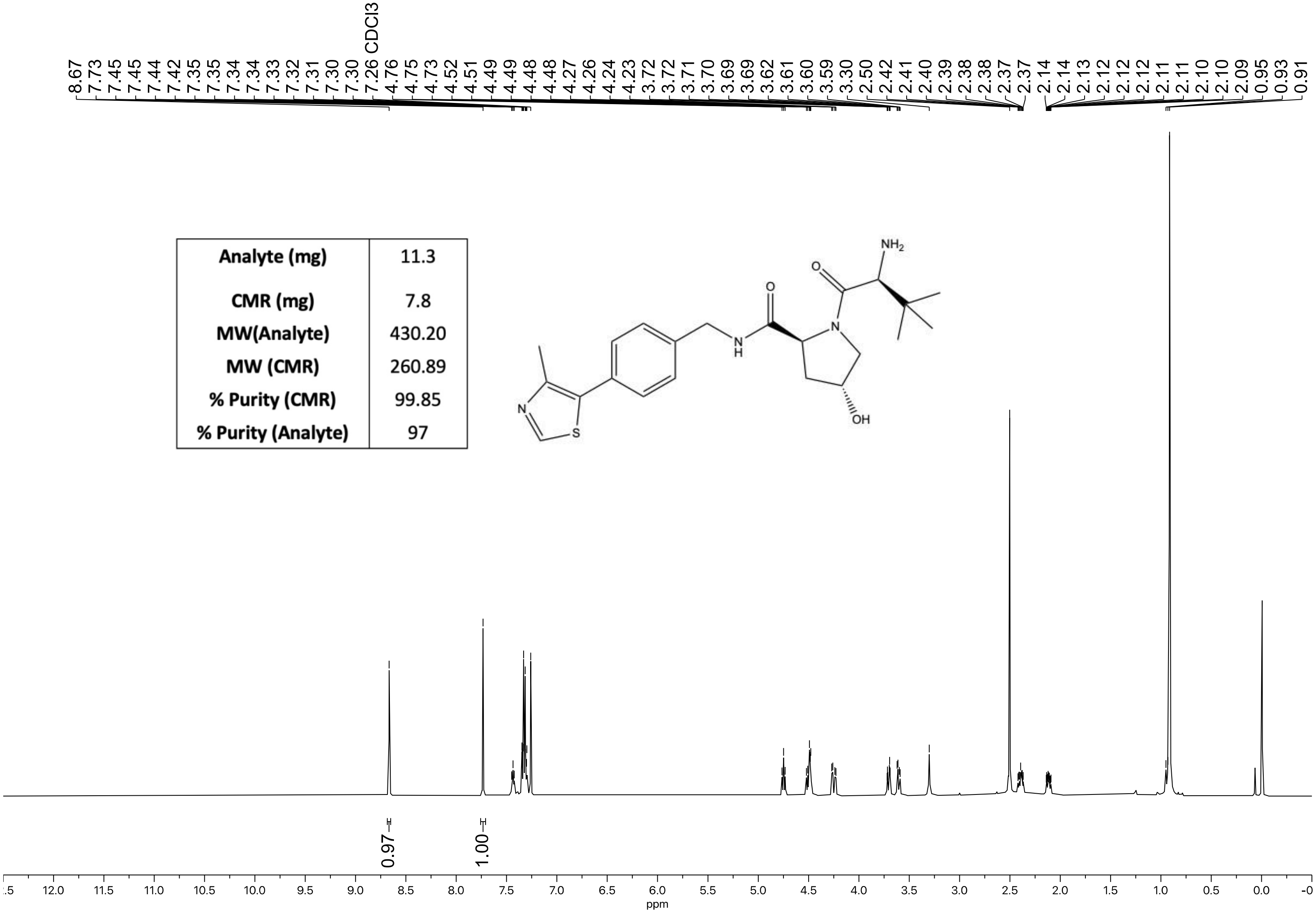

**Analyte (mg)**	11.3
**CMR (mg)**	7.8
**MW(Analyte)**	430.20
**MW (CMR)**	260.89
**% Purity (CMR)**	99.85
**% Purity (Analyte)**	97

qNMR-^{1}H NMR (500 MHz, $CDCl_3$) spectrum of **1**

Analyte (mg)	12.6
CMR (mg)	7.5
MW (Analyte)	444.22
MW (CMR)	260.89
% Purity (CMR)	99.85
% Purity (Analyte)	98

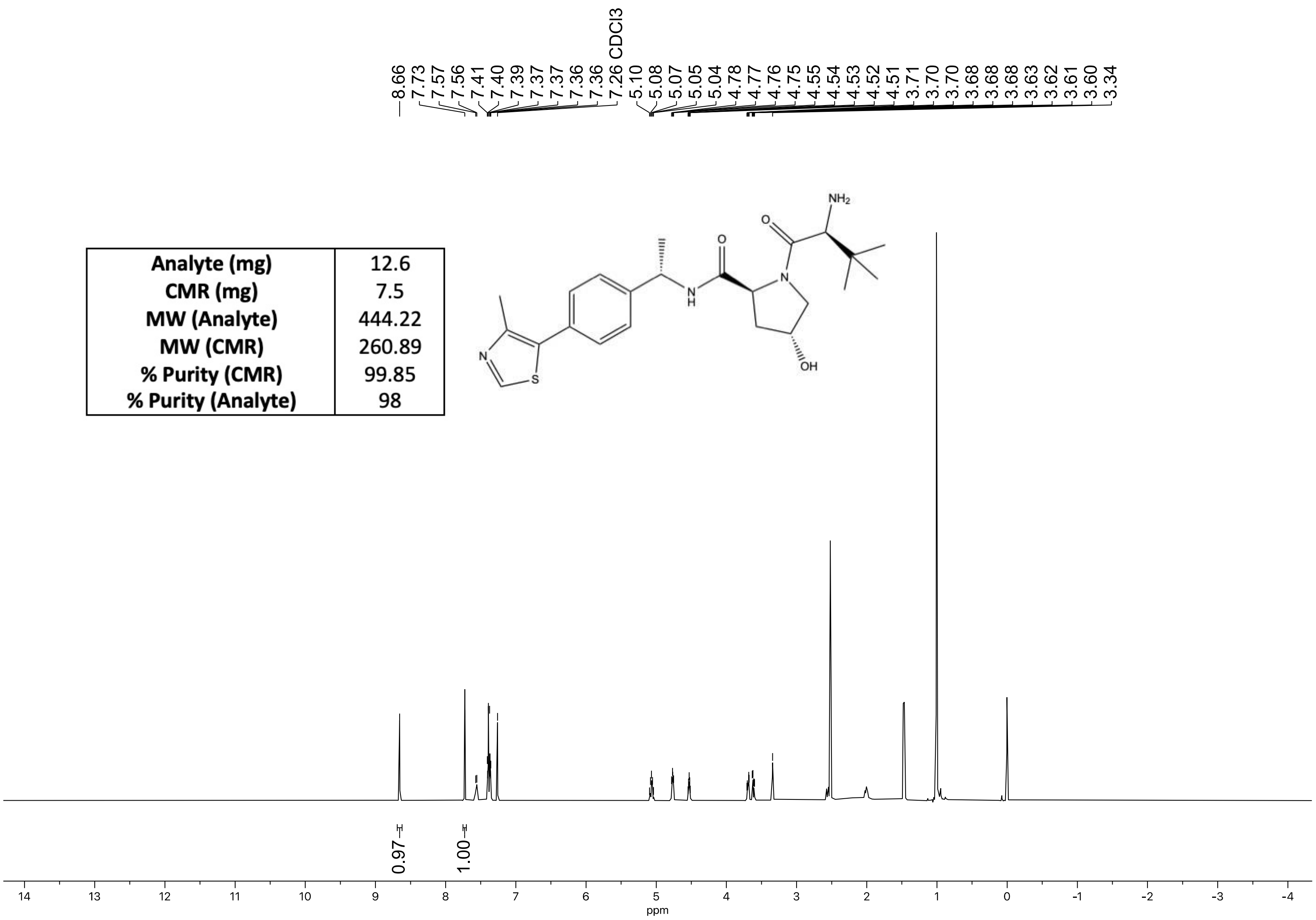

qNMR-^{1}H NMR (500 MHz, $CDCl_3$) spectrum of **2**
